# Supplementary material for: Microtubule Destabilizing Sulfonamides as an Alternative to Taxane-Based Chemotherapy
Source: Int J Mol Sci. 2021 Feb 14;22(4):1907. doi: 10.3390/ijms22041907 (PMC7918738; doi:10.3390/ijms22041907)
Supplement: Supplementary file 1 [file ijms-22-01907-s001.pdf]

Supplementary Materials

Microtubule Destabilizing Sulfonamides as an alternative to taxane-based chemotherapy

Myriam González [1,2,3,†], María Ovejero-Sánchez [2,4,5,†], Alba Vicente-Blázquez [1,2,3], Raquel Álvarez [1,2,3], Ana B. Herrero [2,4,5], Manuel Medarde [1,2,3], Rogelio González-Sarmiento [2,4,5,*] and Rafael Peláez [1,2,3,*]

[1] Laboratorio de Química Orgánica y Farmacéutica, Departamento de Ciencias Farmacéuticas, Facultad de Farmacia, Universidad de Salamanca, Salamanca, 37007, Spain; mygondi@usal.es (M.G.); avicente-blazquez@usal.es (A.V.-B.); raquelalvarez@usal.es (R.A.); medarde@usal.es (M.M.); pelaez@usal.es (R.P.)
[2] Instituto de Investigación Biomédica de Salamanca (IBSAL), Hospital Universitario de Salamanca, Sa-lamanca, 37007, Spain; mygondi@usal.es (M.G.); maría.os@usal.es (M.O.-S); avicenteblazquez@usal.es (A.V.-B.); raquelalvarez@usal.es (R.A.); anah@usal.es (A.B.H.); medarde@usal.es (M.M.); gonza-lez@usal.es (R.G.-S.) pelaez@usal.es (R.P.)
[3] Centro de Investigación de Enfermedades Tropicales de la Universidad de Salamanca (CIETUS), Fa-cultad de Farmacia, Universidad de Salamanca, Salamanca, 37007, Spain; mygondi@usal.es (M.G.); avicenteblazquez@usal.es (A.V.-B.); raquelalvarez@usal.es (R.A.); medarde@usal.es (M.M.); pe-laez@usal.es (R.P.)
[4] Unidad de Medicina Molecular, Departamento de Medicina, Facultad de Medicina, Universidad de Salamanca, Salamanca, 37007, Spain; maría.os@usal.es (M.O.-S); anah@usal.es (A.B.H.); gonza-lez@usal.es (R.G.-S.)
[5] Laboratorio de Diagnóstico en Cáncer Hereditario, Laboratorio 14, Centro de Investigación del Cáncer, Universidad de Salamanca-CSIC, Salamanca, 37007, Spain; maría.os@usal.es (M.O.-S); anah@usal.es (A.B.H.); gonzalez@usal.es (R.G.-S.)
[*] Correspondence: pelaez@usal.es; Tel.: +34-677-554890 (Ext. 1837) (R.P.); gonzalez@usal.es; Tel.: +34-923-294500 (R.G.-S.)
[†] These authors contributed equally to this work.

List of contents:

1) **Figure S1**. Synthetic procedures of new Microtubule Destabilizing Sulfonamides (p. 2).
2) **Table S1**. Antiproliferative activity against HT-29 and sensitivity to MDR pumps (p. 3-4).
3) **Figure S2**. Tubulin Polymerization Assay data. IC_{50} calculation (p. 5).
4) **Methods SP1**. Chemistry. General chemical techniques and chemical synthesis (p. 6-20).
5) **Methods SP2**. ^{1}H NMR and ^{13}C NMR spectra (p. 21-72).

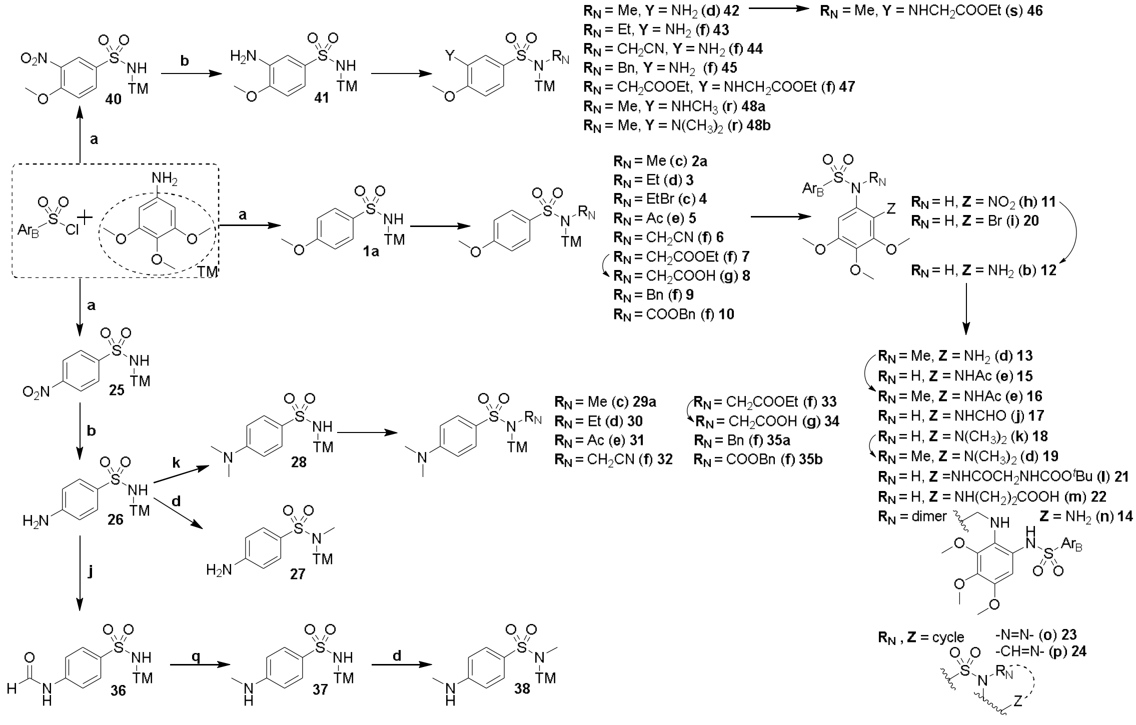

Figure S1. Synthetic procedures of new Microtubule Destabilizing Sulfonamides. Reagents, conditions, and yields: (**a**) Pyridine, CH_2Cl_2, rt, 4-8 h, 90-96% (**b**) H_2, Pd/C, EtOAc, rt, 48-72 h, 82-98% (**c**) R_N-halogen, NaOH or KOH, $n$$Bu_4N^+HSO_4^-$, CH_2Cl_2, rt, 48-72 h, 26-78% (**d**) R_N-halogen, KOH, CH_3CN, rt, 24 h, 63-98% (**e**) Acetic anhydride, pyridine, CH_2Cl_2, rt or reflux, 8-12 h, 61-84% (**f**) R_N-halogen, K_2CO_3, dry DMF, rt, 24-48 h, 40-99% (**g**) KOH, MeOH, rt, 30 min, 63-89% (**h**) *tert*-Butyl nitrite, CH_3CN, 45 °C, 24 h, 60% (**i**) NBS, CH_2Cl_2, rt, 6 h, 43% (**j**) Formic acid, CH_2Cl_2, rt, 24-48 h, 62-82% (**k**) Paraformaldehyde, $NaBH_3CN$, AcOH, MeOH, rt, 72-96 h, 95% (**l**) (*tert*-butoxycarbonyl)glycine, EDCI, 4-DMAP, CH_2Cl_2, rt, 24 h, 53% (**m**) Succinic anhydride, pyridine, CH_2Cl_2, rt or reflux, 24-72 h, 23-35% (**n**) KOH, $n$$Bu_4N^+HSO_4^-$, CH_2Cl_2, rt, 72 h, 35% (**o**) *tert*-Butyl nitrite, CH_3CN, H_2O, AcOH, 0 °C, 24 h, 30% (**p**) Triethyl orthoformate, CH_3CN, reflux, 2 h, 87% (**q**) Trichloroacetic acid, $NaBH_4$, dry THF, 0°C, 24 h, 97% (**r**) $(CH_3)_2SO_4$, K_2CO_3, acetone, reflux, 12 h, 11-32% (**s**) Ethyl 2-bromoacetate, NaI, acetone/THF 1:1, reflux, 48 h, 19%.

Table S1. Chemical structure, antiproliferative activity against the CA-4 resistant cancer cell line HT-29, and sensitivity to MDR pumps of novel Microtubule Destabilizing Sulfonamides (MDS). Compounds have been divided into three different series according to the substituents on the aromatic B ring (Ar_B). TM: 3,4,5-trimethoxyphenyl.

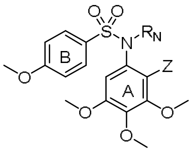

Series 1:			Antiproliferative activity IC_{50} (nM)	Sensivity to MDR pumps IC_{50} (nM)
				HT-29
**Z**	**R_N**	**Compound**	**HT-29**	**Verapamil 10 μM**
H	H	**1a**	897	975
H	SO_2-4-OMePh	**1b**	>1000	n.d. [1]
H	Me	**2a**	143	117
H	CH_2-Dim	**2b**	>1000	n.d.
H	Et	**3**	913	328
H	EtBr	**4**	503	460
H	Ac	**5**	747	683
H	CH_2CN	**6**	230	458
H	CH_2COOEt	**7**	237	398
H	CH_2COOH	**8**	>1000	n.d.
H	Benzyl	**9**	>1000	n.d.
H	COOBenzyl	**10**	>1000	n.d.
NO_2	H	**11**	>1000	n.d.
NH_2	H	**12**	>1000	n.d.
NH_2	Me	**13**	>1000	n.d.
NH_2	CH_2-Dim	**14**	>1000	n.d.
NHAc	H	**15**	>1000	n.d.
NHAc	Me	**16**	>1000	n.d.
NHCHO	H	**17**	>1000	n.d.
$N(CH_3)_2$	H	**18**	>1000	n.d.
$N(CH_3)_2$	Me	**19**	>1000	n.d.
Br	H	**20**	>1000	n.d.
Gly-*t*BOC	H	**21**	>1000	n.d.
Succinic	H	**22**	>1000	n.d.
-N=N-		**23**	>1000	n.d.
-N=CH-		**24**	>1000	n.d.

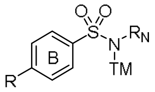

Series 2:			Antiproliferative activity IC_{50} (nM)	Sensivity to MDR pumps IC_{50} (nM)
				HT-29
**R**	**R_N**	**Comp**	**HT-29**	**Verapamil 10 μM**
NO_2	H	**25**	>1000	n.d.
NH_2	H	**26**	>1000	n.d.
NH_2	Me	**27**	>1000	n.d.
$N(CH_3)_2$	H	**28**	887	1170
$N(CH_3)_2$	Me	**29a**	103	43
$N(CH_3)_2$	CH_2-Dim	**29b**	>1000	n.d.
$N(CH_3)_2$	Et	**30**	460	84
$N(CH_3)_2$	Ac	**31**	140	240
$N(CH_3)_2$	CH_2CN	**32**	123	237
$N(CH_3)_2$	CH_2COOEt	**33**	863	850
$N(CH_3)_2$	CH_2COOH	**34**	>1000	n.d.
$N(CH_3)_2$	Benzyl	**35a**	165	287
$N(CH_3)_2$	COOBenzyl	**35b**	690	n.d.
NHCHO	H	**36**	>1000	n.d.
$NHCH_3$	H	**37**	807	n.d.

$NHCH_3$	Me	**38**	59	50
**Series 3:** 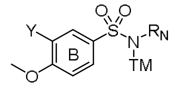			**Antiproliferative activity IC_{50} (nM)**	**Sensivity to MDR pumps IC_{50} (nM)**
**Y**	**R_N**	**Comp**	**HT-29**	**HT-29 Verapamil 10 μM**
NO_2	H	**40**	>1000	n.d.
NH_2	H	**41**	277	260
NH_2	Me	**42**	81	79
NH_2	Et	**43**	63	58
NH_2	CH_2CN	**44**	61	62
NH_2	Benzyl	**45**	300	276
$NHCH_2COOEt$	Me	**46**	113	317
$NHCH_2COOEt$	CH_2COOEt	**47**	>1000	n.d.
$NHCH_3$	Me	**48a**	>1000	n.d.
$N(CH_3)_2$	Me	**48b**	>1000	n.d.
**Paclitaxel** [52]			2.8	n.d.
**Combretastatin A-4**			305	327
**ABT-751**			213	250

1 n.d.: not determined.

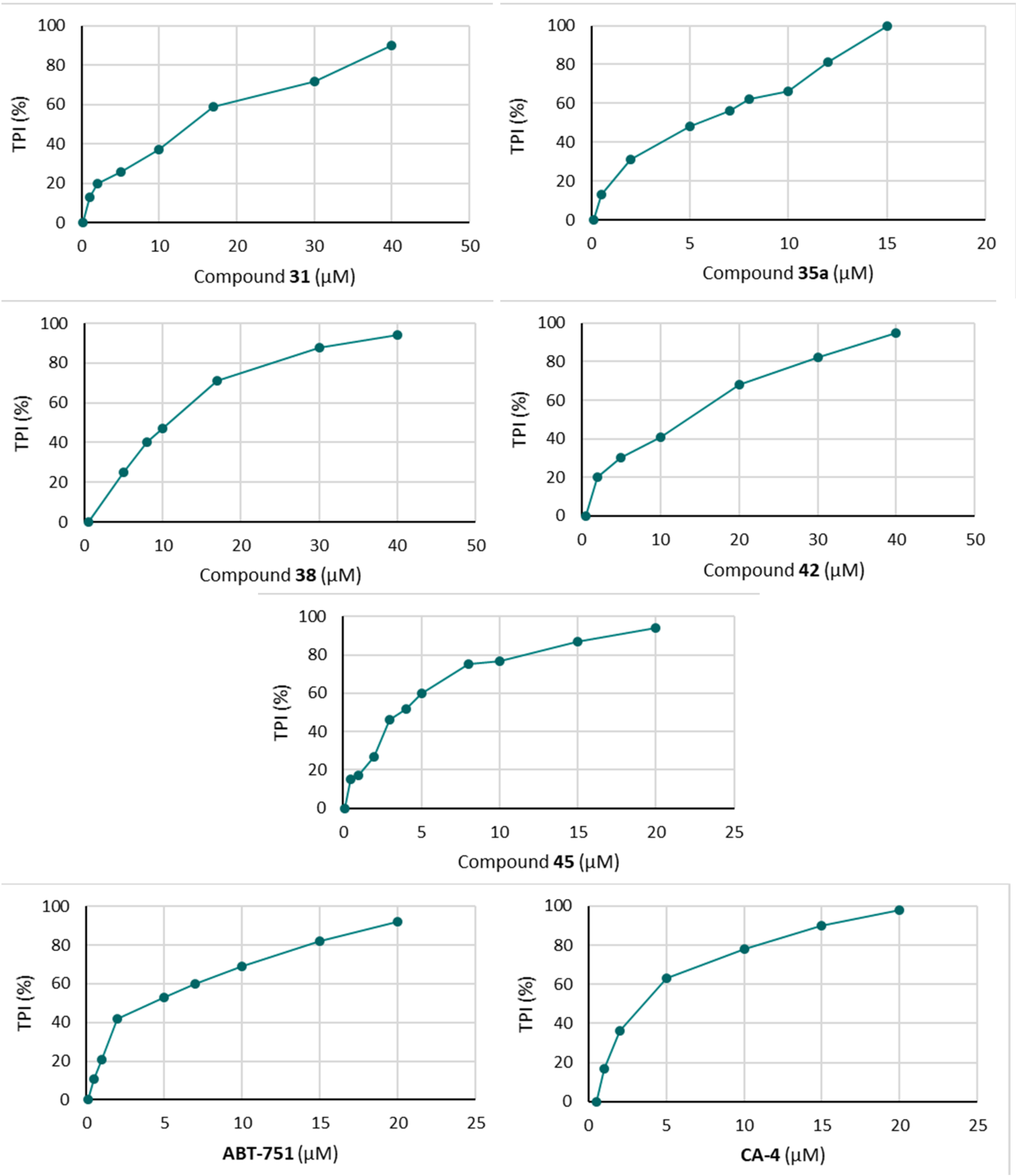

Figure S2. Tubulin Polymerization Assay data. IC_{50} calculation. Tubulin Polymerization Inhibition (TPI) percentages are the average of three independent experiments.

Methods SP1

Chemistry

General chemical techniques

Reagents were used as purchased without further purification. Solvents (EtOAc, DMF, CH_2Cl_2, MeOH, CH_3CN, toluene) were stored over molecular sieves. THF was refluxed with sodium/benzophenone, and hexane was dried by distillation and stored over $CaCl_2$. TLC was performed on precoated silica gel polyester plates (0.25 mm thickness) with a UV fluorescence indicator 254 (Polychrom SI F254). Chromatographic separations were performed on silica gel columns by flash (Kieselgel 40, 0.040-0.063; Merck) chromatography. Melting points were determined on a Buchi 510 apparatus and are uncorrected. ^{1}H NMR and ^{13}C NMR spectra were recorded in $CDCl_3$, CD_3OD, DMSO-D6, or acetone-D6 on a Bruker WP 200-SY spectrometer operating at 200/50 MHz, or a Bruker SY spectrometer at 400/100 MHz. Chemical shifts (δ) are given in ppm downfield from tetramethylsilane and coupling constants (*J* values) are given in Hz. IR spectra in KBr disk were run on a Nicolet Impact 410 Spectrophotometer. A hybrid QSTAR XL quadrupole/time of flight spectrometer was used for HRMS analyses. GC-MS spectra were performed using a Hewlett-Packard 5890 series II mass detector. A Helios-*α* UV-320 from Thermo-Spectronic was used for UV spectra.

Chemical synthesis

4-Methoxy-*N*-(3,4,5-trimethoxyphenyl)benzenesulfonamide (**1a**) and 4-methoxy-*N*-((4-methoxyphenyl)sulfonyl)-*N*-(3,4,5-trimethoxyphenyl)benzenesulfonamide (**1b**). 3,4,5-Trimethoxyaniline (2.64 g, 14.45 mmol) was added to a solution of 4-methoxybenzenesulfonyl chloride (2.97 g, 14.45 mmol) in CH_2Cl_2 (100 mL) and triethylamine (6 mL), and stirred at room temperature. After 4 h, the reaction was treated with 2N HCl and 5% $NaHCO_3$, washed with brine to neutrality, dried over anhydrous Na_2SO_4, and concentrated under vacuum. Successive crystallizations in EtOH and EtOAc provided compounds **1a** (3.58 g, 10.14 mmol, 70%) and **1b** (0.95 g, 1.82 mmol, 25%), respectively. Another synthetic methodology was performed to obtain **1a** in better yield as a single product. 4-Methoxybenzenesulfonyl chloride (2.22 g, 10.76 mmol) dissolved in CH_2Cl_2 (50 mL) was dropwise added to a solution of 3,4,5-trimethoxyaniline (1.97 g, 10.76 mmol) in CH_2Cl_2 (100 mL) and pyridine (3 mL). The mixture was stirred at room temperature for 4 h. The reaction was then treated with 2N HCl and 5% $NaHCO_3$, washed with brine, dried over anhydrous Na_2SO_4, and concentrated under vacuum yielding 3.65 g (10.34 mmol, 96%) of **1a**. **1a:** M.p.: 115-116 °C (EtOH). IR (KBr): 3283, 1601, 1578, 1497, 1335 cm^{-1}. ^{1}H NMR (400 MHz, $CDCl_3$): δ 3.75 (6H, *s*), 3.78 (3H, *s*), 3.84 (3H, *s*), 6.23 (1H, *s*), 6.27 (2H, *s*), 6.91 (2H, *d*, *J* = 9.2), 7.69 (2H, *d*, *J* = 9.2). ^{13}C NMR (100 MHz, $CDCl_3$): δ 56.1 (CH_3), 56.4 ($2CH_3$), 61.3 (CH_3), 99.7 (2CH), 114.5 (2CH), 130.3 (2CH), 131.4 (C), 133.2 (C), 135.7 (C), 153.8 (2C), 163.5 (C). HRMS ($C_{16}H_{19}NO_6S + H^+$): calcd 354.1006 ($M + H^+$), found 354.1009. **1b:** M.p.: 192-193 °C (EtOAc). IR (KBr): 1592, 1576, 1495, 1376, 1356 cm^{-1}. ^{1}H NMR (400 MHz, $CDCl_3$): δ 3.68 (6H, *s*), 3.87 (3H, *s*), 3.91 (6H, *s*), 6.18 (2H, *s*), 7.00 (4H, *d*, *J* = 8.8), 7.90 (4H, *d*, *J* = 8.8). ^{13}C NMR (100 MHz, $CDCl_3$): δ 55.7 ($2CH_3$), 56.0 ($2CH_3$), 60.9 (CH_3), 108.9 (2CH), 114.0 (4CH), 129.5 (C), 129.6 (2C), 130.9 (4CH), 139.5 (C), 153.0 (2C), 163.9 (2C). HRMS ($C_{23}H_{25}NO_9S_2 + H^+$): calcd 524.1043 ($M + H^+$), found 542.1060.

4-Methoxy-*N*-methyl-*N*-(3,4,5-trimethoxyphenyl)benzenesulfonamide (**2a**) and *N*,*N*'-methylenebis(4-methoxy-*N*-(3,4,5-trimethoxyphenyl)benzenesulfonamide) (**2b**). CH_3I (81 μL, 1.30 mmol) was added to a mixture of **1a** (229 mg, 0.65 mmol), NaOH (30 mg, 1.30 mmol), and *n*$Bu_4N^+HSO_4^-$ (260 mg, 1.30 mmol) in CH_2Cl_2 (100 mL). The reaction was stirred at room temperature under nitrogen atmosphere for 3 days. The solution was poured onto brine, extracted with CH_2Cl_2, and then dried over Na_2SO_4. After concentration, the residue was purified by silica gel column chromatography (hexane/EtOAc, 6:4) to give **2a** (187 mg, 0.51 mmol, 78%) and **2b** (39 mg, 0.05 mmol, 17%). **2a:** M.p.: 123-124 °C (CH_2Cl_2/Hexane). IR (KBr): 1595, 1468, 1336 cm^{-1}. ^1H NMR (400 MHz, $CDCl_3$): δ 3.05 (3H, *s*), 3.66 (6H, *s*), 3.76 (3H, *s*), 3.79 (3H, *s*), 6.21 (2H, *s*), 6.87 (2H, *d, J* = 8.8), 7.48 (2H, *d, J* = 8.8). ^{13}C NMR (100 MHz, $CDCl_3$): δ 38.5 (CH_3), 55.6 (CH_3), 56.1 ($2CH_3$), 60.9 (CH_3), 104.5 (2CH), 113.8 (2CH), 128.0 (C), 130.1 (2CH), 137.3 (C), 137.4 (C), 152.9 (2C), 163.0 (C). HRMS ($C_{17}H_{21}NO_6S$ + H^+): calcd 368.1162 (M + H^+), found 368.1154. **2b:** M.p.: 178-179 °C (EtOAc). IR (KBr): 1594, 1464, 1349 cm^{-1}. ^1H NMR (400 MHz, $CDCl_3$): δ 3.63 (12H, *s*), 3.83 (6H, *s*), 3.84 (3H, *s*), 5.52 (2H, *s*), 6.11 (4H, *s*), 6.83 (4H, *d, J* = 8.8), 7.34 (4H, *d, J* = 8.8). ^{13}C NMR (100 MHz, $CDCl_3$): δ 55.6 ($2CH_3$), 55.9 ($4CH_3$), 60.9 ($2CH_3$), 65.7 (CH_2), 106.9 (4CH), 113.8 (4CH), 129.7 (4CH), 130.4 (2C), 132.9 (2C), 138.0 (2C), 152.9 (4C), 163.0 (2C). HRMS ($C_{33}H_{38}N_2O_{12}S_2$ + H^+): calcd 719.1939 (M + H^+), found 719.1959.

N-ethyl-4-methoxy-*N*-(3,4,5-trimethoxyphenyl)benzenesulfonamide (**3**). 101 mg (0.28 mmol) of compound **1a** were dissolved in CH_3CN (50 mL) and 39 mg (0.56 mmol) of crushed KOH were added. After 30 min stirring at room temperature, 42.7 μL (0.56 mmol) of bromoethane were added to the solution. The reaction mixture was stirred at room temperature for 24 h. The mixture was evaporated to dryness. The residue was diluted with water, and the solution was extracted with EtOAc. The extract was dried over Na_2SO_4 and evaporated to dryness yielding 103 mg (0.27 mmol, 94%) of **3**. The residue was crystallized from CH_2Cl_2/hexane to give 78 mg (0.20 mmol, 71%) of compound **3**. M.p.: 140-143 °C (CH_2Cl_2/Hexane). ^1H NMR (400 MHz, $CDCl_3$): δ 1.09 (3H, *t, J* = 7.2), 3.54 (2H, *q, J* = 7.2), 3.72 (6H, *s*), 3.84 (3H, *s*), 3.86 (3H, *s*), 6.23 (2H, *s*), 6.94 (2H, *s, J* = 8.8), 7.60 (2H, *d, J* = 8.8). ^{13}C NMR (100 MHz, $CDCl_3$): δ 14.1 (CH_3), 45.7 (CH_2), 55.5 (CH_3), 56.1 ($2CH_3$), 60.9 (CH_3), 106.5 (2CH), 113.8 (2CH), 129.9 (2CH), 130.0 (C), 134.5 (C), 137.7 (C), 153.0 (2C), 162.9 (C). HRMS ($C_{18}H_{23}NO_6S$ + H^+): calcd 382.1319 (M + H^+), found 382.1322.

N-(2-bromoethyl)-4-methoxy-*N*-(3,4,5-trimethoxyphenyl)benzenesulfonamide (**4**). A mixture of **1a** (161 mg, 0.45 mmol), KOH (51 mg, 0.91 mmol), *n*$Bu_4N^+HSO_4^-$ (309 mg, 0.91 mmol), and 1,2-dibromoethane (79 μL, 0.91 mmol) in CH_2Cl_2 (100 mL) was stirred at room temperature for 3 days. The reaction was washed with brine, extracted with CH_2Cl_2, and dried over Na_2SO_4. After concentration, the crude material was purified by column chromatography using hexane/EtOAc 6:4 as eluants to give **4** (55 mg, 0.12 mmol, 26%) and **2b** (121 mg, 0.17 mmol, 74%). M.p.: 101-102 °C (CH_2Cl_2/Hexane). IR (KBr): 1593, 1498, 1352 cm^{-1}. ^1H NMR (400 MHz, $CDCl_3$): δ 3.41 (2H, *t, J* = 7.2), 3.57 (2H, *t, J* = 7.2), 3.72 (6H, *s*), 3.84 (3H, *s*), 3.87 (3H, *s*), 6.25 (2H, *s*), 6.95 (2H, *d, J* = 8.8), 7.61 (2H, *d, J* = 8.8). ^{13}C NMR (100 MHz, $CDCl_3$): δ 41.3 (CH_2), 52.7 (CH_2), 55.6 (CH_3), 56.1 ($2CH_3$), 60.8 (CH_3), 106.5 (2CH), 113.9 (2CH), 129.7 (C), 129.9 (2CH), 134.4 (C), 138.1 (C), 153.2 (2C), 163.1 (C). HRMS ($C_{18}H_{22}BrNO_6S$ + H^+): calcd 460.0424 and 462.0404 (M + H^+), found 460.0394 and 462.0382.

N-((4-methoxyphenyl)sulfonyl)-*N*-(3,4,5-trimethoxyphenyl)acetamide (**5**). Acetic anhydride (304 μL, 323 mmol) was added to a solution of **1a** (114 mg, 0.32 mmol) in CH_2Cl_2 (50 mL) and pyridine (1 mL). The reaction mixture was heated under reflux for 12 h. After cooling, the mixture was poured onto ice. The organic layer was separated, washed with 2N HCl, 5% $NaHCO_3$, and brine, dried (Na_2SO_4), filtered, and concentrated under vacuum to yield 107 mg (0.27 mmol, 83%) of compound **5**. The product was purified by crystallization in CH_2Cl_2/Hexane (50 mg, 0.13 mmol, 39%). M.p.: 190-195 °C (CH_2Cl_2/Hexane). ^{1}H NMR (400 MHz, $CDCl_3$): δ 1.86 (3H, *s*), 3.76 (6H, *s*), 3.81 (3H, *s*), 3.82 (3H, *s*), 6.37 (2H, *s*), 6.93 (2H, *d, J* = 8.8), 7.92 (2H, *d, J* = 8.8). ^{13}C NMR (100 MHz, $CDCl_3$): δ 24.7 (CH_3), 55.7 (CH_3), 56.3 (2CH_3), 61.0 (CH_3), 107.2 (2CH), 113.8 (2CH), 130.2 (C), 131.5 (2CH), 132.2 (C), 139.2 (C), 153.7 (2C), 163.9 (C), 170.2 (C). HRMS ($C_{18}H_{21}NO_7S + H^+$): calcd 396.1111 (M + H^+), found 396.1126.

N-(cyanomethyl)-4-methoxy-*N*-(3,4,5-trimethoxyphenyl)benzenesulfonamide (**6**). 2-Chloroacetonitrile (37 μL, 0.59 mmol) was added after 30 min to a stirred solution of **1a** (104 mg, 0.29 mmol) and K_2CO_3 (81 mg, 0.59 mmol) in DMF (5 mL). After 24 h, the reaction mixture was dried under vacuum, re-dissolved in EtOAc, and washed with brine. After drying over Na_2SO_4 and removal of the solvent, the residue was purified by preparative TLC (hexane/EtOAc 1:1) to give **6** (57 mg, 0.15 mmol, 49%). ^{1}H NMR (400 MHz, $CDCl_3$): δ 3.67 (6H, *s*), 3.79 (3H, *s*), 3.82 (3H, *s*), 4.49 (2H, *s*), 6.34 (2H, *s*), 6.92 (2H, *d, J* = 8.8), 7.62 (2H, *d, J* = 8.8). ^{13}C NMR (100 MHz, $CDCl_3$): δ 40.1 (CH_2), 56.1 (2CH_3), 56.5 (CH_3), 61.3 (CH_3), 106.3 (2CH), 114.5 (2CH), 115.5 (C), 129.1 (C), 130.8 (2CH), 134.3 (C), 139.0 (C), 153.8 (2C), 164.1 (C). HRMS ($C_{18}H_{20}N_2O_6S + H^+$): calcd 393.1115 (M + H^+), found 393.1097.

Ethyl *N*-((4-methoxyphenyl)sulfonyl)-*N*-(3,4,5-trimethoxyphenyl)glycinate (**7**). K_2CO_3 (78 mg, 0.56 mmol) was added to a solution of **1a** (100 mg, 0.28 mmol) in DMF (5 mL), and the resulting mixture was stirred for 30 min. Then, ethyl 2-bromoacetate (63 μL, 0.56 mmol) was added. After 24 h, the solvent was evaporated to dryness and the residue was dissolved in brine and extracted with EtOAc. The organics were dried over Na_2SO_4 before concentration under reduced pressure to yield compound **7** (121 mg, 0.28 mmol, 97%). The crude product obtained was recrystallized from CH_2Cl_2/Hexane (51 mg, 0.12 mmol, 41%). M.p.: 123-127 °C (CH_2Cl_2/Hexane). ^{1}H NMR (400 MHz, $CDCl_3$): δ 1.25 (3H, *t, J* = 7.2), 3.71 (6H, *s*), 3.83 (3H, *s*), 3.86 (3H, *s*), 4.17 (2H, *q, J* = 7.2), 4.37 (2H, *s*), 6.42 (2H, *s*), 6.93 (2H, *d, J* = 8.8), 7.67 (2H, *d, J* = 8.8). ^{13}C NMR (100 MHz, $CDCl_3$): δ 14.1 (CH_3), 52.9 (CH_2), 55.6 (CH_3), 56.0 (2CH_3), 60.8 (CH_2), 61.4 (CH_3), 106.4 (2CH), 113.7 (2CH), 130.1 (2CH), 130.4 (C), 135.3 (C), 137.8 (C), 153.0 (2C), 163.1 (C), 168.9 (C). HRMS ($C_{20}H_{25}NO_8S + H^+$): calcd 440.1374 (M + H^+), found 440.1363.

N-((4-methoxyphenyl)sulfonyl)-*N*-(3,4,5-trimethoxyphenyl)glycine (**8**). Compound **7** (91 mg 0.21 mmol) was diluted with methanolic KOH for saponification. After 30 min stirring at room temperature, the solution was acidified with 2N HCl and extracted with EtOAc. The extract was washed with brine, dried over Na_2SO_4, and evaporated to dryness to afford 77 mg (0.19 mmol, 89%) of the desired compound (**8**). The residue was purified by crystallization in CH_2Cl_2/Hexane (54 mg, 0.13 mmol, 63%). M.p.: 151-158 °C (CH_2Cl_2/Hexane). ^{1}H NMR (400 MHz, $CDCl_3$): δ 3.71 (6H, *s*), 3.82 (3H, *s*), 3.86 (3H, *s*), 4.41 (2H, *s*), 6.39 (2H, *s*), 6.94 (2H, *d, J* = 8.8), 7.65 (2H, *d, J* = 8.8). ^{13}C NMR (100 MHz, $CDCl_3$): δ 52.7 (CH_2), 55.7 (CH_3), 56.1 (2CH_3), 60.8 (CH_3), 106.3 (2CH), 113.8 (2CH), 130.0 (C), 130.1

(C), 135.2 (2CH), 137.9 (C), 153.1 (2C), 163.2 (C), 174.0 (C). HRMS ($C_{18}H_{21}NO_8S + H^+$): calcd 412.1061 ($M + H^+$), found 412.1053.

N-benzyl-4-methoxy-*N*-(3,4,5-trimethoxyphenyl)benzenesulfonamide (**9**). A solution of **1a** (80 mg, 0.22 mmol) and K_2CO_3 (60 mg, 0.44 mmol) in DMF (5 mL) was stirred for 30 min at room temperature. Then, benzyl chloride (31.5 µL, 0.27 mmol) was added and the reaction mixture was stirred for 24 h. When completed, solvent was removed under vacuum and the residue was washed with brine, extracted with EtOAc, dried (Na_2SO_4), filtered, and evaporated to dryness to afford 93 mg (0.21 mmol, 93%) of **9.** The crude reaction product was purified by crystallization in methanol (85 mg, 0.19 mmol, 85%). M.p.: 170-171 °C (MeOH). ^{1}H NMR (400 MHz, $CDCl_3$): δ 3.60 (6H, *s*), 3.77 (3H, *s*), 3.86 (3H, *s*), 4.64 (2H, *s*), 6.10 (2H, *s*), 6.96 (2H, *d, J* = 8.8), 7.22 (5H, *bs*), 7.65 (2H, *d, J* = 8.8). ^{13}C NMR (100 MHz, $CDCl_3$): δ 55.2 (CH_2), 55.6 (CH_3), 56.0 ($2CH_3$), 60.8 (CH_3), 106.7 (2CH), 113.9 (2CH), 127.6 (CH), 128.3 (2CH), 128.7 (2CH), 129.9 (2CH), 130.4 (C), 134.6 (C), 136.1 (C), 137.7 (C), 152.8 (2C), 163.0 (C). HRMS ($C_{23}H_{25}NO_6S + Na^+$): calcd 466.1295 ($M + Na^+$), found 466.1284.

Benzyl ((4-methoxyphenyl)sulfonyl)(3,4,5-trimethoxyphenyl)carbamate (**10**). K_2CO_3 (70 mg, 0.51 mmol) and benzyl chloroformate (43.6 µL, 0.30 mmol) were added to a solution of **1a** (90 mg, 0.25 mmol) in DMF (5 mL), and the solution was stirred at room temperature for 24 h. The reaction mixture was evaporated and re-dissolved in EtOAc, washed with brine, and dried over Na_2SO_4. After concentration, the residue was purified by crystallization in MeOH to give product **10** (30 mg, 0.06 mmol, 24%). M.p.: 179-184 °C (MeOH). ^{1}H NMR (400 MHz, $CDCl_3$): δ 3.80 (6H, *s*), 3.88 (6H, *s*), 5.11 (2H, *s*), 6.43 (2H, *s*), 6.93 (2H, *d, J* = 8.8), 7.17 (2H, *m*), 7.30 (3H, *m*), 7.89 (2H, *d, J* = 8.8). ^{13}C NMR (100 MHz, $CDCl_3$): δ 55.7 (CH_3), 56.2 ($2CH_3$), 60.9 (CH_3), 68.7 (CH_2), 107.1 (2CH), 113.9 (2CH), 127.9 (2CH), 128.4 (2CH), 128.5 (CH), 130.2 (C), 131.2 (2CH), 131.3 (C), 134.7 (C), 138.8 (C), 152.2 (C), 153.3 (2C), 163.8 (C). HRMS ($C_{24}H_{25}NO_8S + Na^+$): calcd 510.1193 ($M + Na^+$), found 510.1193.

4-Methoxy-*N*-(3,4,5-trimethoxy-2-nitrophenyl)benzenesulfonamide (**11**). *tert*-Butyl nitrite (257 µL, 1.94 mmol) was added to a solution of **1a** (1.37 g, 3.89 mmol) in CH_3CN (50 mL), and stirred at 45 °C. After 1 h, additional 1.94 mmol *tert*-butyl nitrite was added to the reaction mixture and it was stirred at 45 °C for 24 h. The mixture was poured onto ice and basified with 5% $NaHCO_3$, and extracted with EtOAc. The organic layers were washed with brine, dried over Na_2SO_4, and concentrated under vacuum. The residue was purified by silica gel column chromatography (hexane/EtOAc 6:4) to give pure product **11** (924 mg, 2.32 mmol, 60%). M.p.: 122-123 °C (CH_2Cl_2/Hexane). IR (KBr): 3268, 1597, 1495, 876 cm^{-1}. ^{1}H NMR (400 MHz, $CDCl_3$): δ 3.82 (3H, *s*), 3.84 (3H, *s*), 3.89 (3H, *s*), 3.94 (3H, *s*), 6.89 (2H, *d, J* = 9.2), 7.05 (1H, *s*), 7.60 (2H, *d, J* = 9.2), 8.08 (1H, *s*). ^{13}C NMR (100 MHz, $CDCl_3$): δ 55.6 (CH_3), 56.4 (CH_3), 61.1 (CH_3), 62.3 (CH_3), 102.4 (CH), 114.5 (2CH), 127.5 (C), 129.1 (2CH), 129.5 (C), 131.9 (C), 140.2 (C), 148.1 (C), 156.7 (C), 163.6 (C). HRMS ($C_{16}H_{18}N_2O_8S + H^+$): calcd 399.0857 ($M^+ + H^+$), found 399.0855.

N-(2-amino-3,4,5-trimethoxyphenyl)-4-methoxybenzenesulfonamide (**12**). A mixture of **11** (907 mg, 2.28 mmol), 10 mg of Pd/C, and 125 mL of EtOAc was maintained under low hydrogen pressure and stirred at room temperature. After 48 h, uptake of H_2 was completed and the solution was filtered through Celite. The filtrate was concentrated under vacuum to give product **12** (823 mg, 2.24 mmol, 98%). The residue was purified

by crystallization in CH_2Cl_2/Hexane (670 mg, 1.82 mmol, 80%). M.p.: 119-120 °C (CH_2Cl_2/Hexane). IR (KBr): 3469, 3377, 1595, 839 cm^{-1}. ^1H NMR (400 MHz, $CDCl_3$): δ 3.47 (3H, *s*), 3.77 (6H, *s*), 3.79 (3H, *s*), 5.82 (1H, *s*), 5.92 (1H, *s*), 6.87 (2H, *d, J* = 9.2), 7.62 (2H, *d, J* = 9.2). ^{13}C NMR (100 MHz, $CDCl_3$): δ 55.6 (CH_3), 56.1 (CH_3), 60.5 (CH_3), 60.8 (CH_3), 107.7 (CH), 114.0 (2CH), 116.5 (C), 129.6 (2CH), 130.6 (C), 132.5 (C), 141.9 (C), 142.3 (C), 144.9 (C), 163.1 (C). HRMS ($C_{16}H_{21}N_2O_6S + H^+$): calcd 369.1115 ($M + H^+$), found 369.1114.

N-(2-amino-3,4,5-trimethoxyphenyl)-4-methoxy-*N*-methylbenzenesulfonamide (**13**). CH_3I (69 µL, 1.10 mmol) was added to 202 mg (0.55 mmol) of **12** and 61 mg (1.10 mmol) of KOH in CH_3CN, and stirred for 24 h. The CH_3CN solution was concentrated under reduced pressure, the residue dissolved in EtOAc and washed with brine. The organics were dried over Na_2SO_4, filtered, and evaporated. The crude material was purified by column chromatography using hexane/EtOAc 7:3 as eluant to give 169 mg (0.44 mmol, 80%) of **13.** ^1H NMR (400 MHz, $CDCl_3$): δ 3.10 (3H, *s*), 3.47 (3H, *s*), 3.86 (3H, *s*), 3.87 (3H, *s*), 3.88 (3H, *s*), 5.69 (1H, *s*), 6.97 (2H, *d, J* = 8.4), 7.66 (2H, *d, J* = 8.4). ^{13}C NMR (100 MHz, $CDCl_3$): δ 38.7 (CH_3), 55.6 (CH_3), 56.3 (CH_3), 60.4 (CH_3), 60.8 (CH_3), 106.6 (CH), 113.9 (2CH), 121.5 (C), 128.7 (C), 130.3 (2CH), 134.8 (C), 141.7 (C), 142.9 (C), 144.4 (C), 163.1 (C). HRMS ($C_{17}H_{22}N_2O_6S + H^+$): calcd 383.1271 ($M + H^+$), found 383.1271.

N-(2-amino-3,4,5-trimethoxyphenyl)-4-methoxy-*N*-(((2,3,4-trimethoxy-6-((4-methoxyphenyl)sulfonamido)phenyl)amino)methyl)benzenesulfonamide (**14**). A mixture of **12** (110 mg, 0.30 mmol), KOH (33 mg, 0.60 mmol), and $nBu_4N^+HSO_4^-$ (203 mg, 0.60 mmol) in CH_2Cl_2 (100 mL) was stirred at room temperature for 3 days. The reaction was washed with brine, extracted with CH_2Cl_2, and dried over Na_2SO_4. After concentration, the crude material was purified by column chromatography using hexane/EtOAc 6:4 as eluant to give **14** (35 mg, 0.05 mmol, 31%). ^1H NMR (400 MHz, $CDCl_3$): δ 3.35 (3H, *s*), 3.47 (3H, *s*), 3.74 (3H, *s*), 3.82 (3H, *s*), 3.84 (3H, *s*), 3.85 (3H, *s*), 3.89 (3H, *s*), 3.93 (3H, *s*), 5.25 (1H, *d, J* = 13.6), 5.56 (1H, *d, J* = 13.6), 5.73 (1H, *s*), 5.88 (1H, *s*), 6.85 (2H, *d, J* = 8.8), 6.87 (2H, *d, J* = 9.2), 7.43 (2H, *d, J* = 9.2), 7.54 (2H, *d, J* = 8.8). ^{13}C NMR (100 MHz, $CDCl_3$): δ 55.6 (CH_3), 55.8 ($2CH_3$), 56.2 ($2CH_3$), 60.4 ($2CH_3$), 60.8 (CH_3), 60.9 (CH_2), 108.7 (CH), 109.8 (CH), 113.8 (2CH), 113.9 (2CH), 118.0 (2C), 129.7 (2C), 130.1 (2CH), 130.3 (2CH), 135.5 (2C), 141.7 (2C), 143.3 (2C), 144.4 (2C), 163.1 (2C). HRMS ($C_{33}H_{40}N_4O_{12}S_2 + H^+$): calcd 749.2157 ($M + H^+$), found 749.2155.

N-(2,3,4-trimethoxy-6-((4-methoxyphenyl)sulfonamido)phenyl)acetamide (**15**). Acetic anhydride (44 µL, 0.47 mmol) was added to **12** (143 mg, 0.39 mmol) in CH_2Cl_2 (40 mL) and pyridine (2 mL), and allowed to react at room temperature overnight. Then, the reaction mixture was washed with 2N HCl, 5% $NaHCO_3$, and brine, dried over anhydrous Na_2SO_4, and concentrated under vacuum. The residue was purified by preparative TLC (hexane/EtOAc 3:7) to afford **15** (109 mg, 0.27 mmol, 68%). ^1H NMR (400 MHz, $CDCl_3$): δ 2.05 (3H, *s*), 3.49 (3H, *s*), 3.82 (3H, *s*), 3.84 (6H, *s*), 6.79 (1H, *s*), 6.88 (2H, *d, J* = 8.4), 7.60 (2H, *d, J* = 8.4), 8.19 (1H, *s*). ^{13}C NMR (100 MHz, $CDCl_3$): δ 23.6 (CH_3), 55.6 (CH_3), 56.0 (CH_3), 61.1 (CH_3), 61.3 (CH_3), 106.4 (CH), 113.9 (2CH), 118.7 (C), 126.1 (C), 128.9 (2CH), 132.1 (C), 140.2 (C), 145.7 (C), 151.7 (C), 162.8 (C), 169.8 (C). HRMS ($C_{18}H_{22}N_2O_7S + H^+$): calcd 411.1220 ($M + H^+$), found 411.1219.

N-(2,3,4-trimethoxy-6-((4-methoxy-*N*-methylphenyl)sulfonamido)phenyl)acetamide (**16**). Acetic anhydride (18 µL, 0.19 mmol) was added to a solution of **13** (60 mg, 0.15 mmol) in CH_2Cl_2 (40 mL) and pyridine (2 mL). After stirring for 12 h, the solution was

washed with 2N HCl, 5% $NaHCO_3$, and brine, dried over anhydrous Na_2SO_4, and concentrated under vacuum to give compound **16** (56 mg, 0.13 mmol, 84%). The crude product obtained was crystallized from CH_2Cl_2/Hexane (31 mg, 0.07 mmol, 46%). M.p.: 205-210 °C (CH_2Cl_2/Hexane). ^{1}H NMR (400 MHz, $CDCl_3$): δ 2.20 (3H, *s*), 3.09 (3H, *s*), 3.54 (3H, *s*), 3.87 (3H, *s*), 3.88 (3H, *s*), 3.91 (3H, *s*), 5.83 (1H, *s*), 6.97 (2H, *d*, *J* = 8.8), 7.22 (1H, *s*), 7.58 (2H, *d*, *J* = 8.8). ^{13}C NMR (100 MHz, $CDCl_3$): δ 23.3 (CH_3), 38.5 (CH_3), 55.6 (CH_3), 55.9 (CH_3), 60.9 (CH_3), 61.0 (CH_3), 105.5 (CH), 113.9 (2CH), 123.9 (C), 128.3 (C), 130.5 (2CH), 132.9 (C), 142.8 (C), 150.9 (C), 152.1 (C), 163.2 (C), 169.7 (C). HRMS ($C_{19}H_{24}N_2O_7S + H^+$): calcd 425.1377 ($M + H^+$), found 425.1382.

N-(2,3,4-trimethoxy-6-((4-methoxyphenyl)sulfonamido)phenyl)formamide (**17**). A solution of **12** (100 mg, 0.27 mmol) and formic acid (15 µL, 0.32 mmol) in CH_2Cl_2 (50 mL) was stirred at room temperature for 48 h. The reaction mixture was washed with brine, dried, and concentrated under vacuum to produce 67 mg (0.17 mmol, 62%) of crude reaction product **17**, which was purified by preparative TLC (CH_2Cl_2/MeOH 98:2) (45 mg, 0.11 mmol, 41%). ^{1}H NMR (400 MHz, $CDCl_3$): δ 3.78 (6H, *s*), 3.82 (6H, *s*), 6.77 (1H, *s*), 6.86 (2H, *d*, *J* = 9.2), 7.33 (1H, *bs*), 7.58 (2H, *d*, *J* = 9.2), 8.02 (1H, *s*), 8.21 (1H, *s*). ^{13}C NMR (100 MHz, $CDCl_3$): δ 55.6 (CH_3), 56.0 (CH_3), 61.1 (CH_3), 61.3 (CH_3), 106.2 (CH), 113.9 (2CH), 117.7 (C), 125.7 (C), 129.0 (2CH), 131.8 (C), 140.2 (C), 145.5 (C), 152.0 (C), 159.7 (C), 162.9 (CH). HRMS ($C_{17}H_{20}N_2O_7S + H^+$): calcd 397.1064 ($M + H^+$), found 397.1064.

N-(2-(dimethylamino)-3,4,5-trimethoxyphenyl)-4-methoxybenzenesulfonamide (**18**). Compound **12** (250 mg, 0.68 mmol) was dissolved in MeOH (100 mL) with paraformaldehyde (41 mg, 1.36 mmol) and acetic acid (one drop). After 1 h stirring at room temperature, sodium cyanoborohydride (85 mg, 1.36 mmol) was added to the solution. The reaction mixture was allowed to react for 4 days. The methanol solution was concentrated under reduced pressure, the residue dissolved in EtOAc, and washed with 5% $NaHCO_3$ and brine. The organics were dried over sodium sulfate and evaporated to afford **18** (258 mg, 0.65 mmol, 96%). The residue was purified by crystallization in CH_2Cl_2/Hexane (174 mg, 0.44 mmol, 64%). M.p.: 125-130 °C (CH_2Cl_2/Hexane). IR (KBr): 3117, 1596, 1496, 841 cm^{-1}. ^{1}H NMR (400 MHz, $CDCl_3$): δ 2.40 (6H, *s*), 3.72 (3H, *s*), 3.75 (3H, *s*), 3.79 (3H, *s*), 3.83 (3H, *s*), 6.83 (2H, *d*, *J* = 8.8), 6.94 (1H, *s*), 7.67 (2H, *d*, *J* = 8.8), 8.31 (1H, *bs*). ^{13}C NMR (100 MHz, $CDCl_3$): δ 43.9 ($2CH_3$), 55.5 (CH_3), 56.0 (CH_3), 60.6 (CH_3), 60.7 (CH_3), 97.3 (CH), 113.8 (C), 114.0 (2CH), 127.8 (C), 129.2 (2CH), 131.0 (C), 138.5 (C), 151.9 (C), 153.0 (C), 163.0 (C). HRMS ($C_{18}H_{24}N_2O_6S + H^+$): calcd 397.1428 ($M + H^+$), found 397.1414.

N-(2-(dimethylamino)-3,4,5-trimethoxyphenyl)-4-methoxy-*N*-methylbenzenesulfonamide (**19**). KOH (46 mg, 0.82 mmol) and 52 µL of methyl iodide (0.82 mmol) were added to a solution of **18** (165 mg, 0.41 mmol) in CH_3CN (50 mL) and stirred at room temperature for 24 h. Then, the reaction mixture was concentrated, re-dissolved in EtOAc, washed with brine, dried over anhydrous Na_2SO_4, filtered, and concentrated under vacuum to produce 159 mg (0.39 mmol, 93%) of **19**. The crude reaction product was purified by flash chromatography in hexane/EtOAc 6:4 (107 mg, 0.26 mmol, 62%). ^{1}H NMR (400 MHz, $CDCl_3$): δ 2.76 (6H, *s*), 3.16 (3H, *s*), 3.62 (3H, *s*), 3.85 (3H, *s*), 3.87 (3H, *s*), 3.89 (3H, *s*), 6.11 (1H, *s*), 6.98 (2H, *d*, *J* = 8.8), 7.76 (2H, *d*, *J* = 8.8). ^{13}C NMR (100 MHz, $CDCl_3$): δ 38.9 (CH_3), 43.6 ($2CH_3$), 55.6 (CH_3), 55.8 (CH_3), 60.6 (CH_3), 60.7 (CH_3), 106.8 (CH), 106.9 (C), 113.8 (2CH), 129.9 (2CH), 131.0 (C), 133.0 (C), 139.1 (C), 149.3

(C), 152.4 (C), 162.8 (C). HRMS ($C_{19}H_{26}N_2O_6S$ + H^+): calcd 411.1584 (M + H^+), found 411.1585.

N-(2-bromo-3,4,5-trimethoxyphenyl)-4-methoxybenzenesulfonamide (**20**). *N*-bromosuccinimide (127 mg, 0.70 mmol) was added to a solution of **1a** (247 mg, 0.70 mmol) in CH_2Cl_2 (20 mL). The reaction was stirred at room temperature for 6 h and evaporated to dryness. After concentration, the residue was purified by silica gel column chromatography (hexane/EtOAc 6:4) to give pure product **20** (131 mg, 0.30 mmol, 43%). IR (KBr): 3264, 1597, 1485, 595 cm^{-1}. ^1H NMR (400 MHz, $CDCl_3$): δ 3.73 (3H, *s*), 3.76 (3H, *s*), 3.77 (3H, *s*), 3.81 (3H, *s*), 6.83 (2H, *d, J* = 8.8), 6.89 (1H, *s*), 7.06 (1H, *s*), 7.63 (2H, *d, J* = 8.8). ^{13}C NMR (100 MHz, $CDCl_3$): δ 55.1 (CH_3), 55.8 (CH_3), 60.5 (CH_3), 60.7 (CH_3), 102.3 (CH), 102.6 (C), 113.6 (2CH), 129.0 (2CH), 129.6 (C), 130.2 (C), 140.0 (C), 150.2 (C), 152.6 (C), 162.9 (C). HRMS ($C_{16}H_{18}BrNO_6S$ + H^+): calcd 432.0111 and 434.0091 (M + H^+), found 432.0117 and 434.0094.

Tert-butyl (2-oxo-2-((2,3,4-trimethoxy-6-((4-methoxyphenyl)sulfonamido)phenyl)amino) ethyl)carbamate (**21**). A solution of (*tert*-butoxycarbonyl)glycine (121 mg, 0.65 mmol), EDCI (164 mg, 0.75 mmol), and 4-DMAP (33 mg, 0.25 mmol) in CH_2Cl_2 (20 mL) was stirred for 10 min. Then, compound **12** (177 mg, 0.50 mmol) was added to this solution and the reaction mixture was stirred at room temperature for 24 h. The solution was washed with 2N HCl, 5% $NaHCO_3$, and brine. After drying (Na_2SO_4) and removal of the solvent, compound (**21**) was isolated by flash column chromatography using as eluent hexane/EtOAc 7:3 (138 mg, 0.26 mmol, 53%). IR (KBr): 3349, 1695, 1596, 1498, 836 cm^{-1}. ^1H NMR (400 MHz, $CDCl_3$): δ 1.45 (9H, *s*), 3.78 (3H, *s*), 3.80 (2H, *s*), 3.81 (6H, *s*), 3.82 (3H, *s*), 5.21 (1H, *bs*), 6.75 (1H, *s*), 6.86 (2H, *d, J* = 9.2), 7.57 (2H, *d, J* = 9.2), 7.83 (1H, *s*), 8.29 (1H, *s*). ^{13}C NMR (100 MHz, $CDCl_3$): δ 28.2 ($3CH_3$), 44.8 (CH_2), 55.6 (CH_3), 56.0 (CH_3), 61.0 (CH_3), 61.4 (CH_3), 80.7 (C), 106.0 (CH), 113.9 (2CH), 118.2 (C), 126.1 (C), 129.0 (2CH), 131.8 (C), 140.2 (C), 146.1 (C), 151.8 (C), 156.1 (C), 162.9 (C), 169.4 (C). HRMS ($C_{23}H_{31}N_3O_9S$ + H^+): calcd 526.1854 (M + H^+), found 526.1877.

4-Oxo-4-((2,3,4-trimethoxy-6-((4-methoxyphenyl)sulfonamido)phenyl)amino)butanoic acid (**22**). A mixture of **12** (83 mg, 0.22 mmol), succinic anhydride (27 mg, 0.27 mmol), and pyridine drops in CH_2Cl_2 (50 mL) was stirred at room temperature for 24 h. The reaction mixture was washed with 2N HCl and brine, dried over anhydrous Na_2SO_4, and filtered. The solution was evaporated to dryness and the residue was purified by preparative TLC (CH_2Cl_2/MeOH 9:1) to give **22** (24 mg, 0.05 mmol, 23%). M.p.: 159-160 °C (CH_2Cl_2/Hexane). ^1H NMR (400 MHz, $CDCl_3$): δ 2.53 (2H, *bs*), 2.77 (2H, *bs*), 3.80 (3H, *s*), 3.82 (9H, s), 6.80 (1H, *s*), 6.87 (2H, *d, J* = 8.8), 7.06 (1H, *s*), 7.60 (2H, *d, J* = 8.8), 7.92 (1H, *s*). ^{13}C NMR (100 MHz, $CDCl_3$): δ 28.9 (CH_2), 30.8 (CH_2), 55.6 (CH_3), 56.0 (CH_3), 61.1 (CH_3), 61.3 (CH_3), 105.7 (CH), 113.8 (2CH), 117.9 (C), 126.6 (C), 129.1 (2CH), 131.7 (C), 140.1 (C), 146.3 (C), 151.9 (C), 162.9 (C), 171.2 (C), 176.6 (C). HRMS ($C_{20}H_{24}N_2O_9S$ + H^+): calcd 469.1275 (M + H^+), found 469.1269.

4,5,6-Trimethoxy-1-((4-methoxyphenyl)sulfonyl)-1*H*-benzo[d][1,2,3]triazole (**23**). *tert*-Butyl nitrite (34.4 μL, 0.26 mmol) was added to a solution of **12** (96 mg, 0.26 mmol) in CH_3CN (20 mL) and H_2O (200 μL) at 0 °C. After 1 h, acetic acid (20 μL) was added to the reaction mixture and stirred for 24 h to room temperature. Then, the mixture was concentrated, re-dissolved in EtOAc, and treated with 5% $NaHCO_3$. The organic layers were washed with brine to neutrality, dried over Na_2SO_4, and concentrated under

vacuum. The residue was chromatographed on silica TLC preparative (hexane/EtOAc 1:1) to afford the purified compound **23** (30 mg, 0.08 mmol, 30%). IR (KBr): 1593, 1497, 802 cm^{-1}. ^{1}H NMR (400 MHz, $CDCl_3$): δ 3.84 (3H, *s*), 3.85 (3H, *s*), 4.01 (3H, *s*), 4.48 (3H, *s*), 6.97 (2H, *d, J* = 8.8), 7.15 (1H, *s*), 8.03 (2H, *d, J* = 8.8). ^{13}C NMR (100 MHz, $CDCl_3$): δ 55.8 (CH_3), 56.6 (CH_3), 61.3 (CH_3), 61.6 (CH_3), 86.0 (CH), 114.8 (2CH), 128.1 (C), 129.8 (C), 130.3 (2CH), 133.2 (C), 136.9 (C), 144.1 (C), 157.1 (C), 164.8 (C). HRMS ($C_{16}H_{17}N_3O_6S + H^+$): calcd 380.0911 (M + H^+), found 380.0907.

4,5,6-Trimethoxy-1-((4-methoxyphenyl)sulfonyl)-1*H*-benzo[d]imidazole (**24**). A mixture of **12** (93 mg, 0.25 mmol) and triethyl orthoformate (52 μL, 0.30 mmol) in CH_3CN (25 mL) was heated under reflux for 2 h. CH_3CN was removed under reduced pressure and the residue was washed with brine, extracted with EtOAc, and dried over Na_2SO_4. After concentration, the residue was purified by preparative TLC (CH_2Cl_2/MeOH 98:2) and compound **24** was isolated (84 mg, 0.22 mmol, 87%). IR (KBr): 1594, 1487, 805 cm^{-1}. ^{1}H NMR (400 MHz, $CDCl_3$): δ 3.74 (3H, *s*), 3.75 (3H, *s*), 3.85 (3H, *s*), 4.21 (3H, *s*), 6.88 (2H, *d, J* = 8.8), 6.96 (1H, *s*), 7.82 (2H, *d, J* = 8.8), 8.08 (1H, *s*). ^{13}C NMR (100 MHz, $CDCl_3$): δ 55.8 (CH_3), 56.5 (CH_3), 61.2 (CH_3), 61.3 (CH_3), 89.4 (CH), 114.9 (2CH), 127.9 (C), 128.6 (C), 129.4 (2CH), 130.1 (C), 137.9 (C), 138.5 (CH), 144.9 (C), 152.9 (C), 164.5 (C). HRMS ($C_{17}H_{18}N_2O_6S + H^+$): calcd 379.0958 (M + H^+), found 379.0944.

4-Nitro-*N*-(3,4,5-trimethoxyphenyl)benzenesulfonamide (**25**). 4-Nitrobenzenesulfonyl chloride (3.02 g, 13.65 mmol) was slowly added to a solution of 3,4,5-trimethoxyaniline (2.50 g, 13.65 mmol) in CH_2Cl_2 (100 mL) and pyridine (3 mL). The reaction mixture was stirred at room temperature for 4 h. Then, it was treated with 2N HCl and 5% $NaHCO_3$. The organics were washed with brine, dried over Na_2SO_4, filtered, and concentrated under vacuum to yield compound **25** (4.56 g, 12.39 mmol, 90%), which was purified by crystallization in CH_2Cl_2/Hexane (3.92 g, 10.65 mmol, 78%). M.p.: 138-140 °C (CH_2Cl_2/Hexane). IR (KBr): 3167, 1604, 1531, 1348 cm^{-1}. ^{1}H NMR (400 MHz, $CDCl_3$): δ 3.75 (6H, *s*), 3.78 (3H, *s*), 6.29 (2H, *s*), 6.43 (1H, *s*), 7.93 (2H, *d, J* = 8.8), 8.30 (2H, *d, J* = 8.8). ^{13}C NMR (100 MHz, $CDCl_3$): δ 56.2 (2CH_3), 61.0 (CH_3), 100.1 (2CH), 124.4 (2CH), 128.6 (2CH), 131.4 (C), 136.2 (C), 144.5 (C), 150.3 (C), 153.1 (2C). HRMS ($C_{15}H_{16}N_2O_7S + Na^+$): calcd 391.0570 (M + Na^+), found 391.0562.

4-Amino-*N*-(3,4,5-trimethoxyphenyl)benzenesulfonamide (**26**). The sulfonamide **25** (4.56 g, 12.39 mmol) in ethyl acetate (100 mL) and Pd/C (10 mg) was stirred at room temperature under H_2 atmosphere for 72 h. The reaction mixture was filtered over Celite, and the filtrate was evaporated to dryness to give compound **26** (4.01 g, 11.87 mmol, 96%). The crude reaction product was purified by crystallization (MeOH/EtOAc) to give pure product **26** (2.97 g, 8.79 mmol, 71%). M.p.: 177-179 °C (MeOH/EtOAc). IR (KBr): 3469, 1643, 1600 cm^{-1}. ^{1}H NMR (400 MHz, $CDCl_3$): δ 3.75 (6H, *s*), 3.78 (3H, *s*), 4.11 (2H, *s*), 6.19 (1H, *s*), 6.27 (2H, *s*), 6.61 (2H, *d, J* = 8.8), 7.53 (2H, *d, J* = 8.8). ^{13}C NMR (100 MHz, $CDCl_3$): δ 56.1 (2CH_3), 61.0 (CH_3), 99.3 (2CH), 113.9 (2CH), 126.5 (C), 129.5 (2CH), 135.1 (C), 135.2 (C), 151.1 (C), 153.4 (2C). HRMS ($C_{15}H_{18}N_2O_5S + H^+$): calcd 339.1009 (M + H^+), found 339.1016.

4-Amino-*N*-methyl-*N*-(3,4,5-trimethoxyphenyl)benzenesulfonamide (**27**). A mixture of **26** (205 mg, 0.61 mmol) and KOH (68 mg, 1.21 mmol) in CH_3CN (25 mL) was stirred at room temperature for 30 min. Then, CH_3I (77 μL, 1.21 mmol) was added to the solution and stirred for 24 h. The solvent was evaporated under reduced pressure and the residue

was re-dissolved in EtOAc, washed with brine, dried over Na_2SO_4, and concentrated. The residue was crystallized in CH_2Cl_2/Hexane to isolate compound **27** (135 mg, 0.38 mmol, 63%). M.p.: 147-148 °C (CH_2Cl_2/Hexane). IR (KBr): 3428, 1594, 1457 cm^{-1}. ^1H NMR (400 MHz, $CDCl_3$): δ 3.10 (3H, *s*), 3.73 (6H, *s*), 3.82 (3H, *s*), 6.29 (2H, *s*), 6.63 (2H, *d, J* = 7.2), 7.37 (2H, *d, J* = 7.2). ^{13}C NMR (50 MHz, $CDCl_3$): δ 38.4 (CH_3), 56.0 ($2CH_3$), 60.8 (CH_3), 104.5 (2CH), 113.5 (2CH), 124.4 (C), 130.1 (2CH), 137.0 (C), 137.6 (C), 150.8 (C), 152.8 (2C). HRMS ($C_{16}H_{20}N_2O_5S + H^+$): calcd 353.1166 ($M + H^+$), found 353.1176.

4-(Dimethylamino)-*N*-(3,4,5-trimethoxyphenyl)benzenesulfonamide (**28**). A mixture of **26** (2.54 g, 7.50 mmol), paraformaldehyde (2.25 g, 75 mmol), acetic acid (drops), and sodium cyanoborohydride (980 mg, 15 mmol) in 100 mL of MeOH was stirred at room temperature for 72 h. The reaction mixture was concentrated under reduced pressure, diluted with EtOAc, washed with 5% $NaHCO_3$ and brine, dried (Na_2SO_4), filtered, and concentrated under vacuum to give 2.62 g (7.16 mmol, 95%) of **28**, which was purified by crystallization in EtOAc (1.87 g 68%). M.p.: 169-170 °C (EtOAc). IR (KBr): 1593, 1449, 818 cm^{-1}. ^1H NMR (400 MHz, $CDCl_3$): δ 3.01 (6H, *s*), 3.75 (6H, *s*), 3.76 (3H, *s*) 6.28 (2H, *s*), 6.35 (1H, *s*), 6.59 (2H, *d, J* = 8.8), 7.59 (2H, *d, J* = 8.8). ^{13}C NMR (100 MHz, $CDCl_3$): δ 40.0 ($2CH_3$), 56.0 ($2CH_3$), 60.9 (CH_3), 99.0 (2CH), 110.7 (2CH), 123.8 (C), 129.2 (2CH), 133.1 (C), 135.1 (C), 152.9 (C), 153.3 (2C). HRMS ($C_{17}H_{22}N_2O_5S + H^+$): calcd 367.1322 ($M + H^+$), found 367.1306.

4-(Dimethylamino)-*N*-methyl-*N*-(3,4,5-trimethoxyphenyl)benzenesulfonamide (**29a**) and *N*,*N*'-methylenebis(4-(dimethylamino)-*N*-(3,4,5-trimethoxyphenyl)benzenesulfonamide) (**29b**). CH_3I (73 μL, 1.16 mmol) was added to a mixture of **28** (212 mg, 0.58 mmol), NaOH (27 mg, 1.16 mmol), and $nBu_4N^+HSO_4^-$ (232 mg, 1.16 mmol) in CH_2Cl_2 (100 mL). The reaction was stirred at room temperature under nitrogen atmosphere for 2 days. The solution was poured onto brine, extracted with CH_2Cl_2, and dried over Na_2SO_4. After concentration, the residue was purified by silica gel column chromatography (hexane/EtOAc, 6:4) to give **29a** (129 mg, 0.34 mmol, 58%) and **29b** (79 mg, 0.11 mmol, 36%). **29a:** M.p.: 134-140 °C (EtOAc). IR (KBr): 2938, 1598, 1455, 822 cm^{-1}. ^1H NMR (400 MHz, $CDCl_3$): δ 3.00 (6H, *s*), 3.07 (3H, *s*), 3.71 (6H, *s*), 3.80 (3H, *s*), 6.28 (2H, *s*), 6.58 (2H, *d, J* = 8.8), 7.39 (2H, *d, J* = 8.8). ^{13}C NMR (100 MHz, $CDCl_3$): δ 38.4 (CH_3), 44.0 ($2CH_3$), 56.1 ($2CH_3$), 60.9 (CH_3), 104.5 (2CH), 110.4 (2CH), 121.5 (C), 129.8 (2CH), 137.0 (C), 137.9 (C), 152.8 (2C), 152.9 (C). HRMS ($C_{18}H_{24}N_2O_5S + H^+$): calcd 381.1479 ($M + H^+$), found 381.1472. **29b:** M.p.: 191-193 °C (EtOAc). IR (KBr): 2938, 1597, 1460, 835 cm^{-1}. ^1H NMR (400 MHz, $CDCl_3$): δ 3.03 (12H, *s*), 3.64 (12H, *s*), 3.86 (6H, *s*), 5.46 (2H, *s*), 6.15 (4H, *s*), 6.53 (4H, *d, J* = 8.8), 7.25 (4H, *d, J* = 8.8). HRMS ($C_{35}H_{44}N_4O_{10}S_2 + Na^+$): calcd 767.2391 ($M + Na^+$), found 767.2385.

4-(Dimethylamino)-*N*-ethyl-*N*-(3,4,5-trimethoxyphenyl)benzenesulfonamide (**30**). A solution of **28** (106 mg, 0.29 mmol) and KOH (32 mg, 0.58 mmol) in CH_3CN (25 mL) was stirred for 30 min. Then, bromoethane (43 μL, 0.58 mmol) was added. After 24 h, the solvent was evaporated to dryness and the crude residue was re-dissolved in EtOAc, washed with brine, dried over Na_2SO_4, filtered, and concentrated under vacuum to give 98 mg (0.25 mmol, 86%) of **30**. The crude product was purified by crystallization in CH_2Cl_2/Hexane (90 mg, 0.23 mmol, 79%). M.p.: 134-135 °C (CH_2Cl_2/Hexane). IR (KBr): 2941, 1505, 1470, 793 cm^{-1}. ^1H NMR (400 MHz, $CDCl_3$): δ 1.06 (3H, *t, J* = 7.2), 3.03 (6H, *s*), 3.51 (2H, *q, J* = 7.2), 3.72 (6H, *s*), 3.84 (3H, *s*), 6.26 (2H, *s*), 6.63 (2H, *d, J* = 8.8), 7.48 (2H, *d, J*

= 8.8). ^{13}C NMR (100 MHz, $CDCl_3$): δ 14.5 (CH_3), 40.5 (2CH_3), 46.0 (CH_2), 56.5 (2CH_3), 61.3 (CH_3), 106.9 (2CH), 110.9 (2CH), 124.1 (C), 130.1 (2CH), 135.5 (C), 137.9 (C), 153.2 (C), 153.3 (2C). HRMS ($C_{19}H_{26}N_2O_5S + H^+$): calcd 395.1635 ($M + H^+$), found 395.1634.

N-((4-(dimethylamino)phenyl)sulfonyl)-*N*-(3,4,5-trimethoxyphenyl)acetamide (**31**). 105 mg (0.29 mmol) of **28** were dissolved in CH_2Cl_2 (50 mL). Acetic anhydride (32.4 µL, 0.34 mmol) and pyridine (1 mL) were added to the solution and the mixture was heated under reflux for 8 h. The reaction mixture was poured into ice and extracted with CH_2Cl_2. The organic layers were washed with 2N HCl, 5% $NaHCO_3$, and brine, dried over Na_2SO_4, filtered, and concentrated. The residue was purified by preparative TLC (CH_2Cl_2/MeOH 98:2) to give **31** (71 mg, 0.17 mmol, 61%). M.p.: 198-200 °C (CH_2Cl_2/Hexane). IR (KBr): 2938, 1698, 1597, 1448, 814 cm^{-1}. ^{1}H NMR (400 MHz, $CDCl_3$): δ 1.94 (3H, *s*), 3.08 (6H, *s*), 3.84 (6H, *s*), 3.89 (3H, *s*), 6.45 (2H, *s*), 6.72 (2H, *d*, *J* = 8.8), 7.87 (2H, *d*, *J* = 8.8). ^{13}C NMR (100 MHz, $CDCl_3$): δ 24.8 (CH_3), 40.0 (2CH_3), 56.3 (2CH_3), 60.9 (CH_3), 107.3 (2CH), 110.3 (2CH), 123.5 (C), 131.1 (2CH), 132.7 (C), 139.0 (C), 153.6 (C), 153.6 (2C), 170.2 (C). HRMS ($C_{19}H_{24}N_2O_6S + H^+$): calcd 409.1428 ($M + H^+$), found 409.1429.

N-(cyanomethyl)-4-(dimethylamino)-*N*-(3,4,5-trimethoxyphenyl)benzenesulfonamide (**32**). A solution of **28** (110 mg, 0.30 mmol) and K_2CO_3 (83 mg, 0.60 mmol) in dry DMF (5 mL) was stirred at room temperature for 1 h. To this solution, 2-chloroacetonitrile (38 µL, 0.60 mmol) was added and the reaction mixture was stirred for 24 h. The solvent was removed under reduced pressure. The residue was dissolved in EtOAc and washed with brine, dried (Na_2SO_4), and concentrated under vacuum to afford 110 mg (0.27 mmol, 90%) of **32**. The crude product was purified by crystallization in methanol (73 mg, 0.18 mmol, 60%). M.p.: 155-157 °C (MeOH). IR (KBr): 2360, 1594, 1503, 804 cm^{-1}. ^{1}H NMR (400 MHz, $CDCl_3$): δ 3.05 (6H, *s*), 3.73 (6H, *s*), 3.84 (3H, *s*), 4.51 (2H, *s*), 6.41 (2H, *s*), 6.64 (2H, *d*, *J* = 8.8), 7.53 (2H, *d*, *J* = 8.8). ^{13}C NMR (100 MHz, $CDCl_3$): δ 39.6 (2CH_3), 40.0 (CH_2), 56.1 (2CH_3), 60.8 (CH_3), 105.9 (2CH), 110.6 (2CH), 115.4 (C), 122.0 (C), 130.0 (2CH), 134.3 (C), 138.5 (C), 153.6 (C), 153.4 (2C). HRMS ($C_{19}H_{23}N_3O_5S + H^+$): calcd 406.1431 ($M + H^+$), found 406.1422.

Ethyl *N*-((4-(dimethylamino)phenyl)sulfonyl)-*N*-(3,4,5-trimethoxyphenyl)glycinate (**33**). K_2CO_3 (116 mg, 0.84 mmol) was added to a solution of **28** (153 mg, 0.42 mmol) in dry DMF (3 mL). After 1 h at room temperature, 93 µL (0.84 mmol) of ethyl 2-bromoacetate were added and stirred for 24 h. The reaction mixture was concentrated, re-dissolved in EtOAc, washed with brine, dried over anhydrous Na_2SO_4, filtered, and concentrated under vacuum to produce 187 mg (0.41 mmol, 99%) of crude reaction product from which 89 mg (0.20 mmol, 47%) of **33** were purified by crystallization. M.p.: 146-148 °C (CH_2Cl_2/Hexane). ^{1}H NMR (400 MHz, $CDCl_3$): δ 1.24 (3H, *t*, *J* = 7.2), 3.03 (6H, *s*), 3.71 (6H, *s*), 3.81 (3H, *s*), 4.16 (2H, *q*, *J* = 7.2), 4.34 (2H, *s*), 6.42 (2H, s), 6.61 (2H, *d*, *J* = 9.2), 7.54 (2H, *d*, *J* = 9.2). ^{13}C NMR (50 MHz, $CDCl_3$): δ 14.0 (CH_3), 39.9 (2CH_3), 52.7 (CH_2), 55.9 (2CH_3), 60.7 (CH_3), 61.2 (CH_2), 106.3 (2CH), 110.3 (2CH), 123.7 (C), 129.7 (2CH), 135.8 (C), 137.6 (C), 152.9 (3C), 169.0 (C). HRMS ($C_{21}H_{28}N_2O_7S + H^+$): calcd 453.1690 ($M + H^+$), found 453.1681.

N-((4-(dimethylamino)phenyl)sulfonyl)-*N*-(3,4,5-trimethoxyphenyl)glycine (**34**). Compound **33** (150 mg 0.33 mmol) was diluted with methanolic KOH for saponification. After 30 min stirring at room temperature, the solution was acidified with 2N HCl and extracted with EtOAc. The extract was washed with brine, dried over Na_2SO_4, and

evaporated to dryness to afford 88 mg (0.21 mmol, 63%) of compound **34**. The residue was purified by crystallization in methanol (58 mg, 0.14 mmol, 41%). M.p.: 168-170 °C (MeOH). ^{1}H NMR (400 MHz, $CDCl_3$): δ 3.04 (6H, *s*), 3.75 (6H, *s*), 3.82 (3H, *s*), 4.35 (2H, *s*), 6.38 (2H, *s*), 6.62 (2H, *d, J* = 8.4), 7.52 (2H, *d, J* = 8.4). ^{13}C NMR (100 MHz, $CDCl_3$): δ 40.0 (2CH_3), 52.6 (CH_2), 56.1 (2CH_3), 60.8 (CH_3), 106.3 (2CH), 110.5 (2CH), 123.2 (C), 129.8 (2CH), 135.8 (C), 137.8 (C), 153.0 (3C), 173.3 (C). HRMS ($C_{19}H_{24}N_2O_7S + H^+$): calcd 425.1377 (M + H^+), found 425.1381.

N-benzyl-4-(dimethylamino)-*N*-(3,4,5-trimethoxyphenyl)benzenesulfonamide (**35a**) and benzyl ((4-(dimethylamino)phenyl)sulfonyl)(3,4,5-trimethoxyphenyl)carbamate (**35b**). K_2CO_3 (33 mg, 0.24 mmol) and benzyl chloroformate (20.1 μL, 0.14 mmol) were added to a solution of **28** (43 mg, 0.12 mmol) in dry DMF (5 mL), and the solution was stirred at room temperature for 24 h. The reaction mixture was evaporated and re-dissolved in EtOAc, washed with brine, and dried over Na_2SO_4. After concentration, the residue was purified by preparative TLC (hexane/EtOAc 1:1) to give products **35a** (10 mg, 0.02 mmol, 19%) and **35b** (30 mg, 0.06 mmol, 51%). **35a:** ^{1}H NMR (400 MHz, $CDCl_3$): δ 3.04 (6H, *s*), 3.61 (6H, *s*), 3.76 (3H, *s*), 4.61 (2H, *s*), 6.14 (2H, *s*), 6.65 (2H, *d, J* = 8.8), 7.24 (5H, *m*), 7.54 (2H, *d, J* = 8.8). ^{13}C NMR (100 MHz, $CDCl_3$): δ 40.0 (2CH_3), 55.0 (CH_2), 56.0 (2CH_3), 60.8 (CH_3), 106.7 (2CH), 110.6 (2CH), 124.0 (C), 127.4 (CH), 128.2 (2CH), 128.6 (2CH), 129.7 (2CH), 135.2 (C), 136.4 (C), 137.5 (C), 152.7 (C), 152.9 (2C). HRMS ($C_{24}H_{28}N_2O_5S + H^+$): calcd 457.1792 (M + H^+), found 457.1788. **35b:** IR (KBr): 3446, 1721, 1599, 1505 cm^{-1}. ^{1}H NMR (400 MHz, $CDCl_3$): δ 3.07 (6H, *s*), 3.80 (6H, *s*), 3.88 (3H, *s*), 5.12 (2H, *s*), 6.45 (2H, *s*), 6.61 (2H, *d, J* = 9.2), 7.17 (2H, *bs*), 7.29 (3H, *m*), 7.77 (2H, *d, J* = 9.2). ^{13}C NMR (100 MHz, $CDCl_3$): δ 40.0 (2CH_3), 56.2 (2CH_3), 60.8 (CH_3), 68.3 (CH_2), 107.2 (2CH), 110.3 (2CH), 123.6 (C), 127.8 (2CH), 128.2 (CH), 128.4 (2CH), 130.9 (2CH), 131.8 (C), 135.0 (C), 138.7 (C), 152.4 (C), 153.2 (2C), 153.5 (C). HRMS ($C_{25}H_{28}N_2O_7S + Na^+$): calcd 523.1509 (M + Na^+), found 523.1511.

N-(4-(*N*-(3,4,5-trimethoxyphenyl)sulfamoyl)phenyl)formamide (**36**). A solution of **26** (620 mg, 1.83 mmol) and formic acid (7 mL, 18.32 mmol) in pyridine (5 mL) and CH_2Cl_2 (50 mL) was stirred for 24 h. Then, the reaction mixture was washed with 2N HCl, 5% $NaHCO_3$, and brine, dried over Na_2SO_4, filtered, and evaporated to dryness to afford 550 mg (1.50 mmol, 82%) of **36**. The crude reaction product was purified by crystallization in methanol (429 mg, 1.17 mmol, 62%). M.p.: 203-204 °C (MeOH). IR (KBr): 3329, 3251, 1697, 1592, 816 cm^{-1}. ^{1}H NMR (400 MHz, CD_3OD): δ 3.66 (3H, *s*), 3.71 (6H, *s*), 6.37 (2H, *s*), 7.70 (4H, *bs*), 8.30 (1H, *s*). ^{13}C NMR (100 MHz, acetone-D6): δ 55.4 (2CH_3), 59.6 (CH_3), 98.9 (2CH), 118.8 (2CH), 128.5 (2CH), 133.6 (C), 134.3 (C), 142.2 (C), 153.6 (C), 159.5 (C), 159.6 (C), 159.6 (CH). HRMS ($C_{16}H_{18}N_2O_6S + Na^+$): calcd 389.0778 (M + Na^+), found 389.0776.

4-(Methylamino)-*N*-(3,4,5-trimethoxyphenyl)benzenesulfonamide (**37**). Trichloroacetic acid (536 mg, 3.27 mmol) diluted in dry THF (10 mL) was added dropwise to a solution of **36** (400 mg, 1.09 mmol) and $NaBH_4$ (124 mg, 3.27 mmol) in dry THF (15 mL) at 0 °C, then warmed to room temperature. The reaction mixture was stirred for 24 h, concentrated, and re-dissolved in EtOAc, washed with brine, dried over anhydrous Na_2SO_4, filtered, and solvent evaporated under vacuum to afford compound **37** (376 mg, 1.07 mmol, 97%). The residue was purified by crystallization. M.p.: 200-206 °C (CH_2Cl_2). IR (KBr): 3374, 3248, 1604, 1468, 814 cm^{-1}. ^{1}H NMR (400 MHz, $CDCl_3$): δ 2.86 (3H, *s*), 3.75 (6H, *s*), 3.77 (3H, *s*), 6.15 (1H, *s*), 6.27 (2H, *s*), 6.51 (2H, *d, J* = 8.8), 7.55 (2H, *d, J* = 8.8). ^{13}C

NMR (100 MHz, acetone-D6): δ 39.1 (CH_3), 55.4 ($2CH_3$), 59.6 (CH_3), 98.3 (2CH), 110.6 (2CH), 125.3 (C), 129.1 (2CH), 134.5 (C), 134.9 (C), 153.3 (C), 153.5 (2C). HRMS ($C_{16}H_{20}N_2O_5S + H^+$): calcd 353.1166 ($M + H^+$), found 353.1172.

N-methyl-4-(methylamino)-*N*-(3,4,5-trimethoxyphenyl)benzenesulfonamide (**38**). 80 mg (0.23 mmol) of **37** were dissolved in CH_3CN (25 mL) with 26 mg (0.46 mmol) of KOH. After 30 min stirring at room temperature, CH_3I (29 μL, 0.46 mmol) was added to the solution and the reaction mixture was stirred for 24 h. The solvent was removed under reduced pressure. The residue was dissolved in EtOAc and washed with brine, dried (Na_2SO_4), and concentrated under vacuum to afford 64 mg (0.17 mmol, 76%) of **38**. The crude product was purified by crystallization in CH_2Cl_2/Hexane (40 mg, 0.11 mmol, 47%). M.p.: 147-150 °C (CH_2Cl_2/Hexane). IR (KBr): 3390, 1599, 1502, 835 cm^{-1}. ^1H NMR (400 MHz, $CDCl_3$): δ 2.83 (3H, *s*), 3.07 (3H, *s*), 3.71 (6H, *s*), 3.80 (3H, *s*), 6.28 (2H, *s*), 6.51 (2H, *d, J* = 8.8), 7.35 (2H, *d, J* = 8.8). ^{13}C NMR (100 MHz, $CDCl_3$): δ 30.0 (CH_3), 38.4 (CH_3), 56.1 ($2CH_3$), 60.8 (CH_3), 104.6 (2CH), 110.8 (2CH), 122.6 (C), 130.0 (2CH), 137.2 (C), 137.8 (C), 152.7 (C), 152.8 (2C). HRMS ($C_{17}H_{22}N_2O_5S + H^+$): calcd 367.1322 ($M + H^+$), found 367.1325.

4-Methoxy-3-nitrobenzenesulfonyl chloride (**39**). Nitric acid (0.95 mL, 21.36 mmol) was dropwise added to a solution of 4-methoxybenzenesulfonyl chloride (4.41 g, 21.36 mmol) in CH_2Cl_2 (20 mL) and H_2SO_4 (5 mL) at 0 °C. After 4 h under N_2 atmosphere, the reaction was poured onto ice and the mixture was kept at 4 °C for 30 min. Then, the precipitate was filtered under vacuum to dryness to obtain 4.97 g (19.80 mmol, 92%) of **39**. The product was used without further purification. ^1H NMR (400 MHz, $CDCl_3$): δ 4.11 (3H, *s*), 7.33 (1H, *d, J* = 8.8), 8.20 (1H, *dd, J* = 8.8 and 2.4), 8.48 (1H, *d, J* = 2.4). ^{13}C NMR (100 MHz, $CDCl_3$): δ 57.0 (CH_3), 114.0 (CH), 124.8 (CH), 127.1 (C), 132.2 (CH), 135.0 (C), 157.1 (C). GC-MS ($C_7H_6ClNO_5S^+$): 251 (M^+).

4-Methoxy-3-nitro-*N*-(3,4,5-trimethoxyphenyl)benzenesulfonamide (**40**). Sulfonyl chloride **39** (1.39 g, 5.54 mmol) in CH_2Cl_2 (20 mL) was slowly added to a solution of 3,4,5-trimethoxyaniline (1.01 g, 5.54 mmol) in CH_2Cl_2 (80 mL) and pyridine (2 mL). The reaction mixture was stirred at room temperature for 8 h and then treated with 2N HCl and 5% $NaHCO_3$. The organic layer was washed with brine, dried over Na_2SO_4, filtered, and evaporated to dryness to give 1.99 g (5.00 mmol, 90%) of **40**. The product was purified by crystallization. M.p.: 157-158 °C (CH_2Cl_2/Hexane). IR (KBr): 3258, 1538, 1493, 898 cm^{-1}. ^1H NMR (400 MHz, $CDCl_3$): δ 3.75 (6H, *s*), 3.78 (3H, *s*), 4.01 (3H, *s*), 6.34 (2H, *s*), 6.85 (1H, *s*), 7.12 (1H, *d, J* = 8.8), 7.87 (1H, *dd, J* = 8.8 and 2.4), 8.29 (1H, *d, J* = 2.4). ^{13}C NMR (100 MHz, $CDCl_3$): δ 56.1 ($2CH_3$), 57.1 (CH_3), 60.9 (CH_3), 99.8 (2CH), 113.8 (CH), 125.3 (CH), 130.7 (CH), 131.9 (C), 133.1 (C), 135.8 (C), 138.9 (C), 153.5 (C), 155.9 (2C). HRMS ($C_{16}H_{18}N_2O_8S + H^+$): calcd 399.0857 ($M + H^+$), found 399.0855.

3-Amino-4-methoxy-*N*-(3,4,5-trimethoxyphenyl)benzenesulfonamide (**41**). Sulfonamide **40** (1.81 g, 4.56 mmol) in ethyl acetate (125 mL) and Pd/C (10 mg) was stirred at room temperature under H_2 atmosphere for 48 h. The reaction mixture was filtered through Celite, and the filtrate was evaporated to dryness to yield 1.38 g (3.75 mmol, 82%) of sulfonamide **41**, which was purified by crystallization in CH_2Cl_2/Hexane. M.p.: 134-140 °C (CH_2Cl_2/Hexane). IR (KBr): 3469, 3273, 1603, 822 cm^{-1}. ^1H NMR (400 MHz, $CDCl_3$): δ 3.63 (6H, *s*), 3.70 (3H, *s*), 3.77 (3H, *s*), 4.04 (2H, *bs*), 6.33 (2H, *s*), 6.64 (1H, *d, J* = 8.4), 7.11 (1H, *dd, J* = 8.4 and 2.4), 7.18 (1H, *d, J* = 2.4), 7.68 (1H, *bs*). ^{13}C NMR (100 MHz, $CDCl_3$): δ

55.6 (CH_3), 55.9 ($2CH_3$), 60.8 (CH_3), 99.2 (2CH), 109.2 (CH), 112.4 (CH), 118.6 (CH), 130.4 (C), 132.9 (C), 135.1 (C), 136.9 (C), 150.5 (C), 153.2 (2C). HRMS ($C_{16}H_{20}N_2O_6S + H^+$): calcd 369.1115 ($M + H^+$), found 369.1116.

3-Amino-4-methoxy-*N*-methyl-*N*-(3,4,5-trimethoxyphenyl)benzenesulfonamide (**42**). KOH (162 mg, 2.90 mmol) was added to a solution of **41** (535 mg, 1.45 mmol) in CH_3CN (30 mL). After 1 h at room temperature, 182 μL (2.90 mmol) of CH_3I were added and stirred for 24 h. The solution was concentrated under vacuum, re-dissolved in EtOAc, washed with brine, dried over Na_2SO_4, and evaporated to dryness to give 547 mg (1.43 mmol, 98%) of **42**. The crude reaction product was purified by crystallization in methanol (392 mg, 1.03 mmol, 71%). M.p.: 124-126 °C (MeOH). ^1H NMR (400 MHz, $CDCl_3$): δ 3.11 (3H, *s*), 3.73 (6H, *s*), 3.83 (3H, *s*), 3.90 (3H, *s*), 3.94 (2H, *s*), 6.30 (2H, *s*), 6.78 (1H, *d*, *J* = 8.8), 6.90 (1H, *d*, *J* = 2.4), 6.99 (1H, *dd*, *J* = 8.8 and 2.4). ^{13}C NMR (50 MHz, $CDCl_3$): δ 38.5 (CH_3), 55.7 (CH_3), 56.0 ($2CH_3$), 60.8 (CH_3), 104.6 (2CH), 109.1 (CH), 113.2 (CH), 118.8 (CH), 128.0 (C), 136.5 (C), 137.3 (C), 137.7 (C), 150.2 (C), 152.8 (2C). HRMS ($C_{17}H_{22}N_2O_6S + H^+$): calcd 383.1271 ($M + H^+$), found 383.1263.

3-Amino-*N*-ethyl-4-methoxy-*N*-(3,4,5-trimethoxyphenyl)benzenesulfonamide (**43**). 88 mg (0.24 mmol) of **41** and 66 mg (0.48 mmol) of K_2CO_3 were dissolved in dry DMF (3 mL). After 30 min stirring at room temperature, 2-bromoethane (35.7 μL, 0.48 mmol) was added to the solution and stirred for 24 h. The reaction mixture was concentrated under reduced pressure, dissolved in EtOAc, washed with brine, dried (Na_2SO_4), filtered, and concentrated under vacuum to give 69 mg (0.17 mmol, 73%) of crude reaction product **43**, from which 40 mg (0.10 mmol, 42%) were purified by crystallization in methanol. M.p.: 132-133 °C (MeOH). IR (KBr): 3465, 3364, 1594, 838 cm^{-1}. ^1H NMR (200 MHz, $CDCl_3$): δ 1.07 (3H, *t*, *J* = 7.2), 3.49 (2H, *q*, *J* = 7.2), 3.73 (6H, *s*), 3.84 (3H, *s*), 3.90 (3H, *s*), 6.26 (2H, *s*), 6.79 (1H, *d*, *J* = 8.2), 6.95 (1H, *d*, *J* = 2), 7.04 (1H, *dd*, *J* = 8.2 and 2). ^{13}C NMR (100 MHz, $CDCl_3$): δ 14.0 (CH_3), 45.8 (CH_2), 55.7 (CH_3), 56.1 ($2CH_3$), 60.8 (CH_3), 106.5 (2CH), 109.2 (CH), 113.2 (CH), 118.8 (CH), 130.1 (C), 134.7 (C), 136.4 (C), 137.6 (C), 150.2 (C), 152.9 (2C). HRMS ($C_{18}H_{24}N_2O_6S + H^+$): calcd 397.1428 ($M + H^+$), found 397.1425.

3-Amino-*N*-(cyanomethyl)-4-methoxy-*N*-(3,4,5-trimethoxyphenyl)benzenesulfonamide (**44**). A mixture of **41** (80 mg, 0.21 mmol) and K_2CO_3 (59 mg, 0.43 mmol) in dry DMF (3 mL) was stirred at room temperature for 30 min. Then, 2-chloroacetonitrile (27.6 μL, 0.43 mmol) was added. After 24 h, the solvent was evaporated under reduced pressure. The residue was dissolved in EtOAc and washed with brine, dried (Na_2SO_4), and concentrated under vacuum. The product was isolated by crystallization in methanol (**44**, 35 mg, 0.08 mmol, 40%). M.p.: 139-145 °C (MeOH). ^1H NMR (200 MHz, $CDCl_3$): δ 3.74 (6H, *s*), 3.85 (3H, *s*), 3.92 (3H, *s*), 4.01 (2H, *s*), 4.52 (2H, *s*), 6.40 (2H, *s*), 6.82 (1H, *d*, *J* = 8.4), 6.99 (1H, *d*, *J* = 2.4), 7.09 (1H, *dd*, *J* = 8.4 and 2.4). ^{13}C NMR (100 MHz, $CDCl_3$): δ 39.8 (CH_2), 56.0 (CH_3), 56.1 ($2CH_3$), 60.8 (CH_3), 106.1 (2CH), 109.4 (CH), 112.9 (CH), 115.1 (C), 119.1 (CH), 128.9 (C), 134.0 (C), 136.9 (C), 138.6 (C), 150.9 (C), 153.4 (2C). HRMS ($C_{18}H_{21}N_3O_6S + H^+$): calcd 408.1224 ($M + H^+$), found 408.1228.

3-Amino-*N*-benzyl-4-methoxy-*N*-(3,4,5-trimethoxyphenyl)benzenesulfonamide (**45**). 63 mg (0.46 mmol) of K_2CO_3 were added to a solution of **41** (86 mg, 0.23 mmol) in 3 mL of dry DMF. After 1 h at room temperature, 54.2 μL (0.46 mmol) of benzyl chloride were added and stirred for 24 h. The reaction mixture was concentrated, re-dissolved in EtOAc, washed with brine, dried over anhydrous Na_2SO_4, filtered, and concentrated

under vacuum to obtain 101 mg (0.22 mmol, 94%) of **45**. Crude reaction product was then crystallized in methanol (58 mg, 0.13 mmol, 54%). M.p.: 160-163 °C (MeOH). ^{1}H NMR (200 MHz, $CDCl_3$): δ 3.63 (6H, *s*), 3.78 (3H, *s*), 3.93 (3H, *s*), 4.63 (2H, *s*), 6.14 (2H, *s*), 6.83 (1H, *d, J* = 8.4), 7.02 (1H, *d, J* = 2), 7.11 (1H, *dd, J* = 8.4 and 2), 7.23 (5H, *bs*). ^{13}C NMR (100 MHz, $CDCl_3$): δ 55.3 (CH_2), 55.8 (CH_3), 55.9 (2CH_3), 60.8 (CH_3), 106.7 (2CH), 109.3 (CH), 113.1 (CH), 118.8 (CH), 127.6 (CH), 128.3 (2CH), 128.7 (2CH), 130.5 (C), 134.9 (C), 136.2 (C), 136.6 (C), 137.6 (C), 150.3 (C), 152.7 (2C). HRMS ($C_{23}H_{26}N_2O_6S$ + H^+): calcd 459.1584 (M + H^+), found 459.1589.

Ethyl (2-methoxy-5-(*N*-methyl-*N*-(3,4,5-trimethoxyphenyl)sulfamoyl)phenyl)glycinate (**46**). Ethyl 2-bromoacetate (242 μL, 2.17 mmol) and NaI (65 mg, 0.43 mmol) were added to a solution of **42** (83 mg, 0.22 mmol) and K_2CO_3 (59 mg, 0.43 mmol) in acetone/THF 1:1 (40 mL). The reaction mixture was heated at 70 °C for 48 h. After cooling, the reaction mixture was concentrated and re-dissolved in EtOAc, washed with brine, dried over Na_2SO_4, filtered, and evaporated to dryness. The residue was purified by preparative TLC (hexane/EtOAc 4:6) to afford compound **46** (20 mg, 0.04 mmol, 19%). ^{1}H NMR (400 MHz, $CDCl_3$): δ 1.28 (3H, *t, J* = 7.2), 3.10 (3H, *s*), 3.72 (6H, *s*), 3.80 (2H, *s*), 3.82 (3H, *s*), 3.91 (3H, *s*), 4.22 (2H, *q, J* = 7.2), 6.28 (2H, *s*), 6.57 (1H, *d, J* = 2), 6.77 (1H, *d, J* = 8.4), 6.99 (1H, *dd, J* = 8.4 and 2). ^{13}C NMR (50 MHz, $CDCl_3$): δ 14.1 (CH_3), 38.4 (CH_3), 44.9 (CH_2), 55.8 (CH_3), 56.1 (2CH_3), 60.8 (CH_3), 61.4 (CH_2), 104.6 (2CH), 108.2 (CH), 108.3 (CH), 118.2 (CH), 128.2 (C), 136.9 (C), 137.2 (C), 137.5 (C), 150.1 (C), 152.8 (2C), 170.3 (C). HRMS ($C_{21}H_{28}N_2O_8S$ + H^+): calcd 469.1639 (M + H^+), found 469.1632.

Ethyl (5-(*N*-(2-ethoxy-2-oxoethyl)-*N*-(3,4,5-trimethoxyphenyl)sulfamoyl)-2-methoxyphenyl) glycinate (**47**). Ethyl 2-bromoacetate (72.6 μL, 0.65 mmol) was added after 30 min to a solution of **41** (80 mg, 0.22 mmol) and K_2CO_3 (59 mg, 0.43 mmol) in dry DMF (3 mL). The reaction mixture was then stirred for 48 h, concentrated under vacuum, re-dissolved in EtOAc, washed with brine, dried (Na_2SO_4), and evaporated to dryness. The residue was purified by preparative TLC (CH_2Cl_2/EtOAc 7:3) to give **47** (34 mg, 0.06 mmol, 29%). ^{1}H NMR (400 MHz, $CDCl_3$): δ 1.22 (3H, *t, J* = 7.2), 1.28 (3H, *t, J* = 7.2), 3.69 (6H, *s*), 3.79 (3H, *s*), 3.84 (2H, *s*), 3.89 (3H, *s*), 4.15 (2H, *q, J* = 7.2), 4.22 (2H, *q, J* = 7.2), 4.31 (2H, *s*), 6.38 (2H, *s*), 6.71 (1H, *d, J* = 2), 6.75 (1H, *d, J* = 8.4), 7.06 (1H, *dd, J* = 8.4 and 2). ^{13}C NMR (100 MHz, $CDCl_3$): δ 14.1 (CH_3), 14.1 (CH_3), 45.0 (CH_2), 52.9 (CH_2), 55.8 (CH_3), 56.0 (2CH_3), 60.8 (CH_3), 61.3 (CH_2), 61.5 (CH_2), 106.4 (2CH), 108.2 (CH), 108.3 (CH), 118.2 (CH), 130.5 (C), 135.5 (C), 137.1 (C), 137.8 (C), 150.2 (C), 152.9 (2C), 168.8 (C), 170.3 (C). HRMS ($C_{24}H_{32}N_2O_{10}S$ + H^+): calcd 541.1850 (M + H^+), found 541.1830.

4-Methoxy-*N*-methyl-3-(methylamino)-*N*-(3,4,5-trimethoxyphenyl)benzenesulfonamide (**48a**) and 3-(dimethylamino)-4-methoxy-*N*-methyl-*N*-(3,4,5-trimethoxyphenyl) benzenesulfonamide (**48b**). $(CH_3)_2SO_4$ (272 μL, 2.85 mmol) was dropwise added to a solution of **41** (140 mg, 0.38 mmol) and K_2CO_3 (525 mg, 3.80 mmol) in acetone (20 mL) and heated under reflux overnight. Then, the reaction mixture was filtered, poured onto ice, and extracted with CH_2Cl_2. The organic layers were dried over anhydrous Na_2SO_4, filtered, and evaporated to dryness. Compounds **48a** (48 mg, 0.12 mmol, 32%) and **48b** (17 mg, 0.04 mmol, 11%) were isolated by preparative TLC (toluene/EtOAc 4:6). **48a:** IR (KBr): 3426, 1594, 1500, 803 cm^{-1}. ^{1}H NMR (400 MHz, $CDCl_3$): δ 2.76 (3H, *s*), 3.12 (3H, *s*), 3.74 (6H, *s*), 3.82 (3H, *s*), 3.89 (3H, *s*), 6.32 (2H, *s*), 6.63 (1H, *d, J* = 2), 6.75 (1H, *d, J* = 8), 6.99 (1H, *dd, J* = 8 and 2). ^{13}C NMR (100 MHz,

$CDCl_3$): δ 30.0 (CH_3), 38.4 (CH_3), 55.7 (CH_3), 56.0 ($2CH_3$), 60.8 (CH_3), 104.7 (2CH), 107.7 (CH), 107.8 (CH), 117.1 (CH), 128.4 (C), 137.3 (C), 137.7 (C), 139.1 (C), 149.9 (C), 152.8 (2C). HRMS ($C_{18}H_{24}N_2O_6S + H^+$): calcd 397.1428 ($M + H^+$), found 397.1432. **48b:** ^1H NMR (400 MHz, $CDCl_3$): δ 2.64 (6H, *s*), 3.05 (3H, *s*), 3.66 (6H, *s*), 3.75 (3H, *s*), 3.88 (3H, *s*), 6.23 (2H, *s*), 6.82 (1H, *d, J* = 8.4), 6.89 (1H, *d, J* = 2), 7.24 (1H, *dd, J* = 8.4 and 2). ^{13}C NMR (100 MHz, $CDCl_3$): δ 37.9 (CH_3), 42.5 ($2CH_3$), 55.4 (CH_3), 55.7 ($2CH_3$), 60.5 (CH_3), 104.1 (2CH), 109.7 (CH), 117.3 (CH), 122.5 (CH), 127.4 (C), 136.8 (C), 137.1 (C), 141.9 (C), 152.5 (2C), 155.2 (C). HRMS ($C_{19}H_{26}N_2O_6S + H^+$): calcd 411.1584 ($M + H^+$), found 411.1570.

SP2

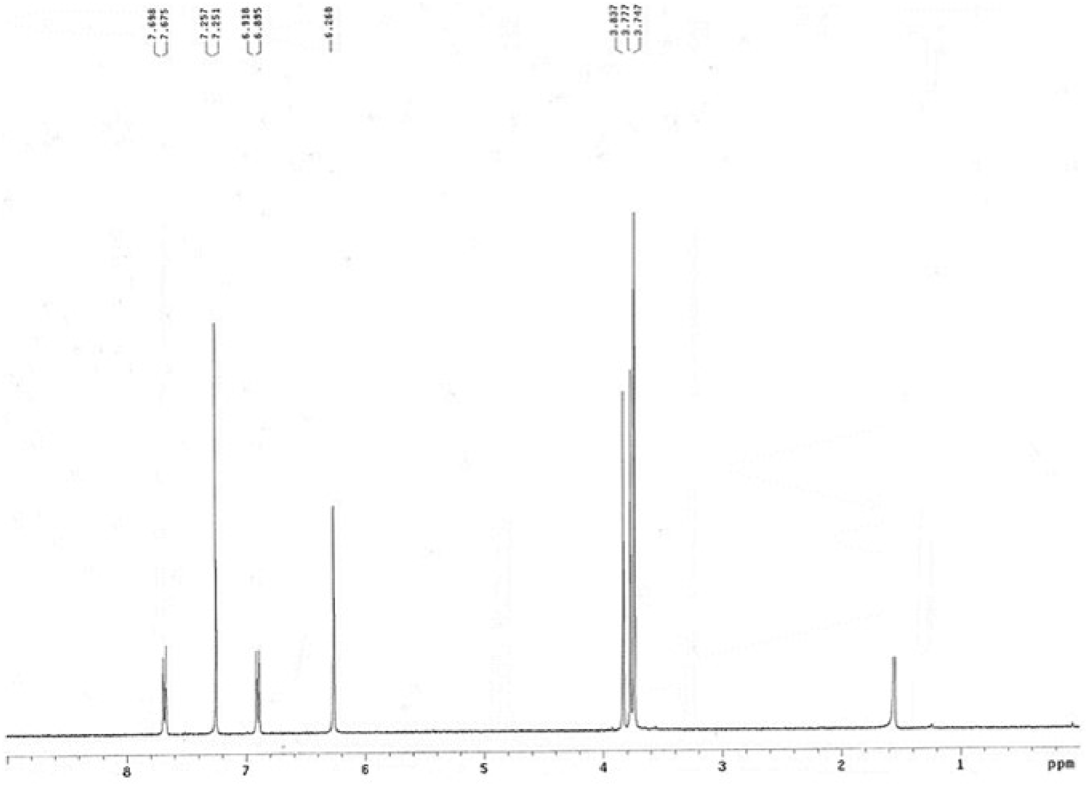

Fig. 1a: Compound **1a** ^{1}H NMR spectra.

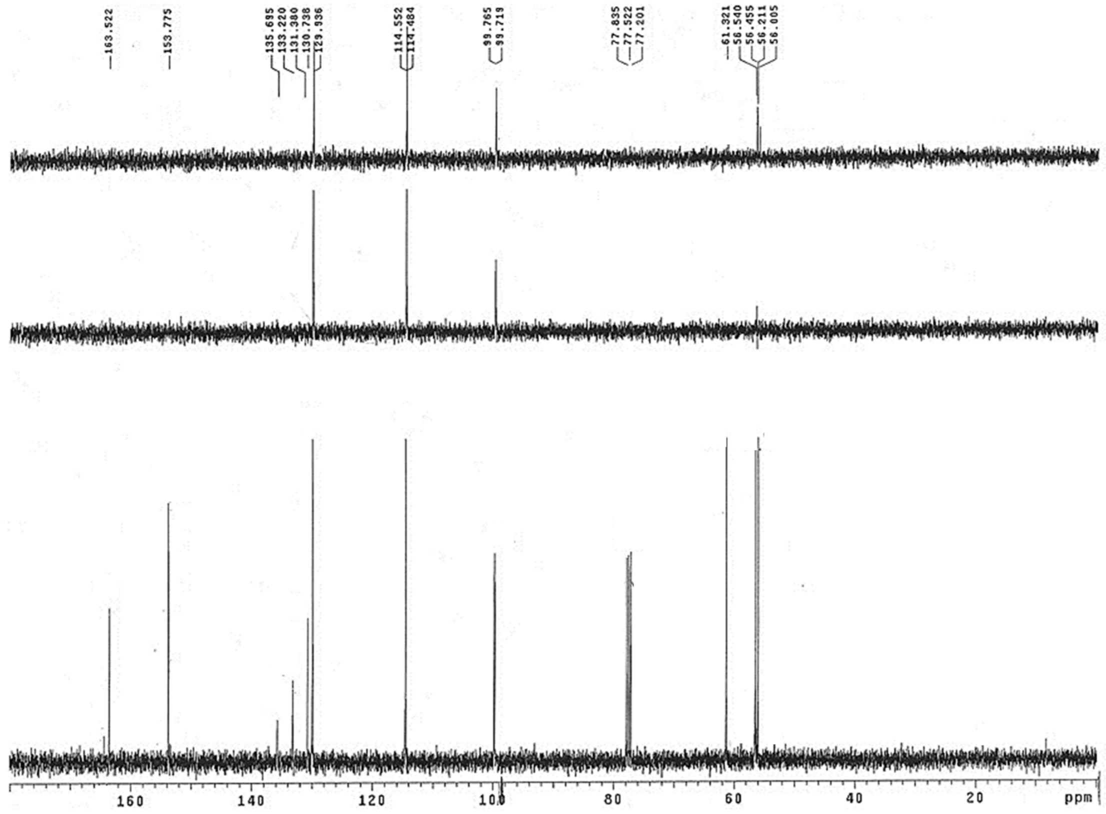

Fig. 1b: Compound **1a** ^{13}C NMR spectra.

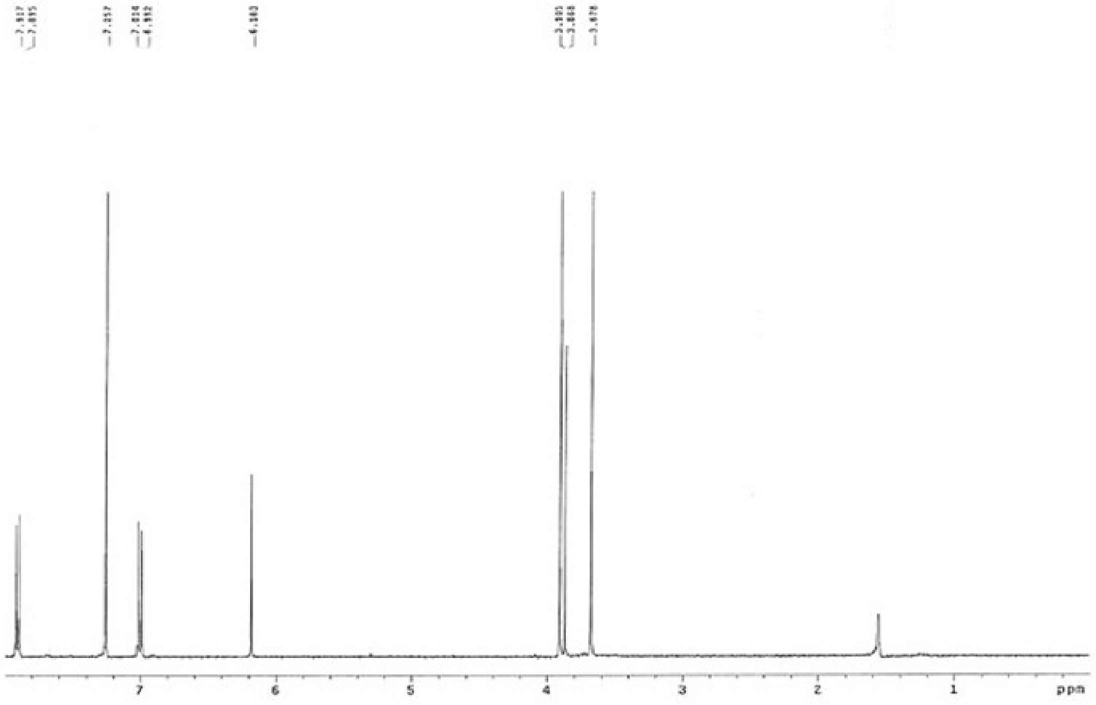

Fig. 2a: Compound **1b** ^{1}H NMR spectra.

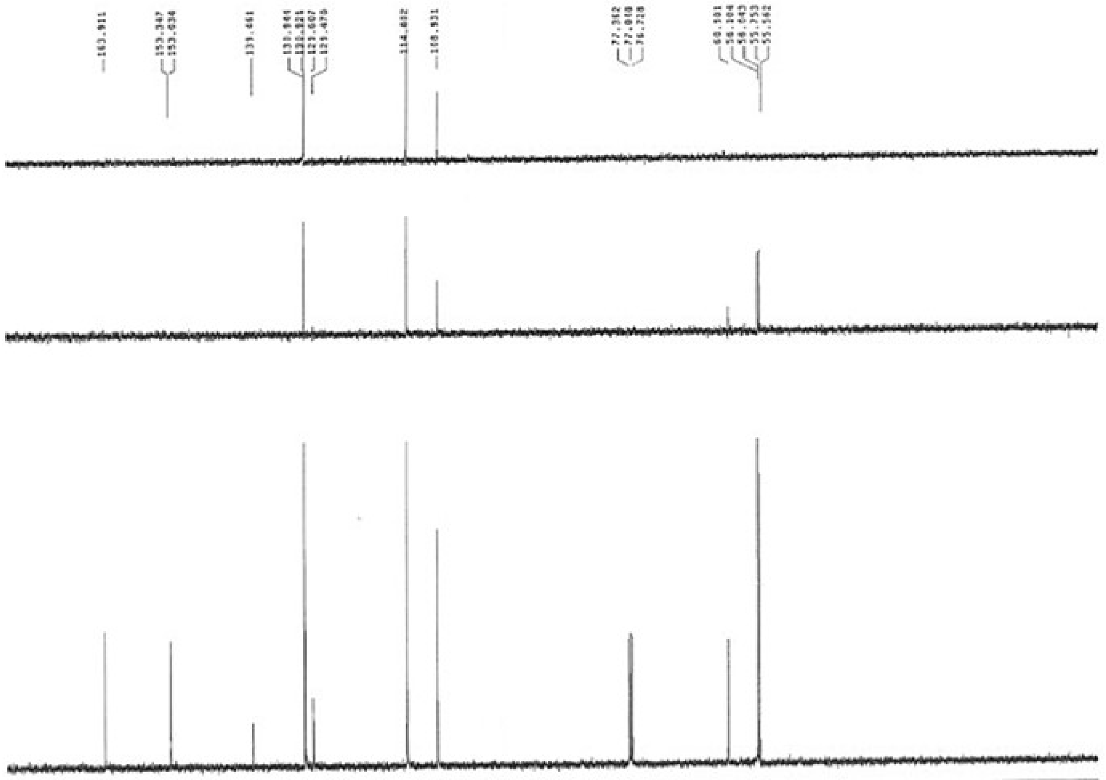

Fig. 2b: Compound **1b** ^{13}C NMR spectra.

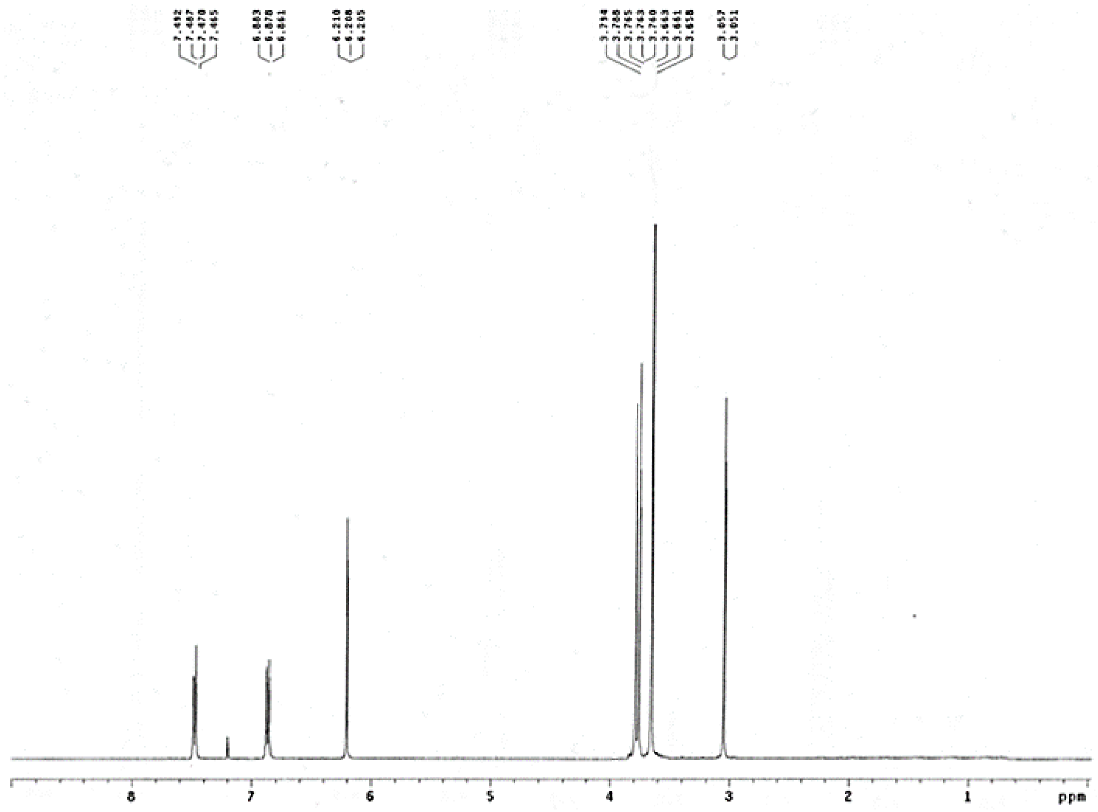

Fig. 3a: Compound **2a** ^{1}H NMR spectra.

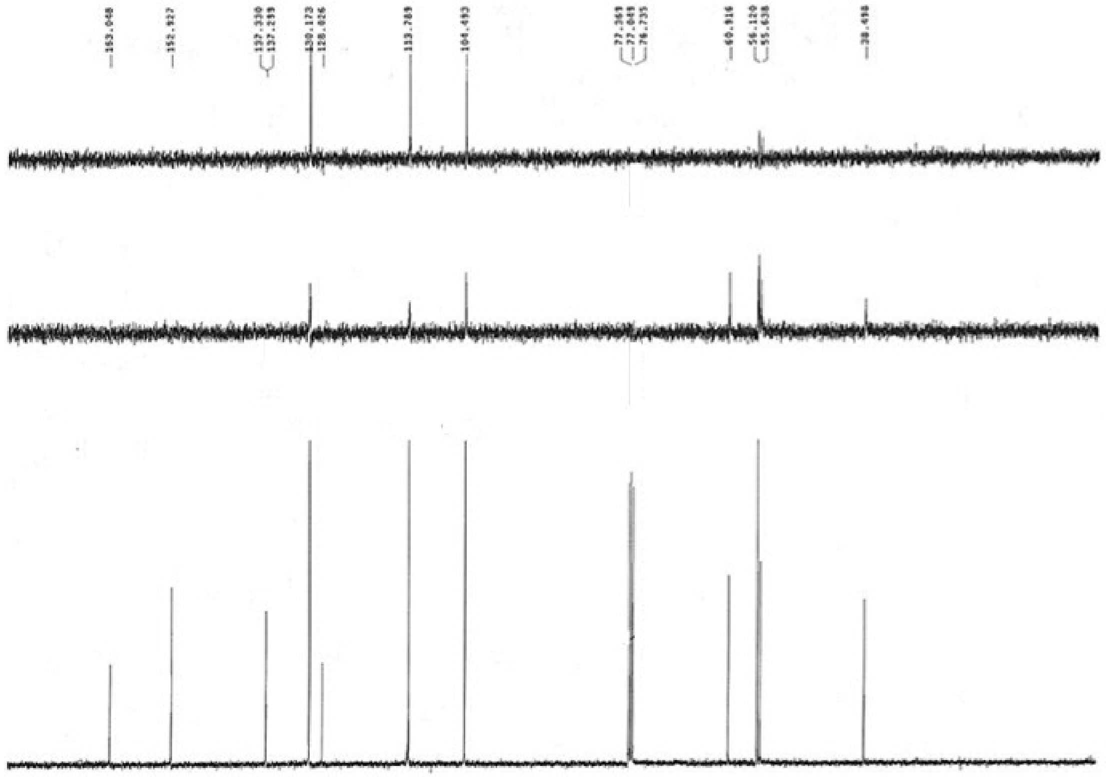

Fig. 3b: Compound **2a** ^{13}C NMR spectra.

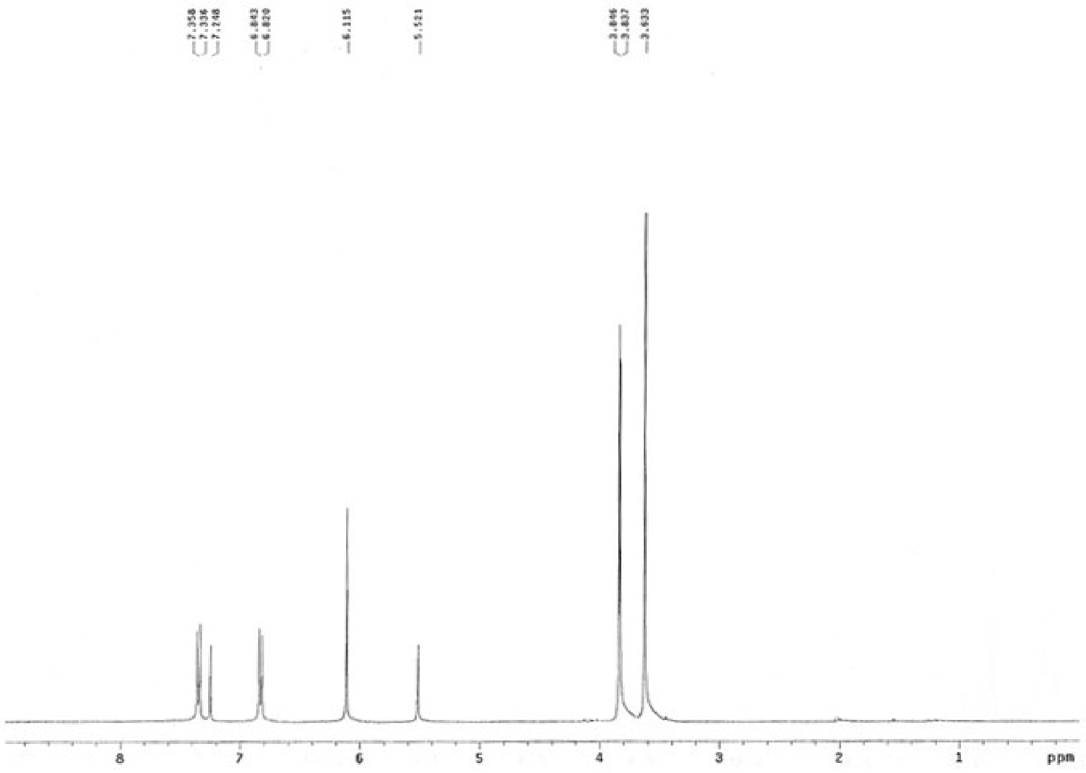

Fig. 4a: Compound **2b** ^{1}H NMR spectra.

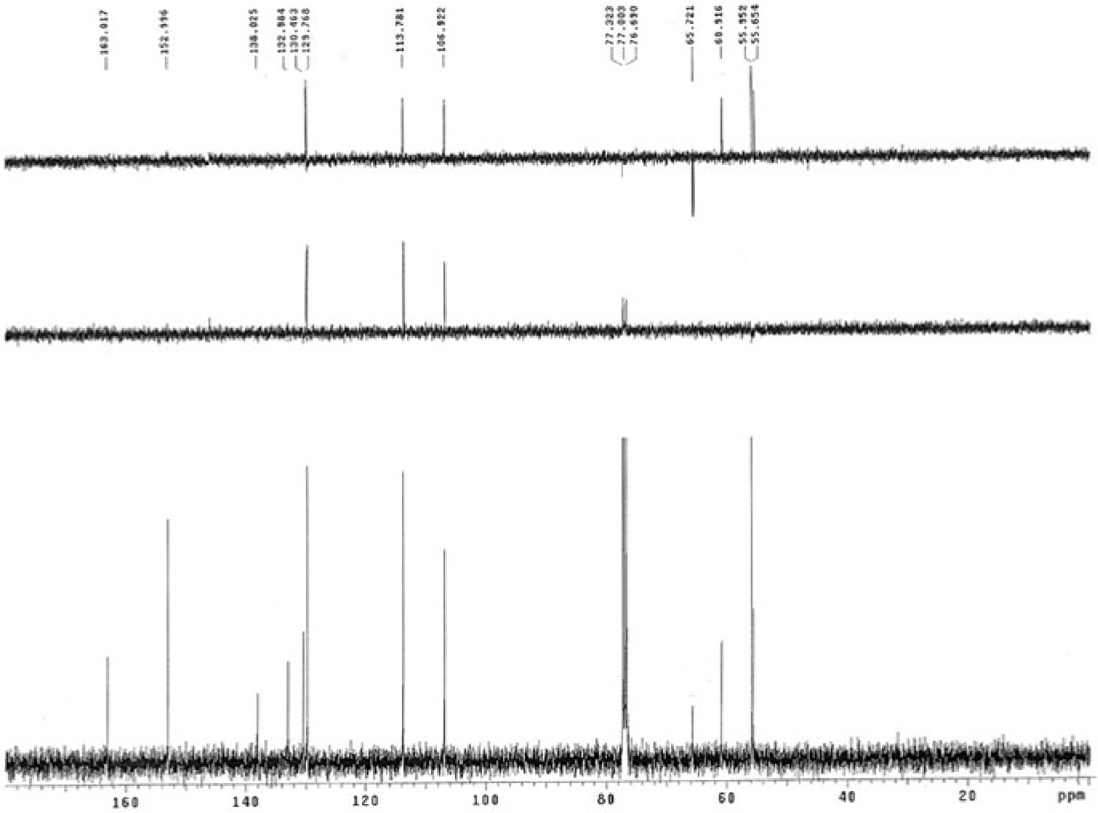

Fig. 4b: Compound **2b** ^{13}C NMR spectra.

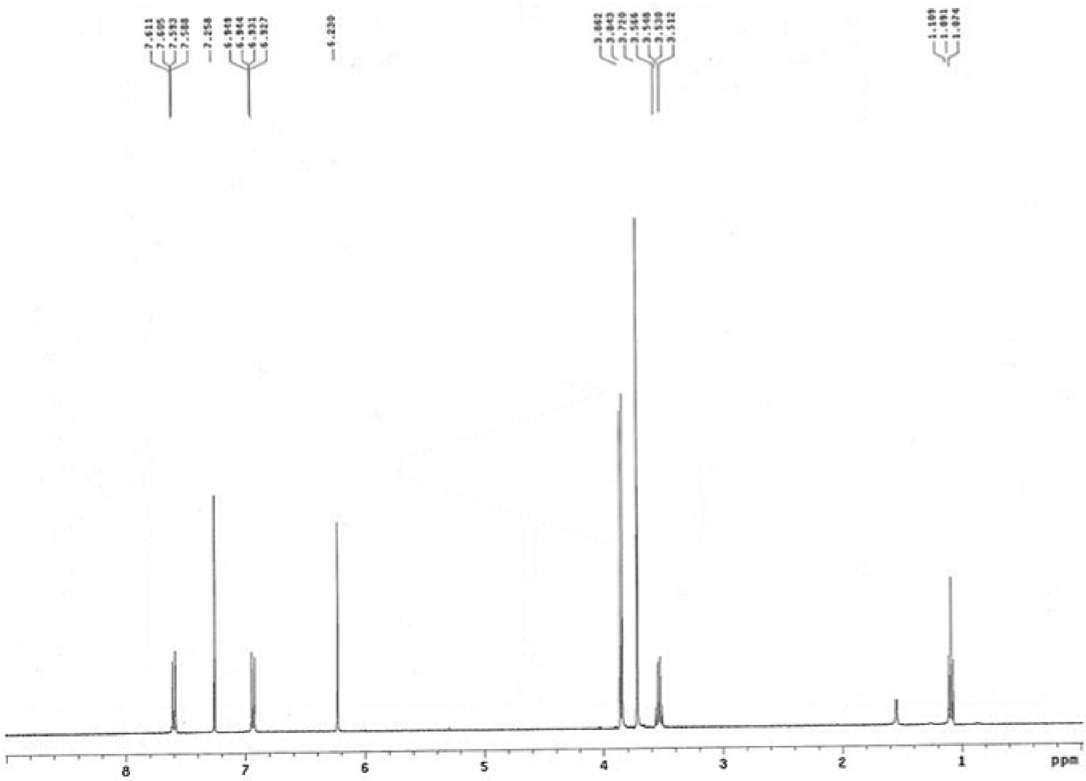

Fig. 5a: Compound **3** ^{1}H NMR spectra.

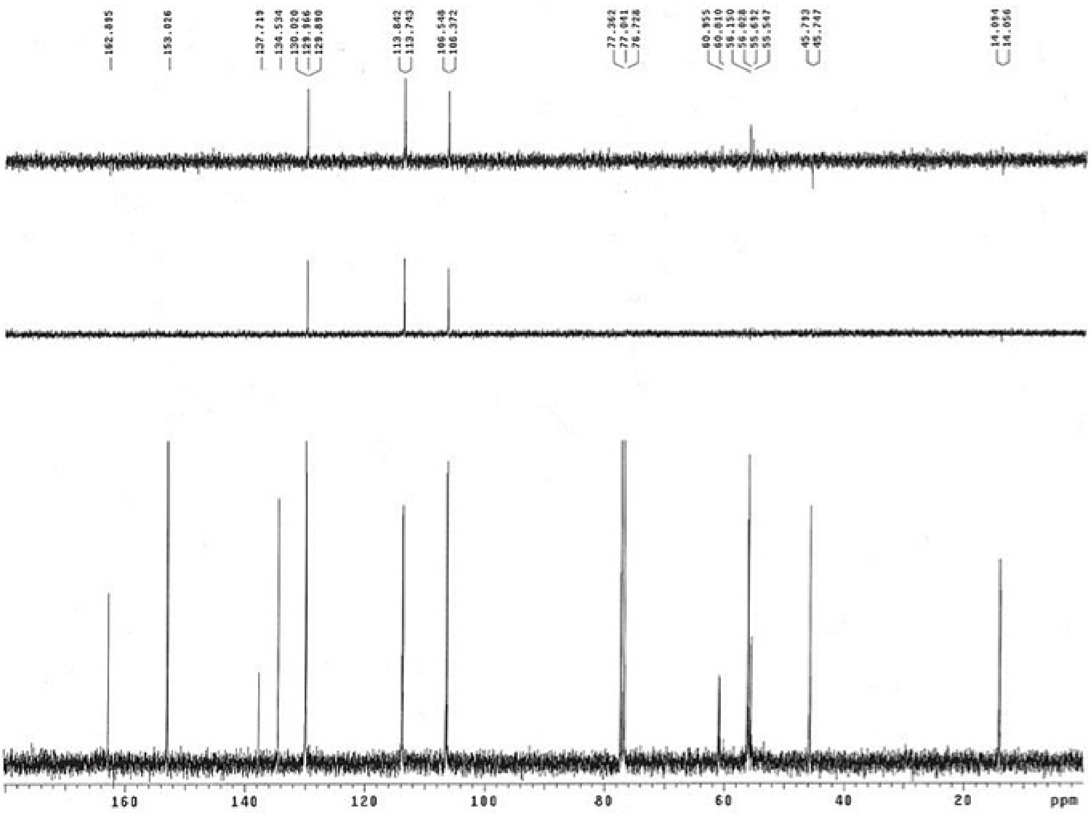

Fig. 5b: Compound **3** ^{13}C NMR spectra.

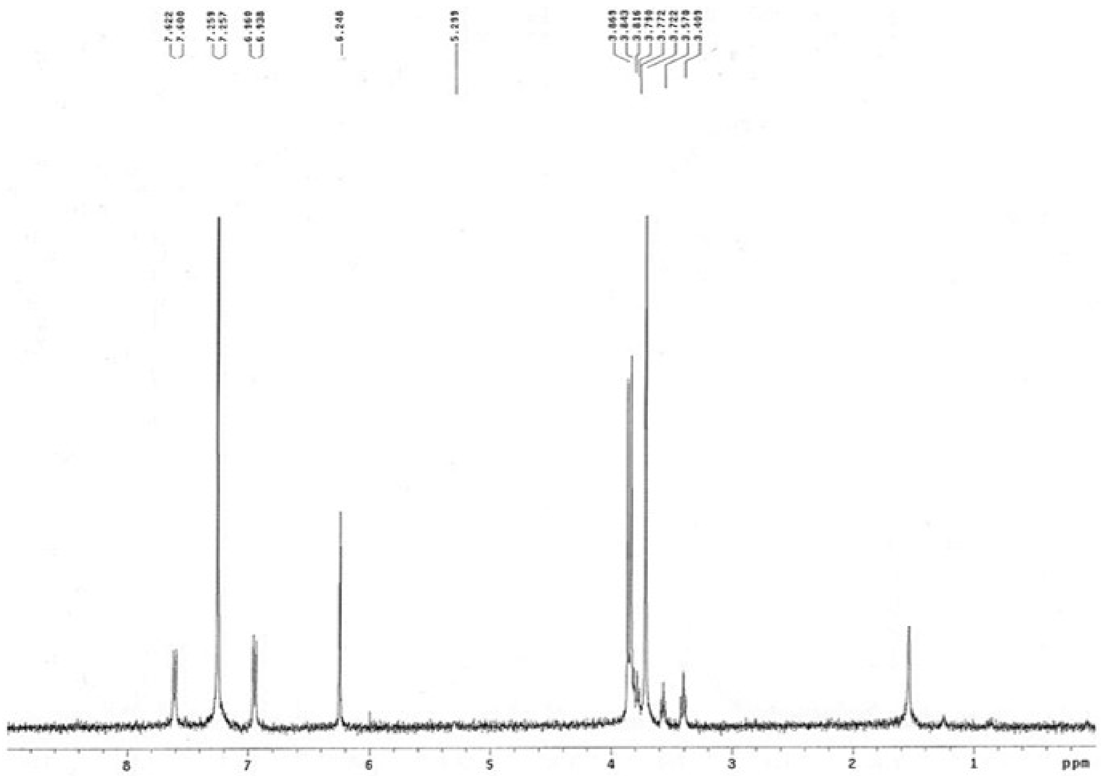

Fig. 6a: Compound **4** ^{1}H NMR spectra.

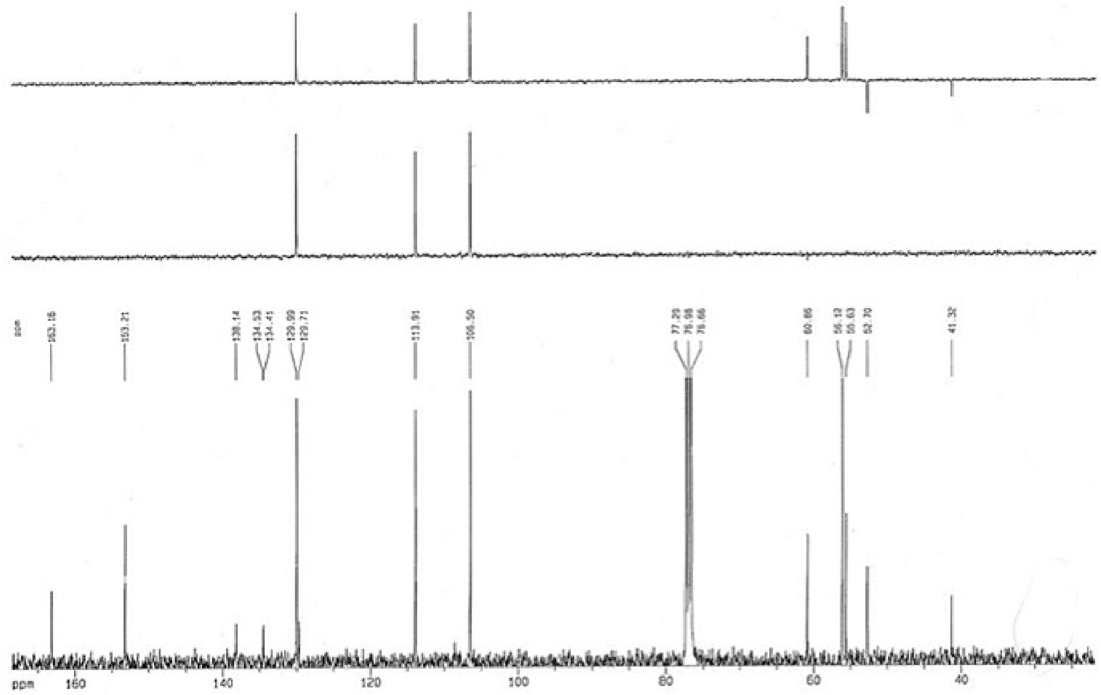

Fig. 6b: Compound **4** ^{13}C NMR spectra.

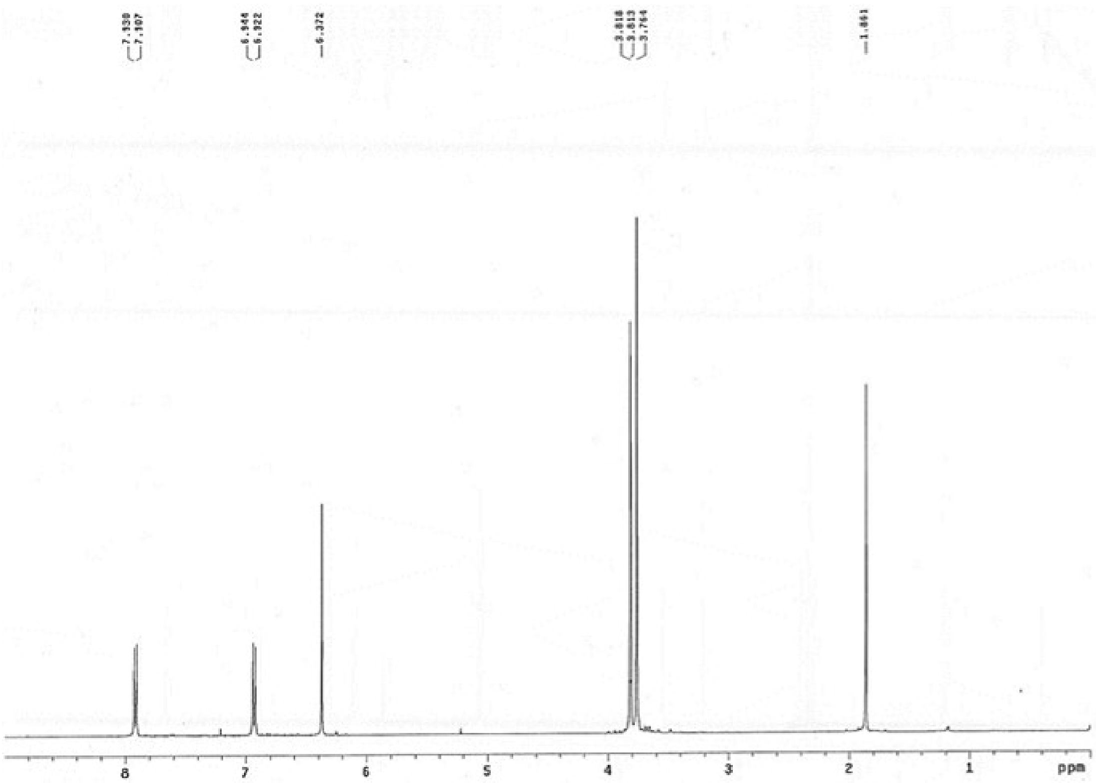

Fig. 7a: Compound **5** ^{1}H NMR spectra.

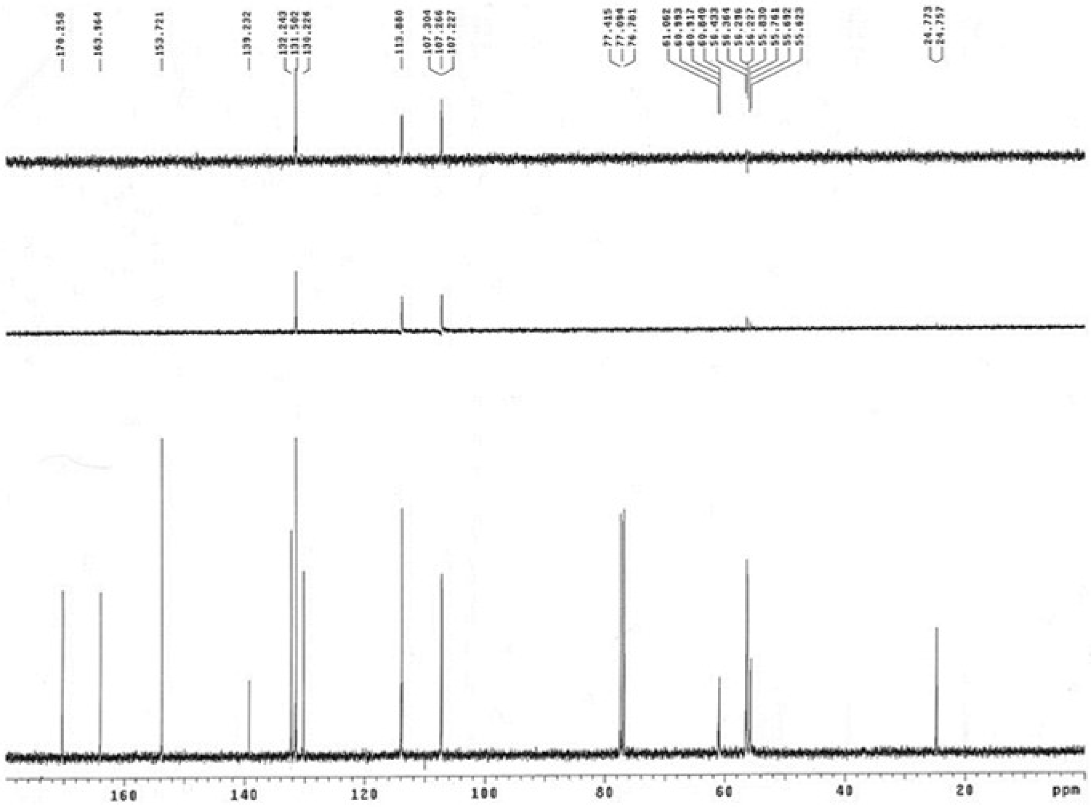

Fig. 7b: Compound **5** ^{13}C NMR spectra.

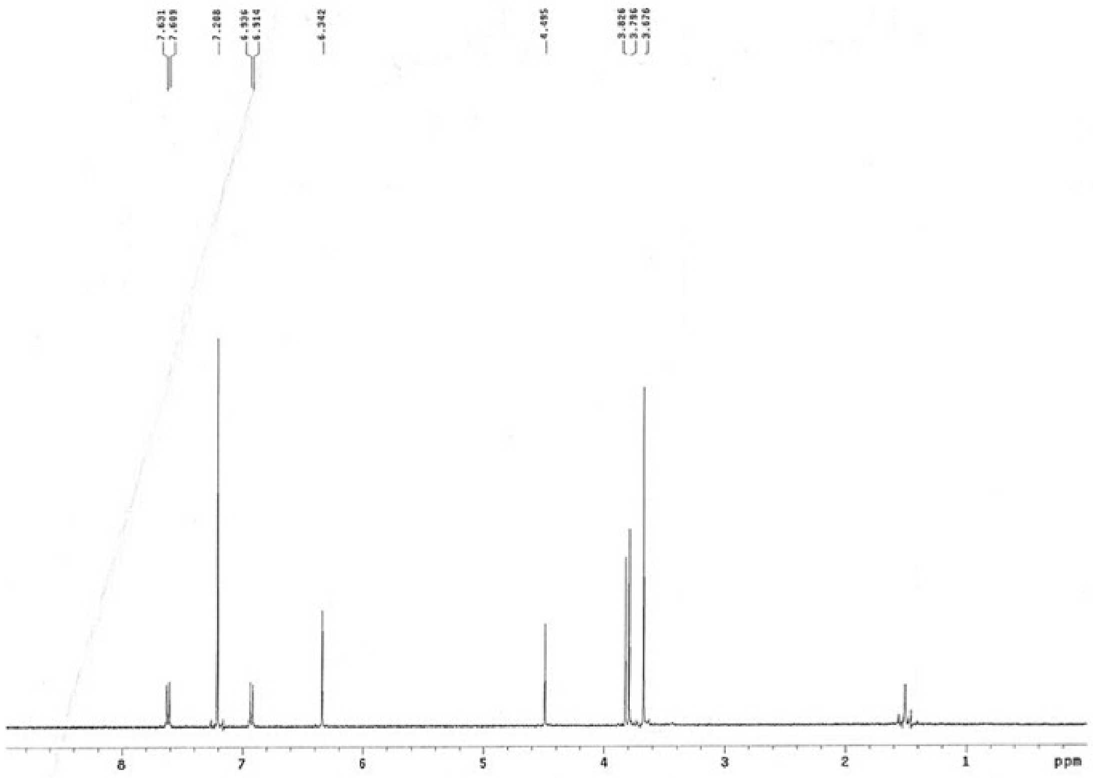

Fig. 8a: Compound **6** ^{1}H NMR spectra.

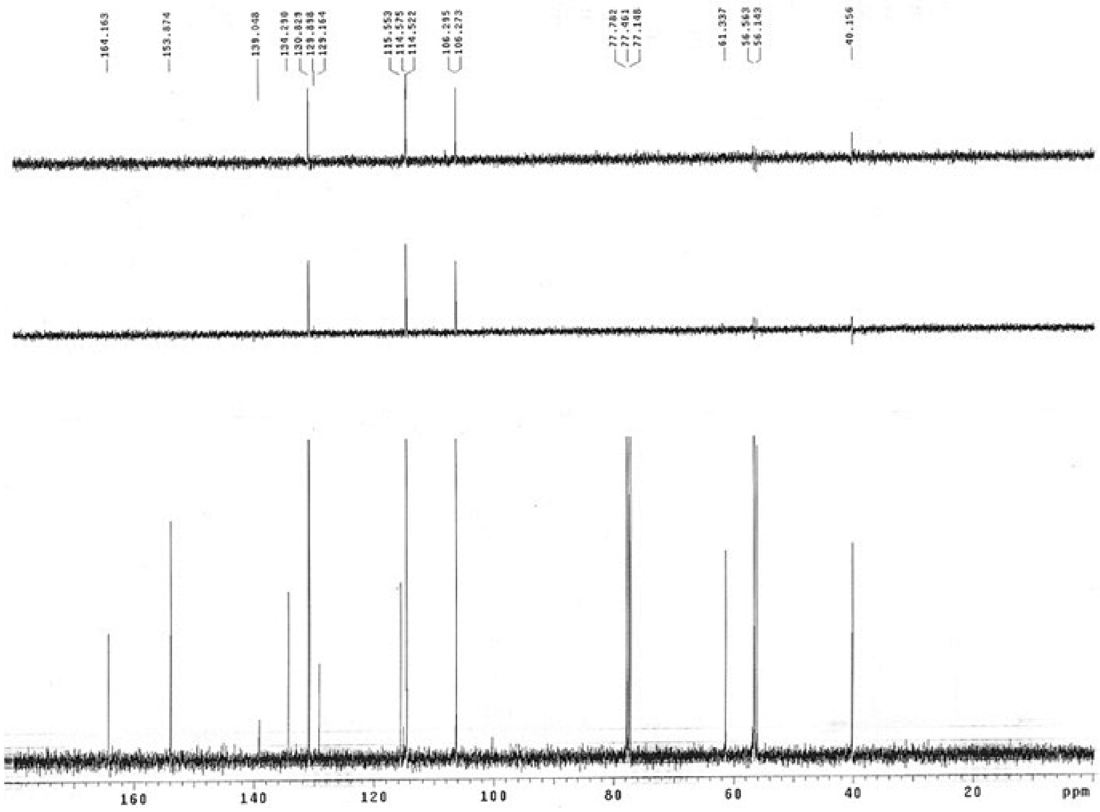

Fig. 8b: Compound **6** ^{13}C NMR spectra.

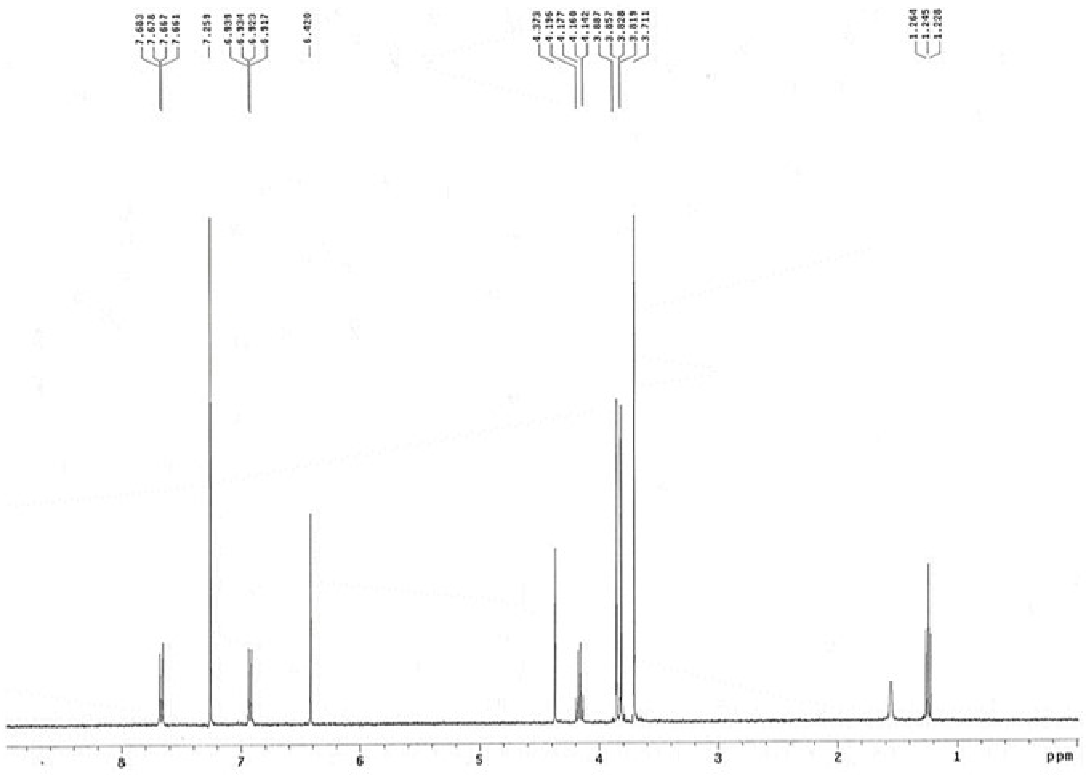

Fig. 9a: Compound **7** ^{1}H NMR spectra.

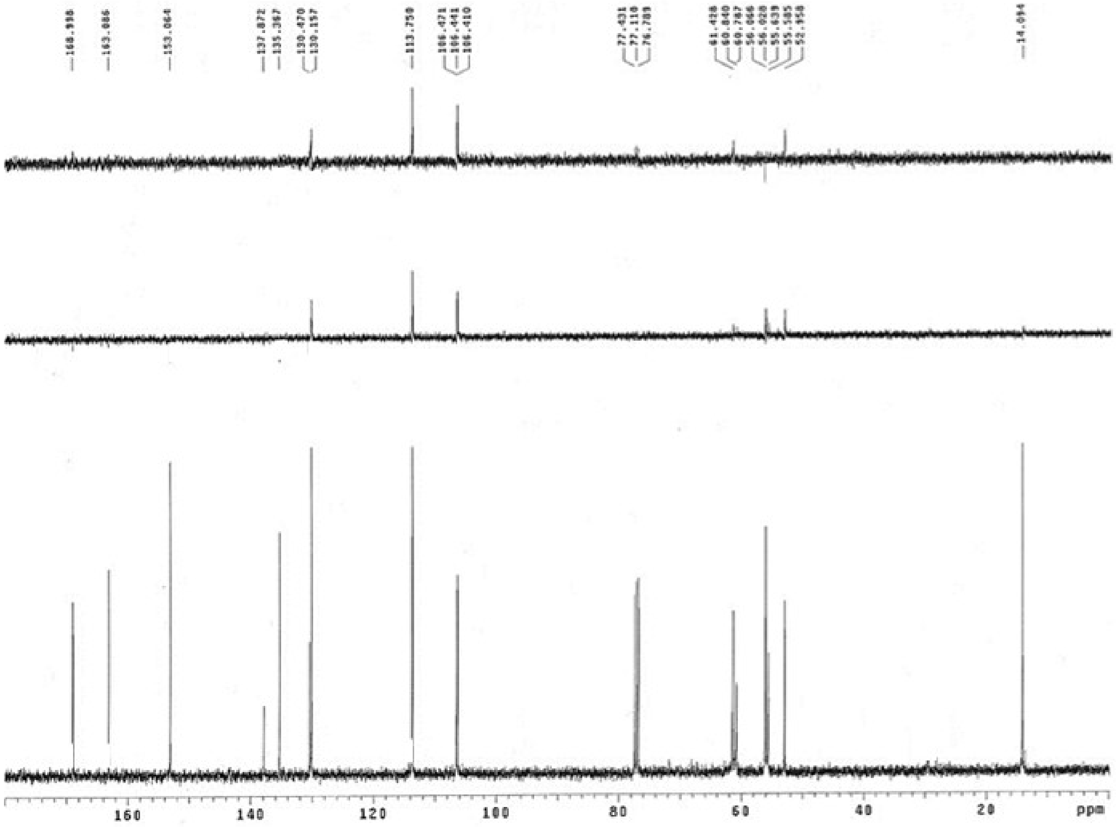

Fig. 9b: Compound **7** ^{13}C NMR spectra.

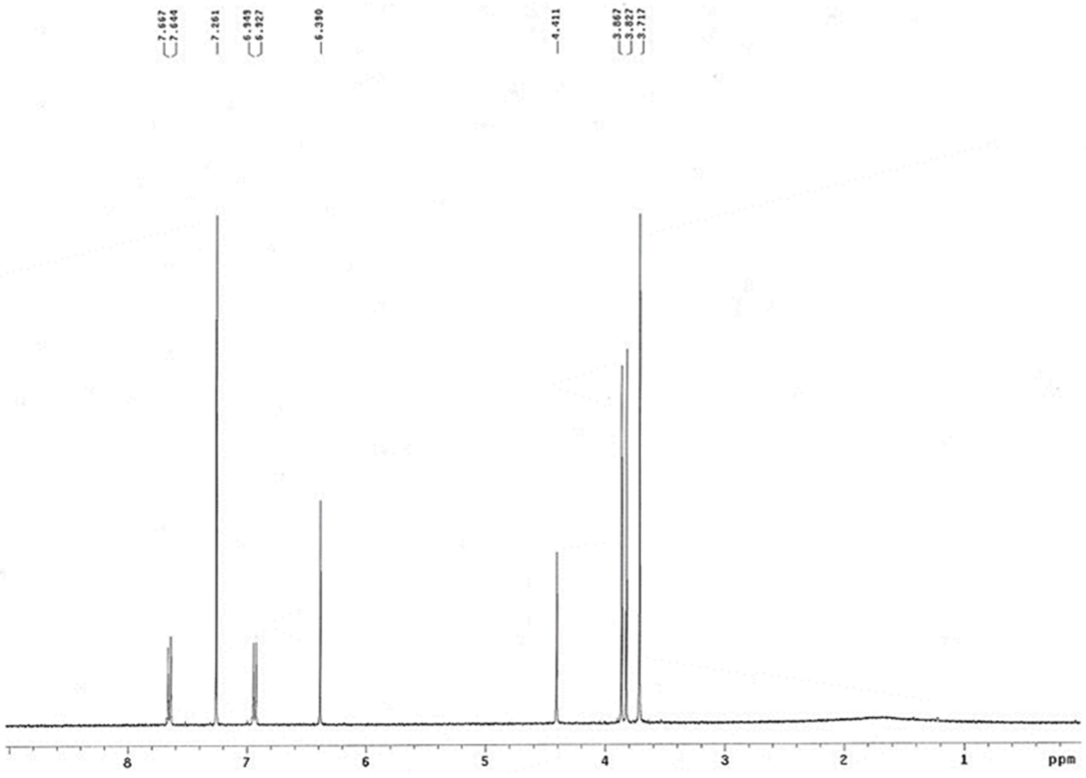

Fig. 10a: Compound **8** ^{1}H NMR spectra.

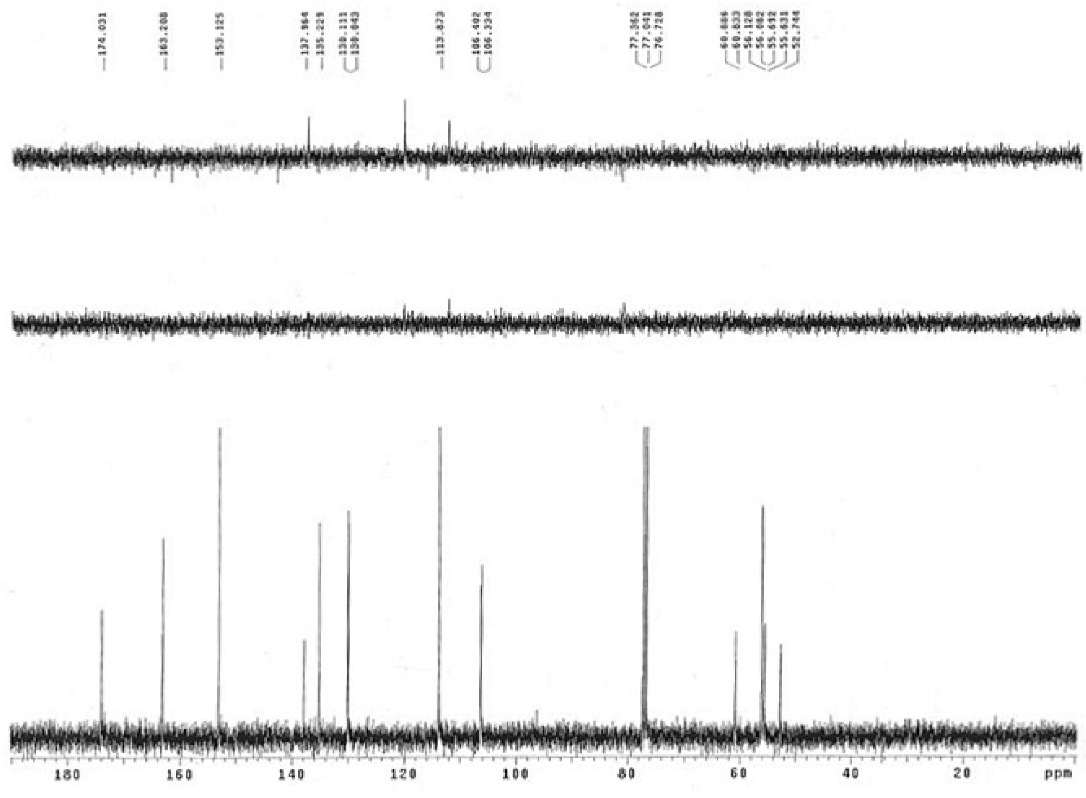

Fig. 10b: Compound **8** ^{13}C NMR spectra.

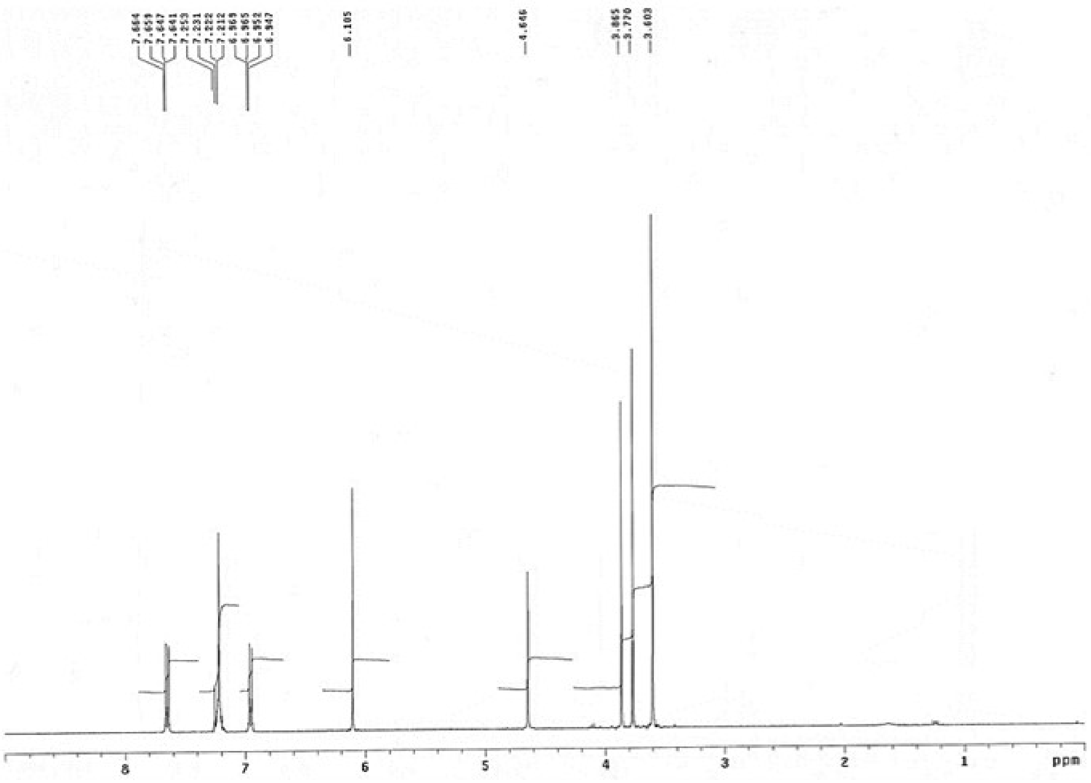

Fig. 11a: Compound **9** ^{1}H NMR spectra.

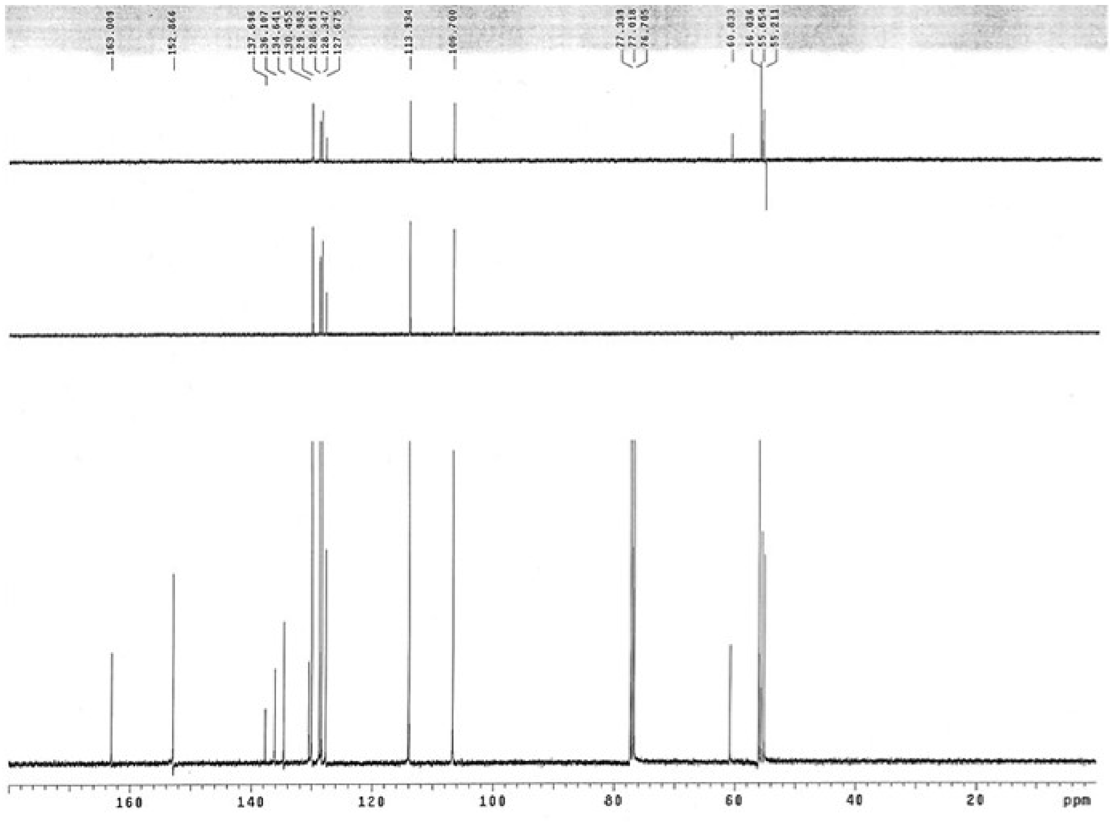

Fig. 11b: Compound **9** ^{13}C NMR spectra.

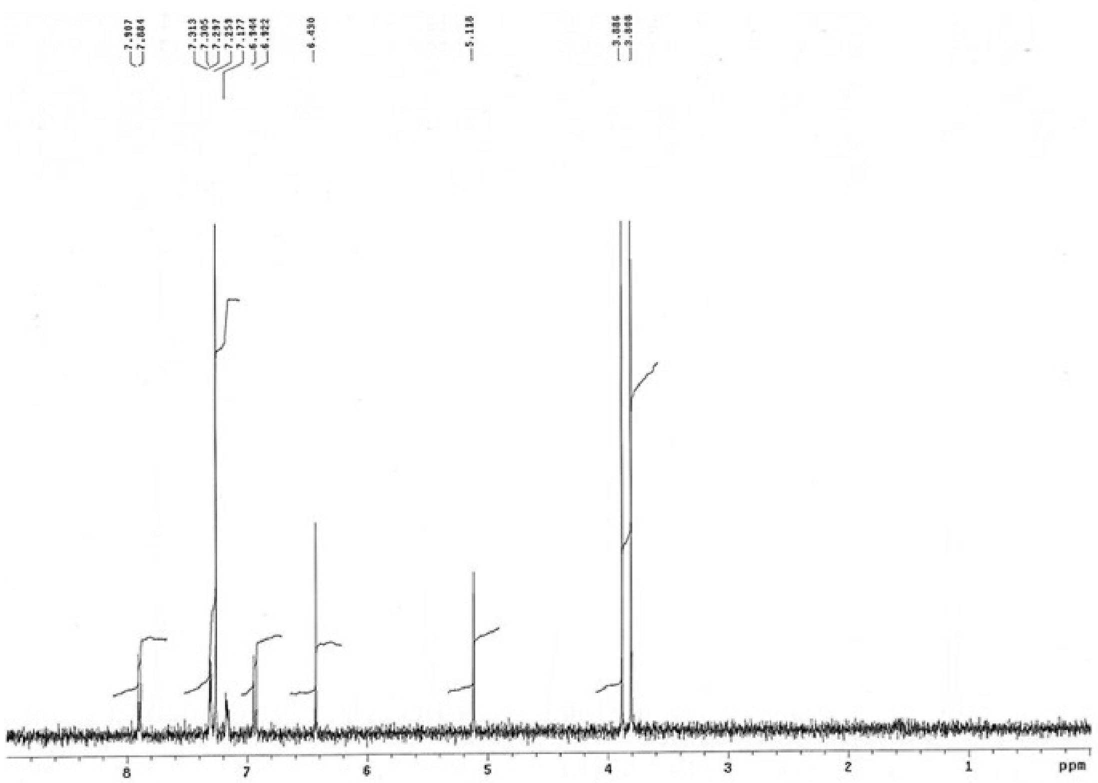

Fig. 12a: Compound **10** ^{1}H NMR spectra.

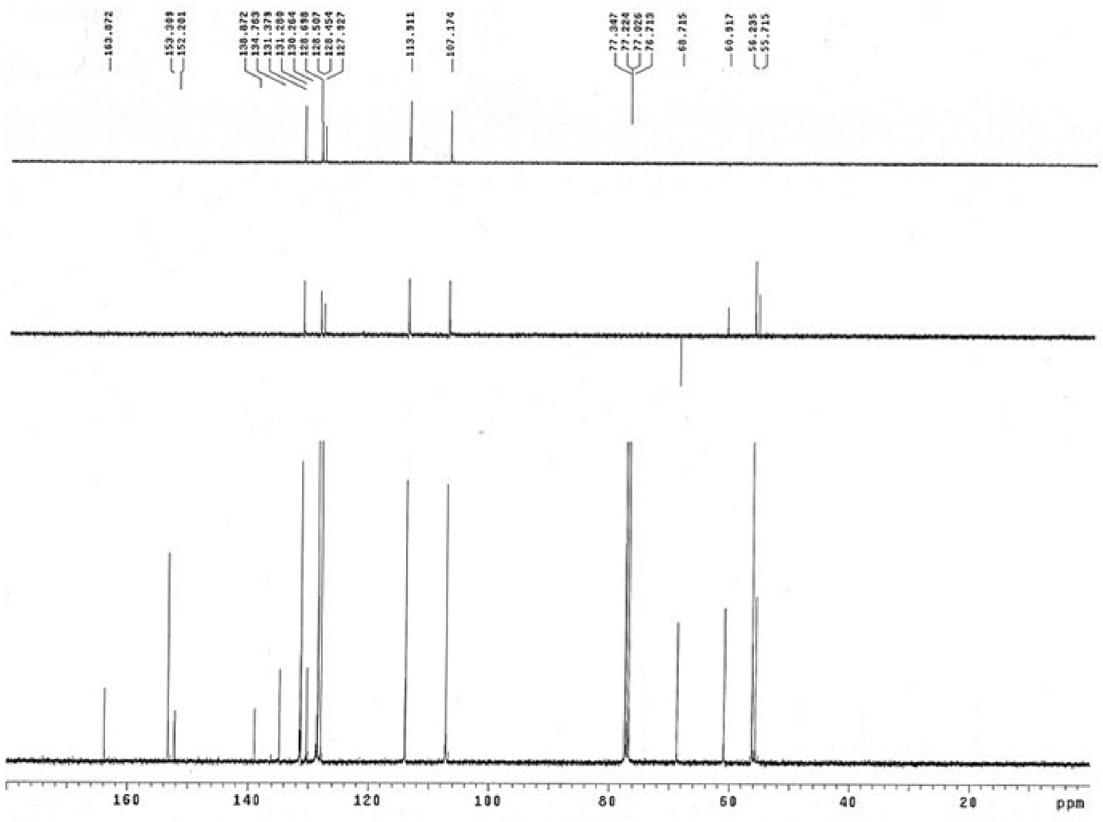

Fig. 12b: Compound **10** ^{13}C NMR spectra.

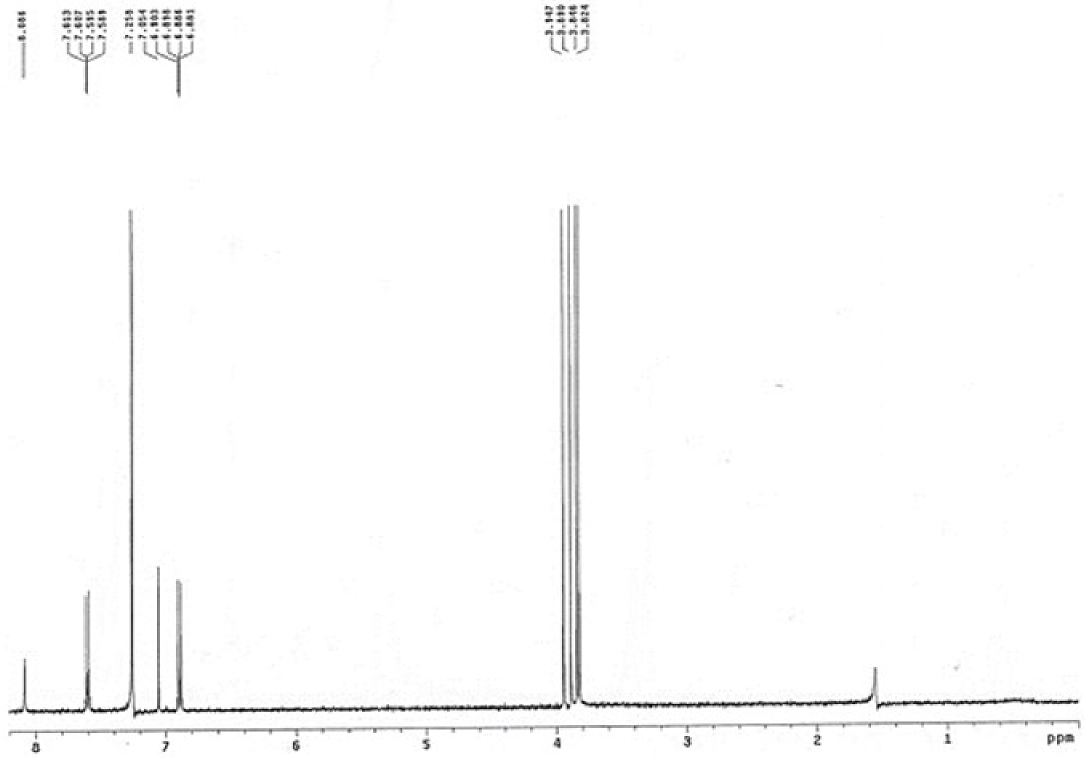

Fig. 13a: Compound **11** ^{1}H NMR spectra.

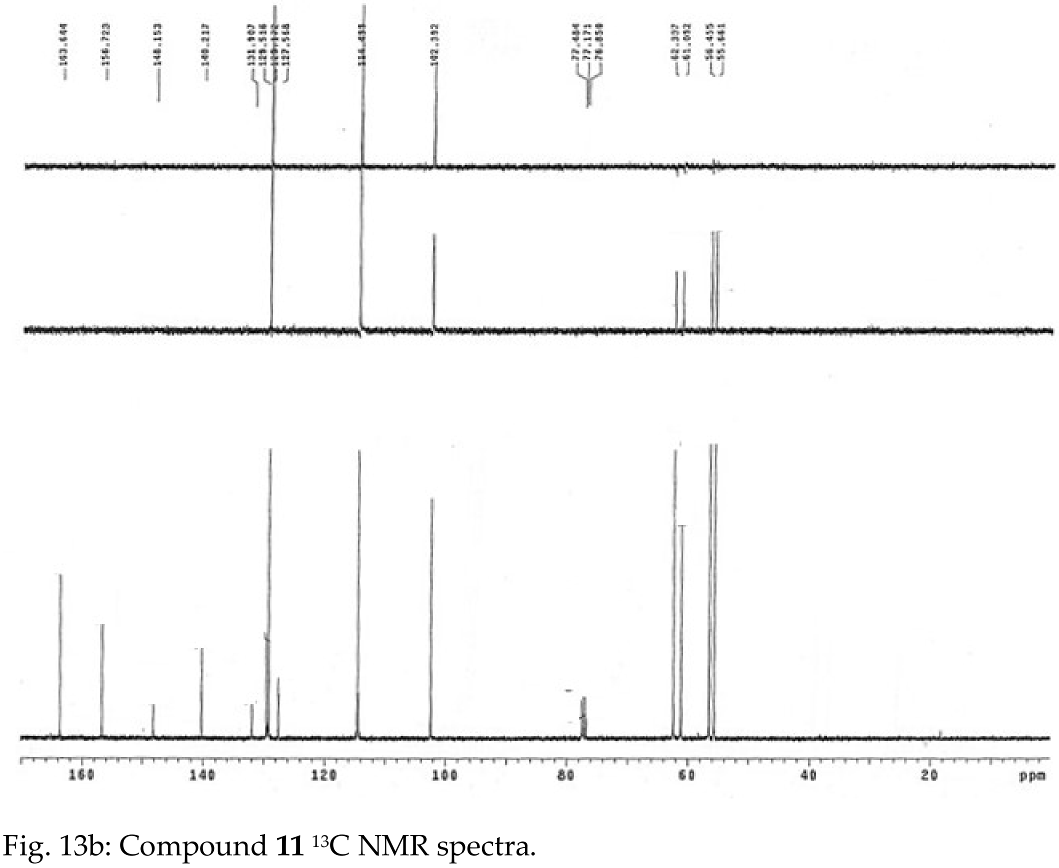

Fig. 13b: Compound **11** ^{13}C NMR spectra.

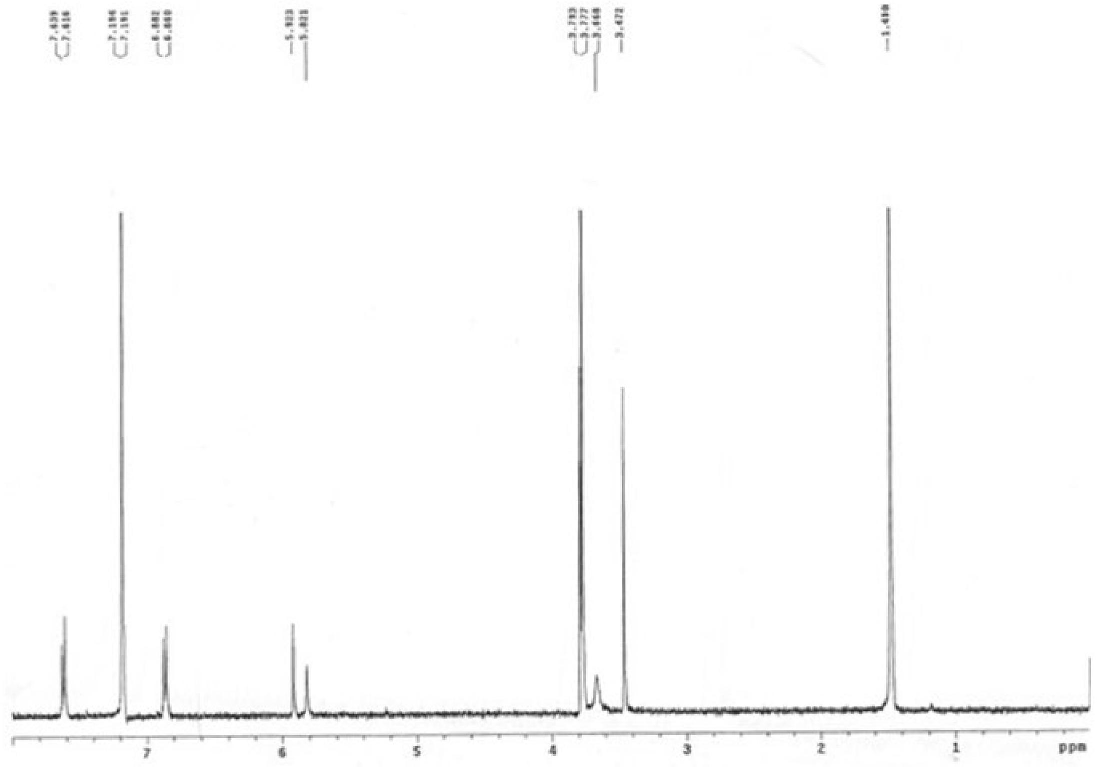

Fig. 14a: Compound **12** ^{1}H NMR spectra.

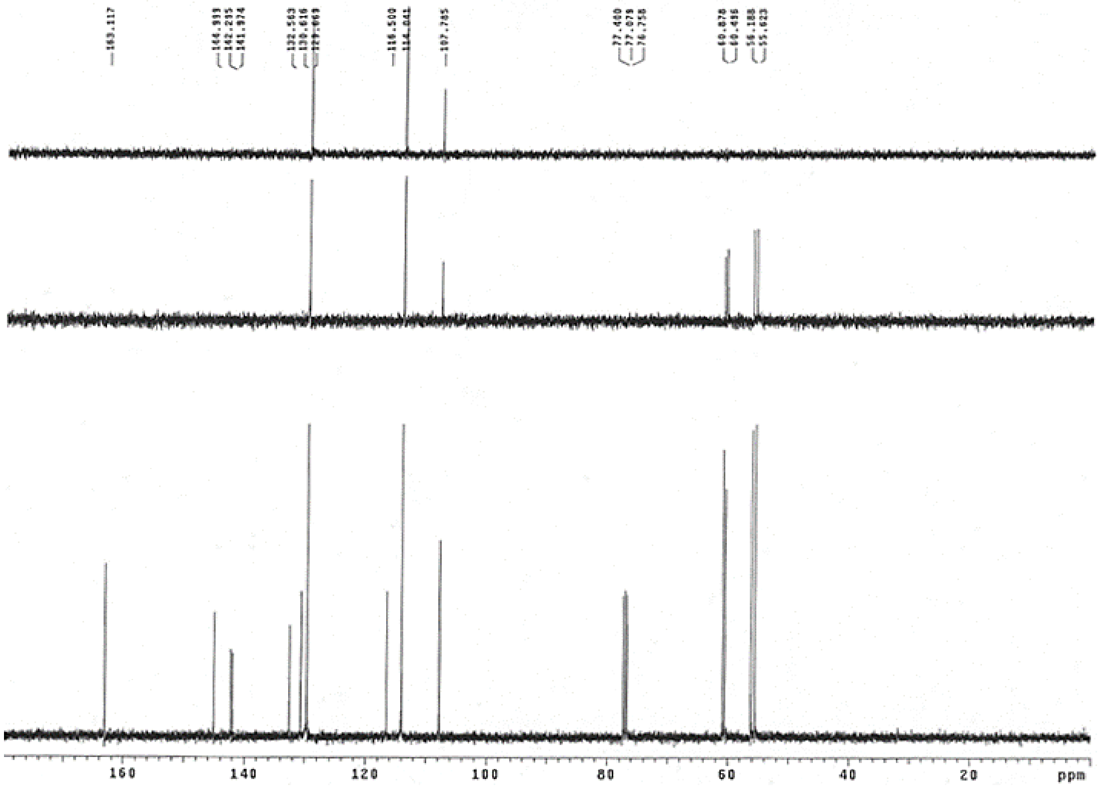

Fig. 14b: Compound **12** ^{13}C NMR spectra.

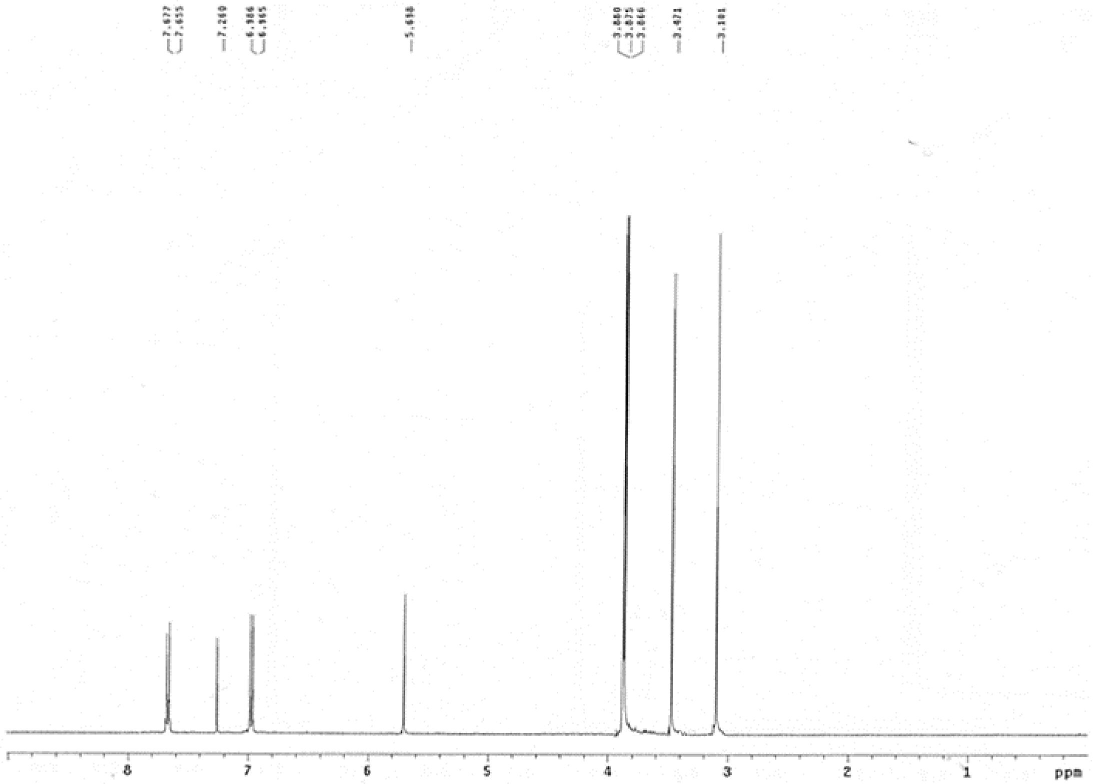

Fig. 15a: Compound **13** ^{1}H NMR spectra-

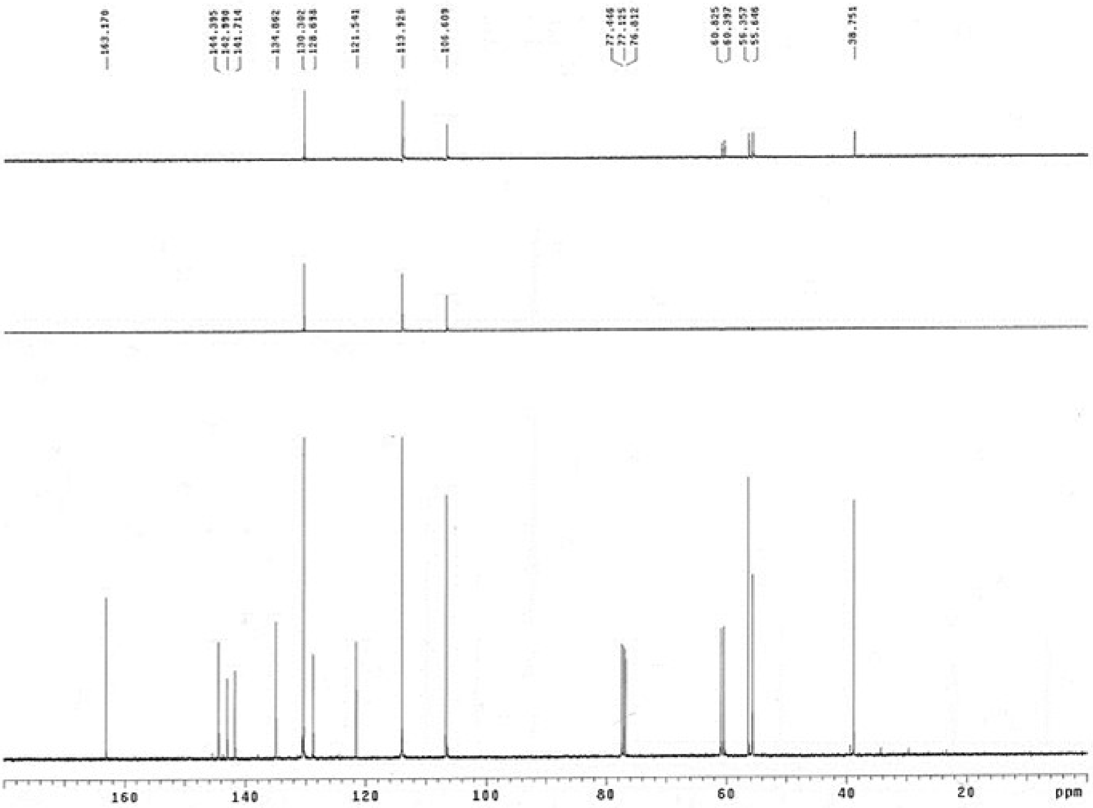

Fig. 15b: Compound **13** ^{13}C NMR spectra.

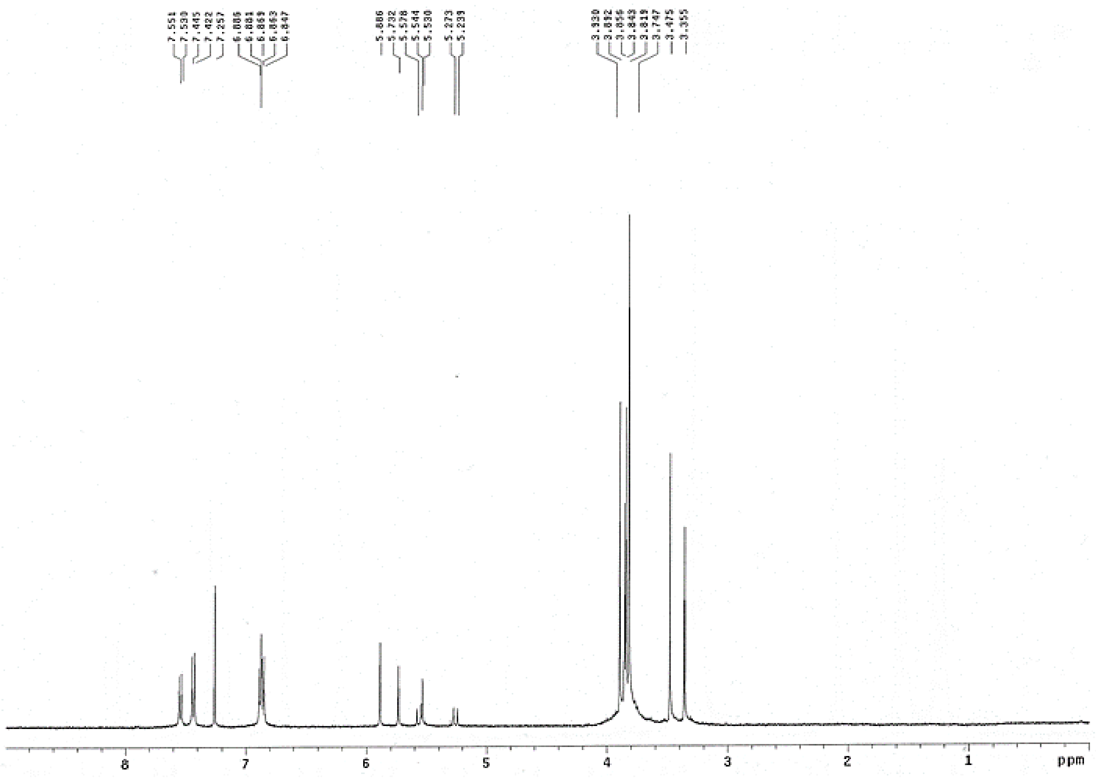

Fig. 16a: Compound **14** ^{1}H NMR spectra.

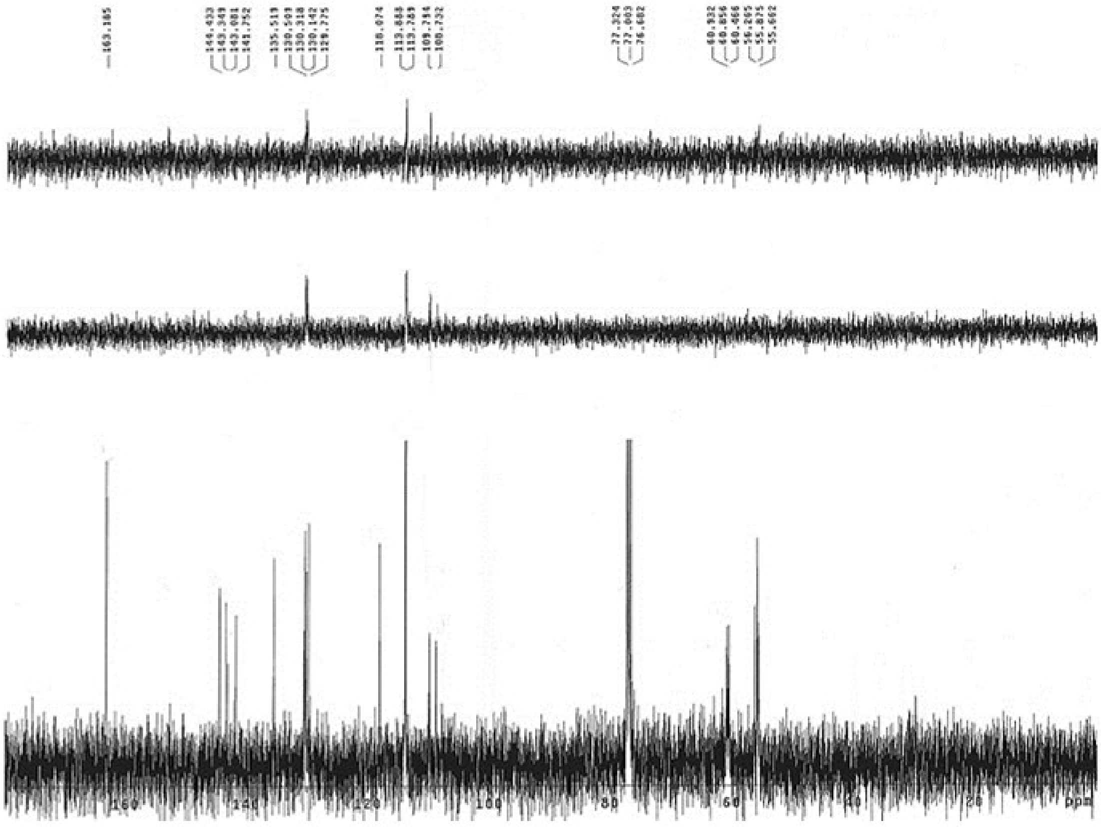

Fig. 16b: Compound **14** ^{13}C NMR spectra.

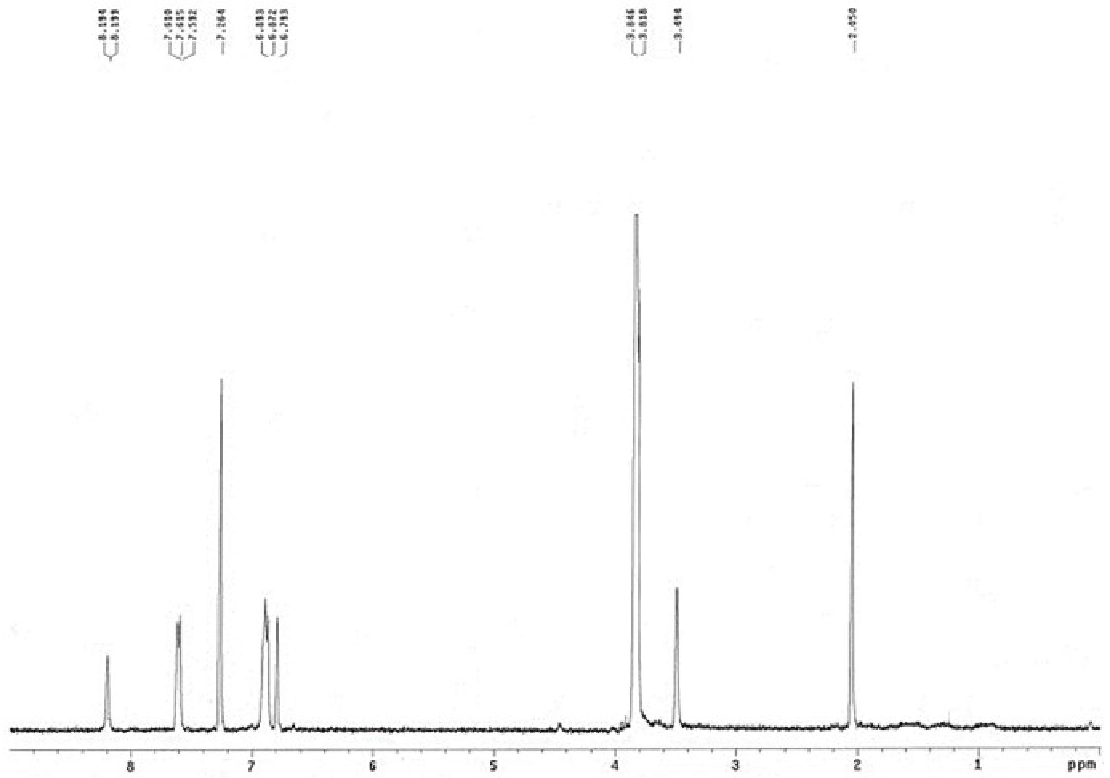

Fig. 17a: Compound **15** ^{1}H NMR spectra.

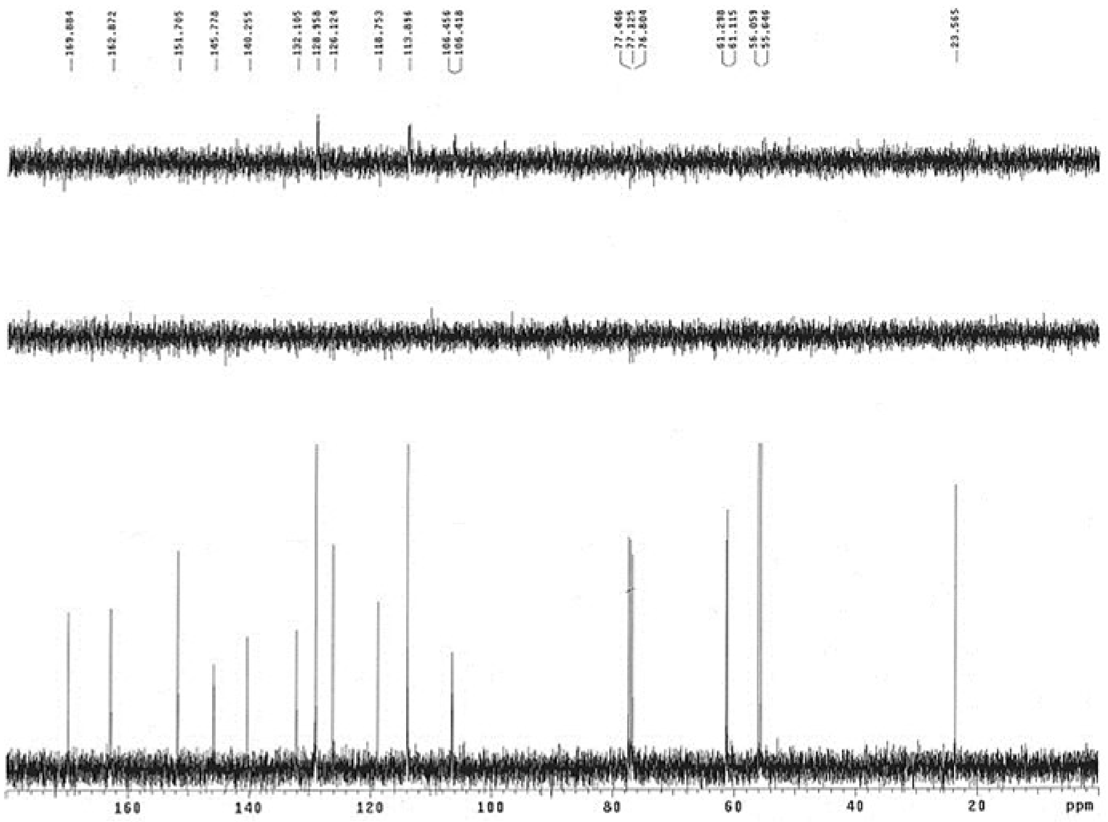

Fig. 17b: Compound **15** ^{13}C NMR spectra.

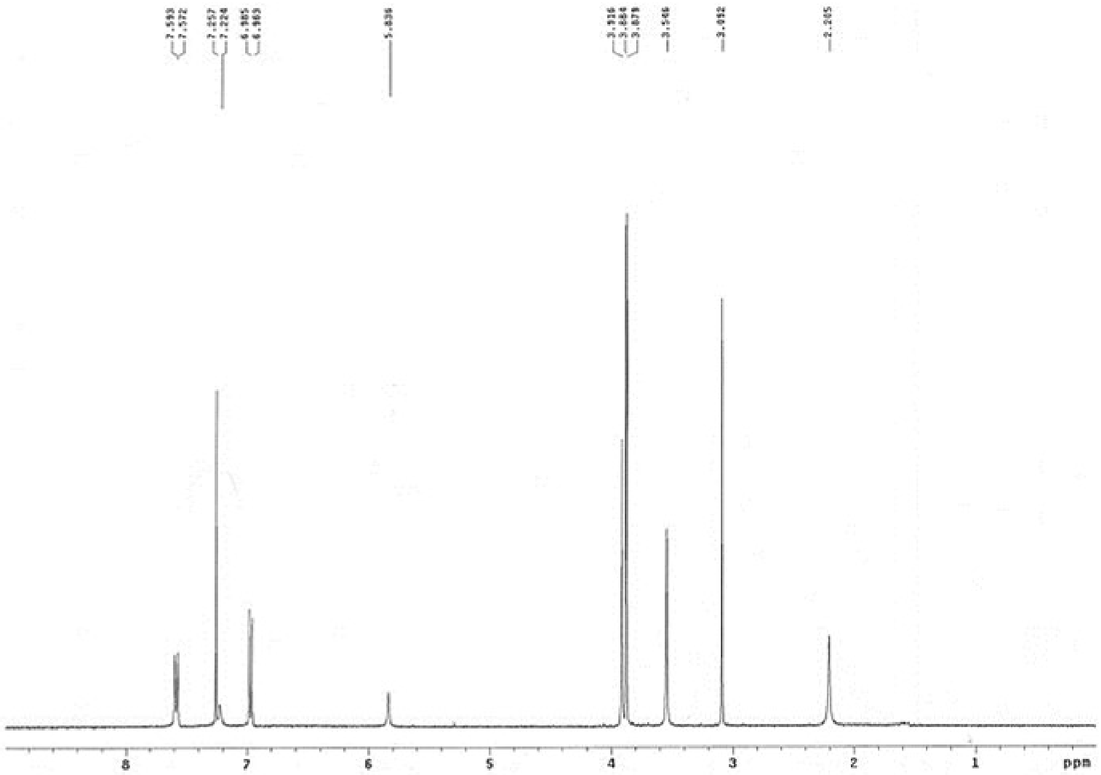

Fig. 18a: Compound **16** ^{1}H NMR spectra.

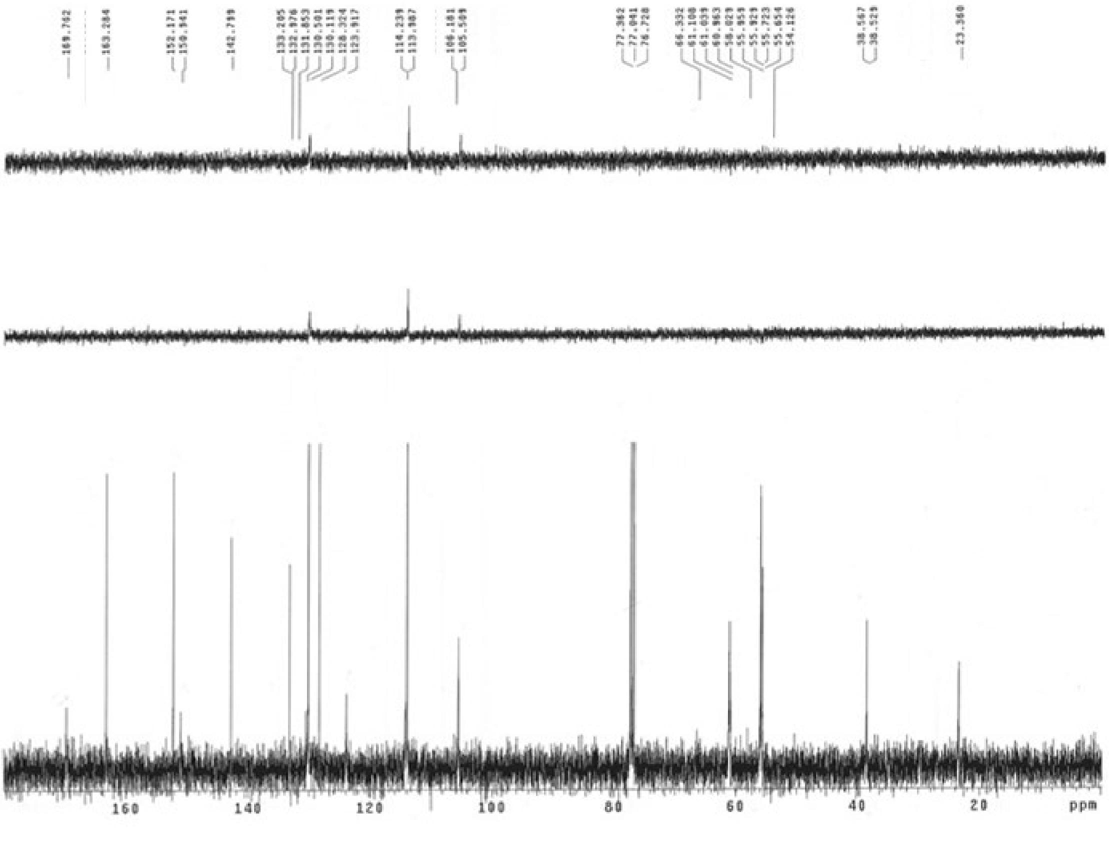

Fig. 18b: Compound **16** ^{13}C NMR spectra.

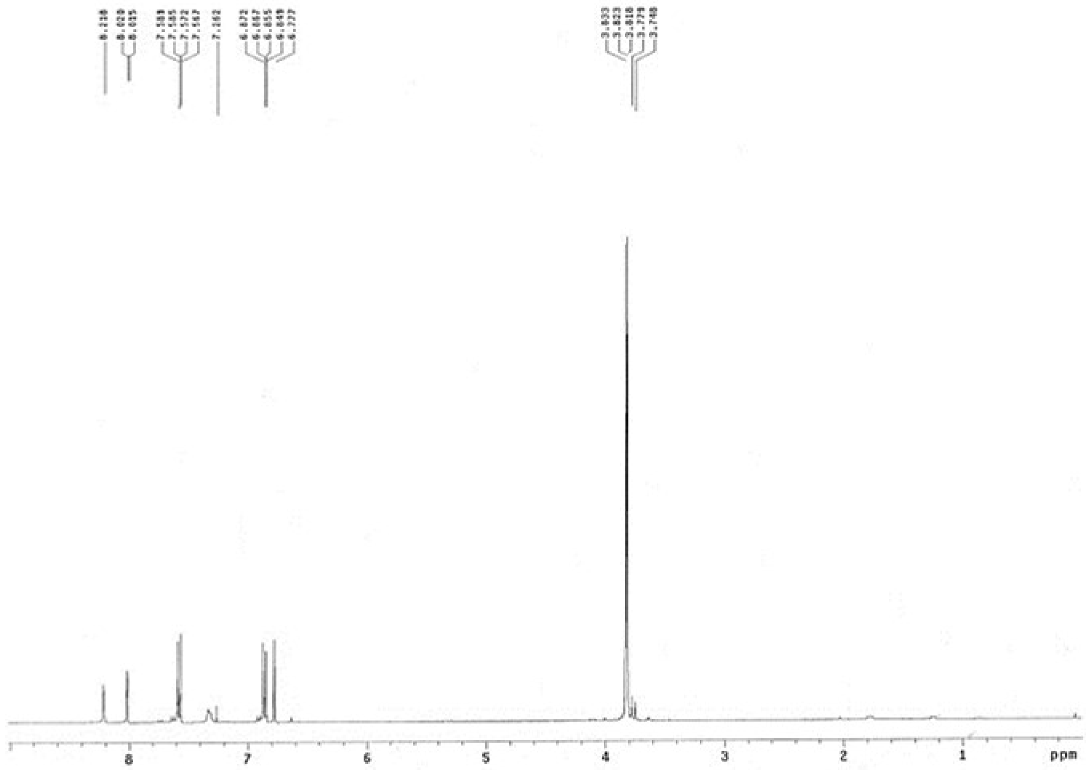

Fig. 19a: Compound **17** ^{1}H NMR spectra.

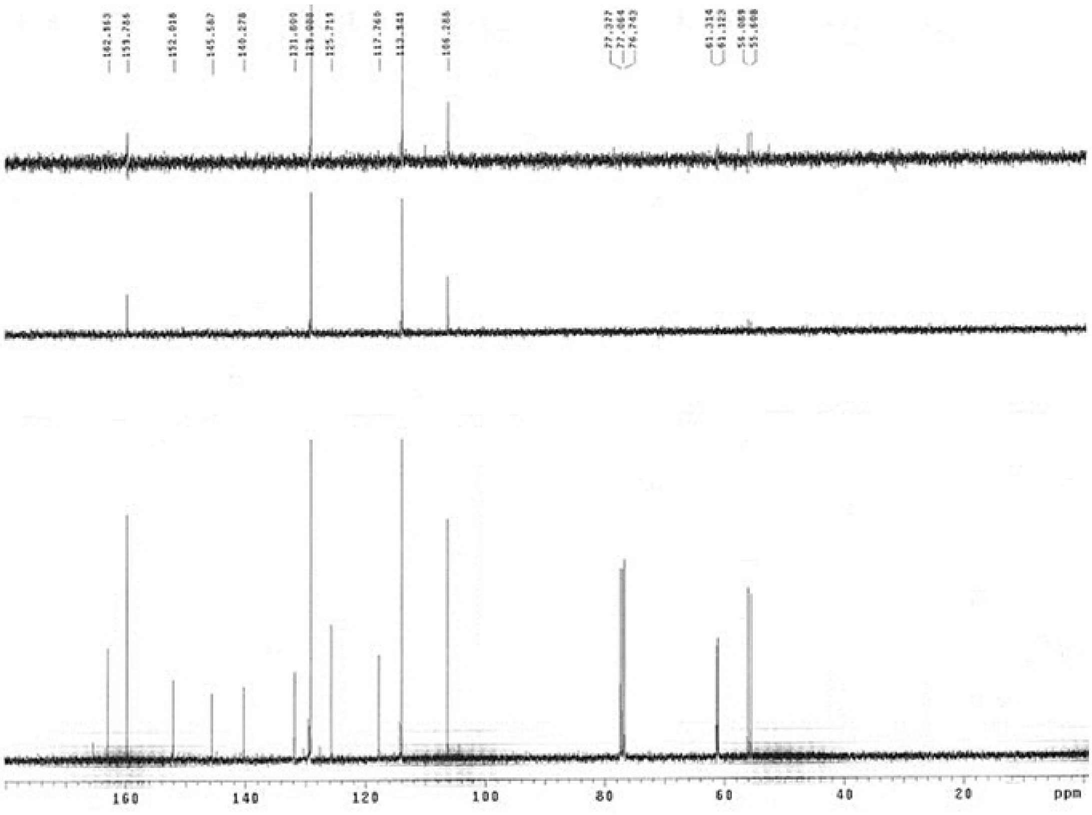

Fig. 19b: Compound **17** ^{13}C NMR spectra.

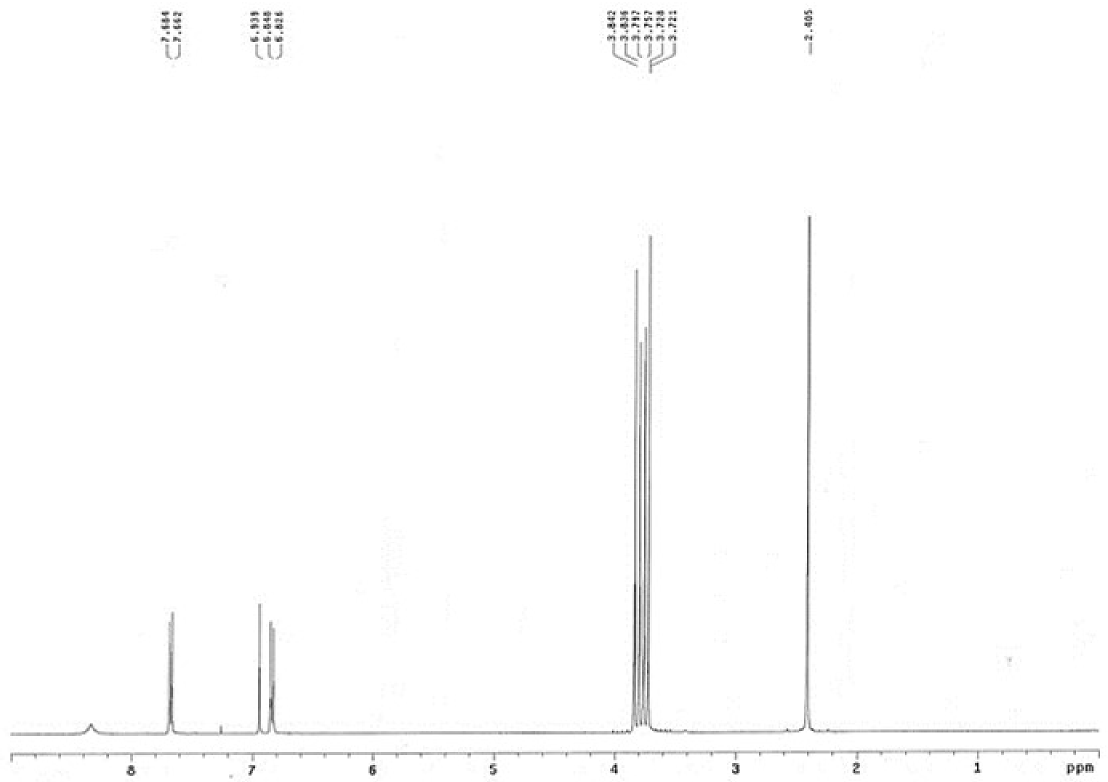

Fig. 20a: Compound **18** ^{1}H NMR spectra.

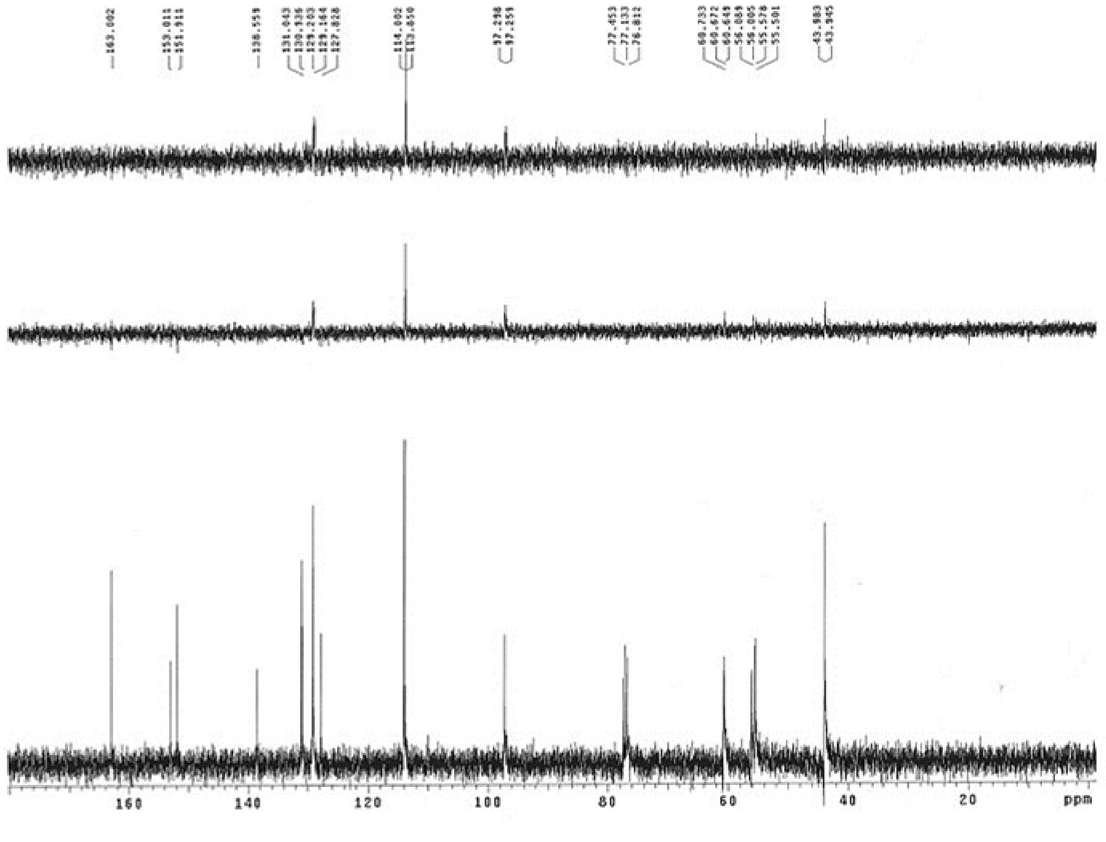

Fig. 20b: Compound **18** ^{13}C NMR spectra.

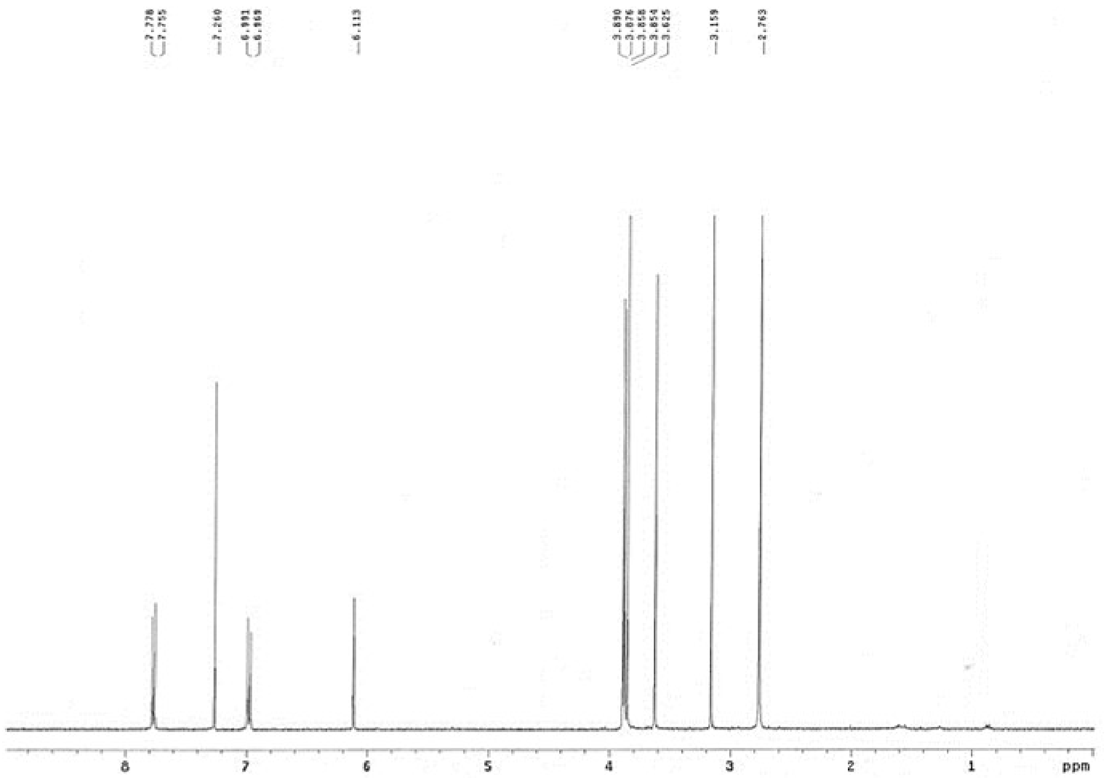

Fig. 21a: Compound **19** ^{1}H NMR spectra.

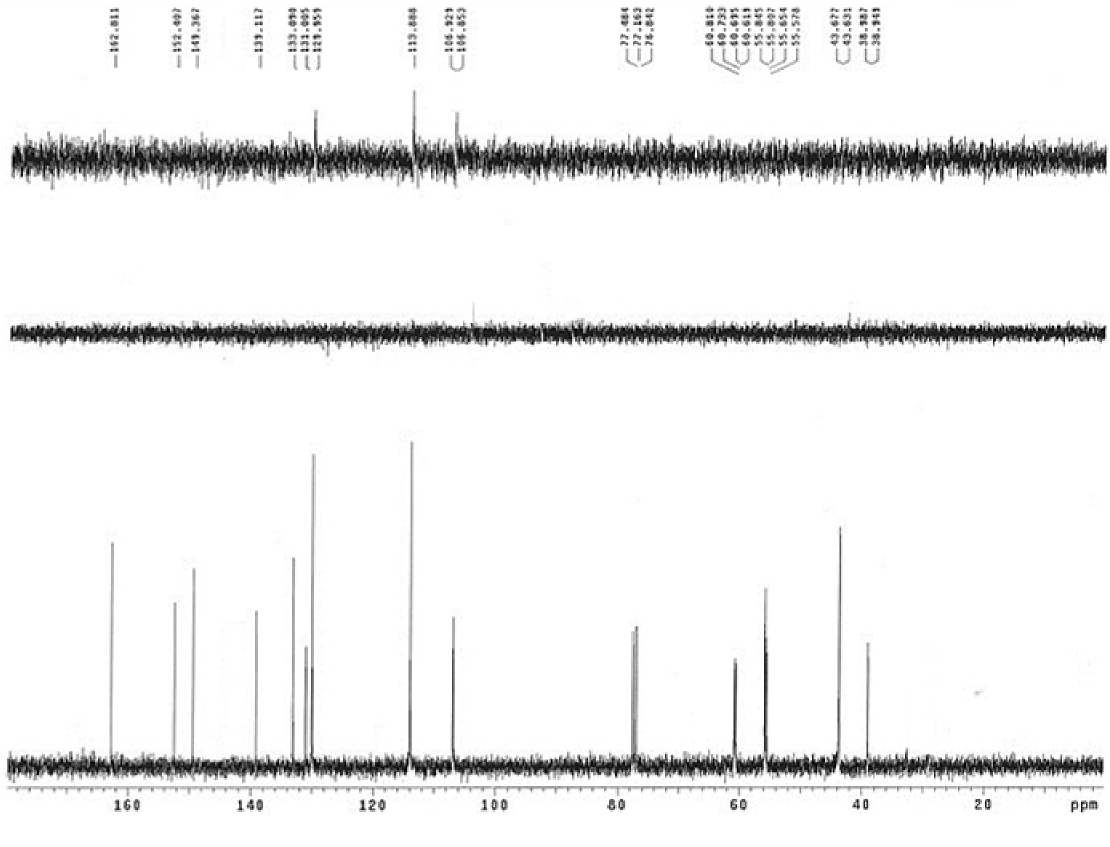

Fig. 21b: Compound **19** ^{13}C NMR spectra.

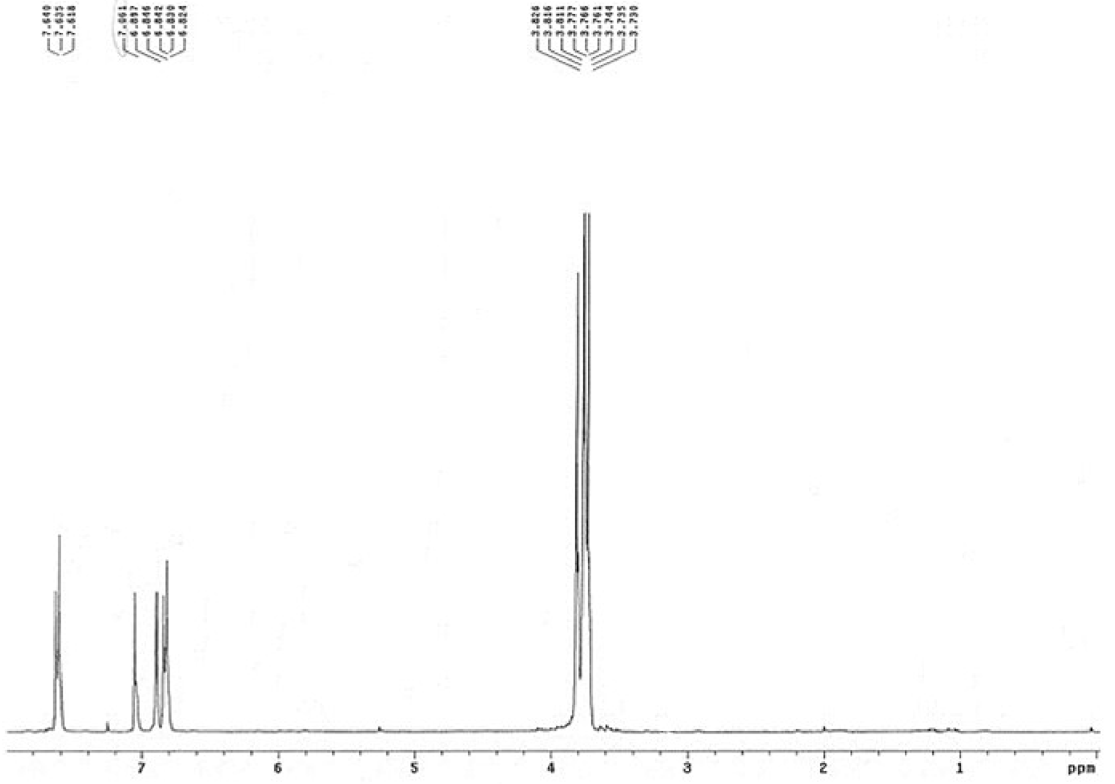

Fig. 22a: Compound **20** ^{1}H NMR spectra.

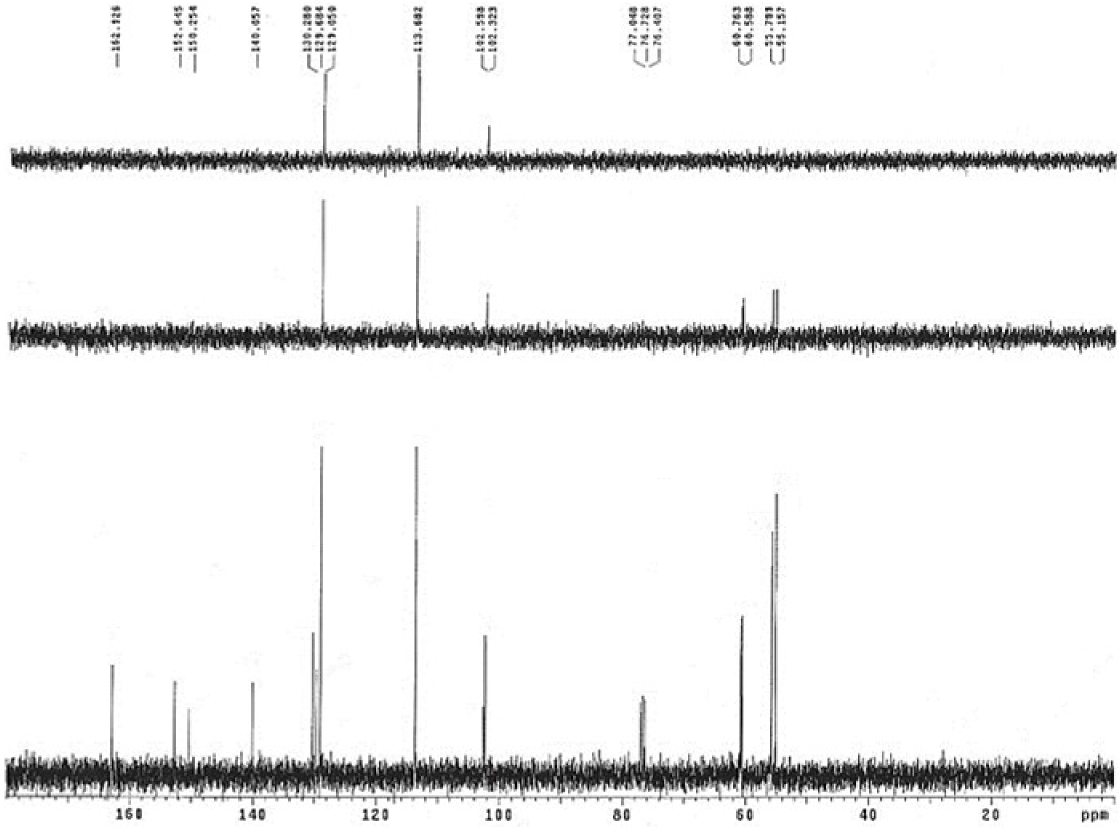

Fig. 22b: Compound **20** ^{13}C NMR spectra.

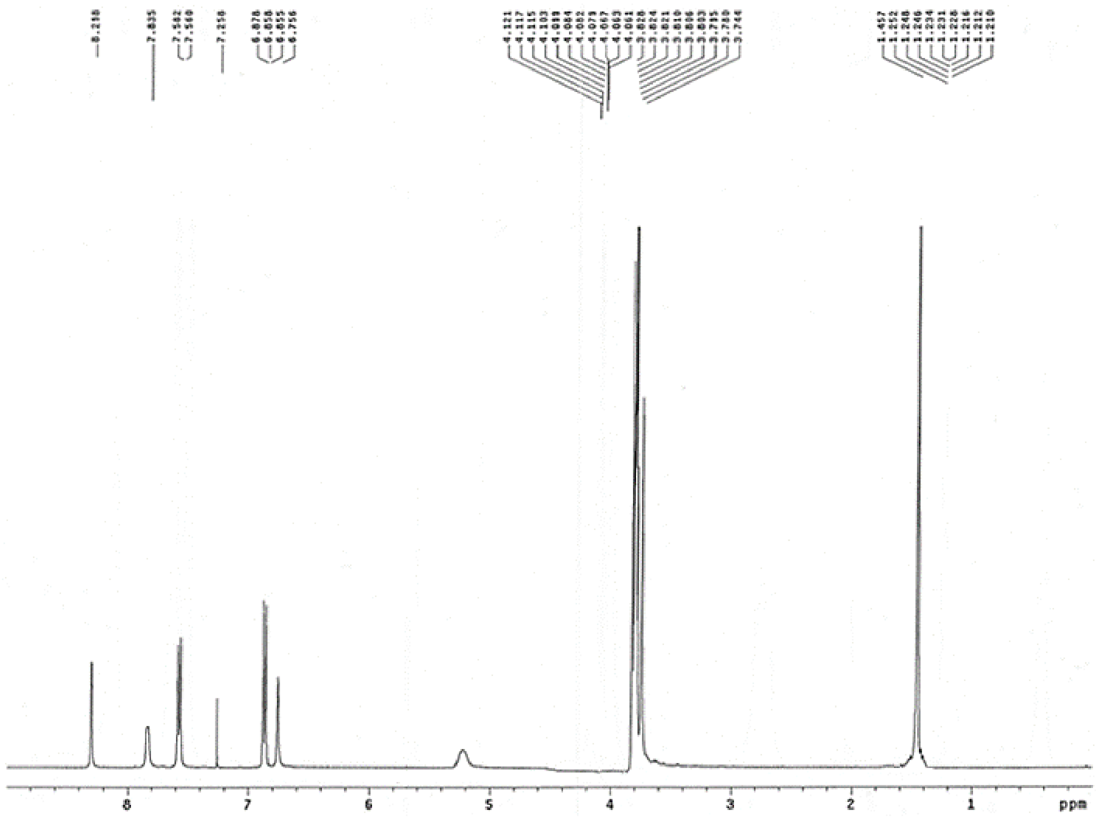

Fig. 23a: Compound **21** ^{1}H NMR spectra.

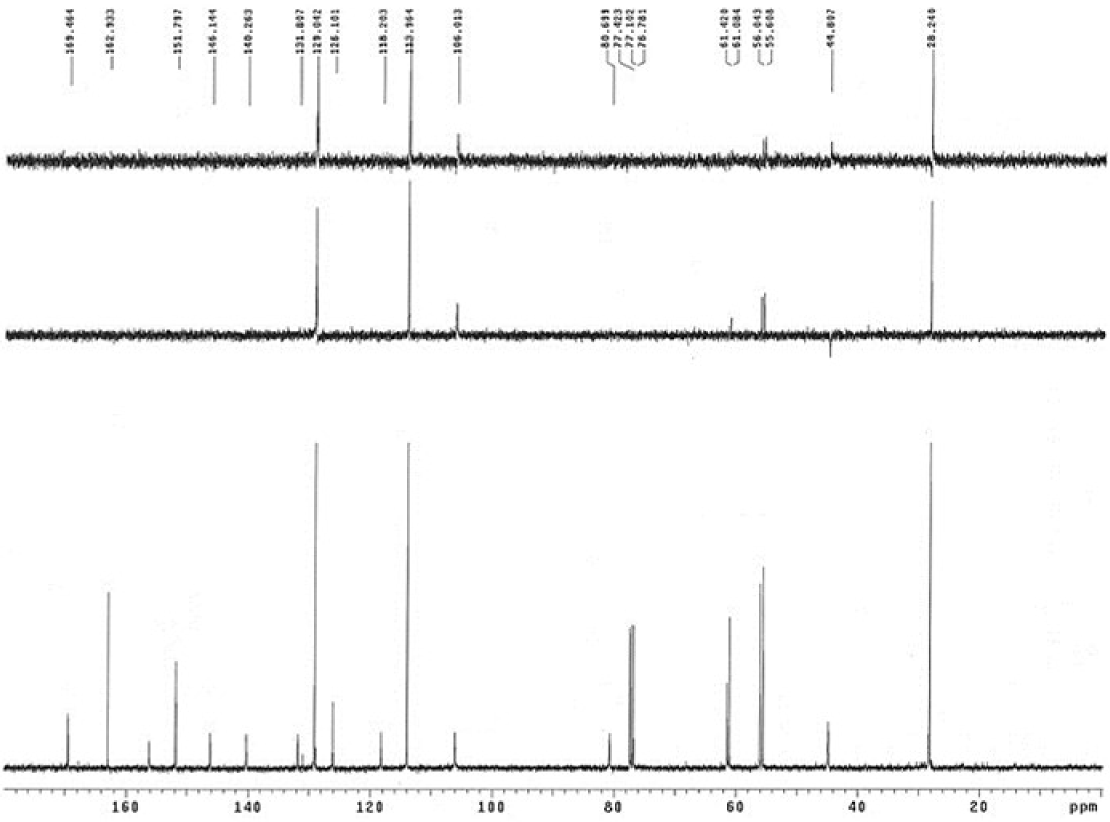

Fig. 23b: Compound **21** ^{13}C NMR spectra.

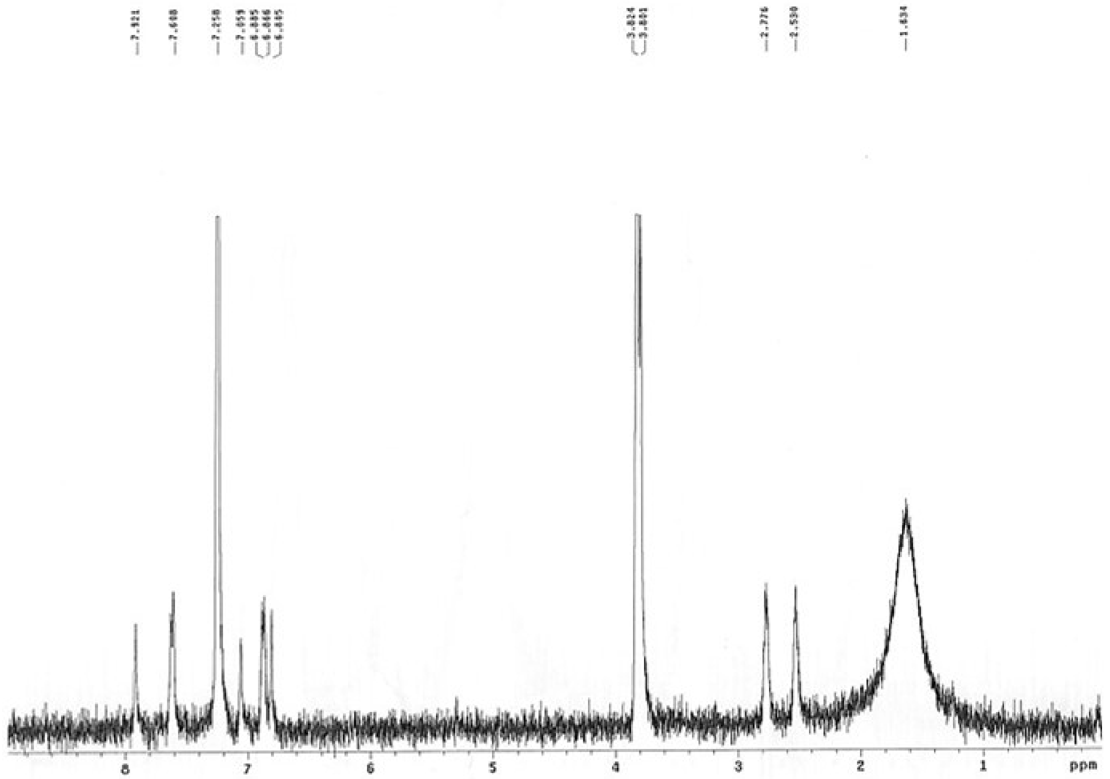

Fig. 24a: Compound **22** ^{1}H NMR spectra.

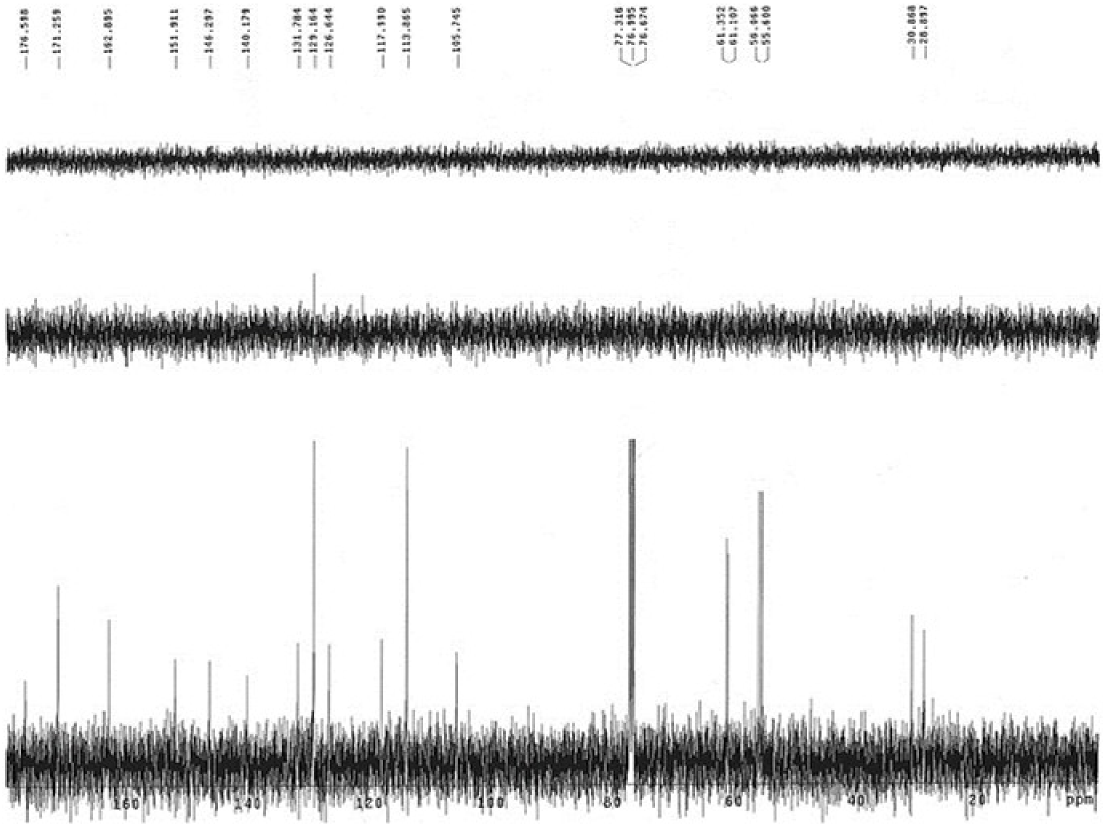

Fig. 24b: Compound **22** ^{13}C NMR spectra.

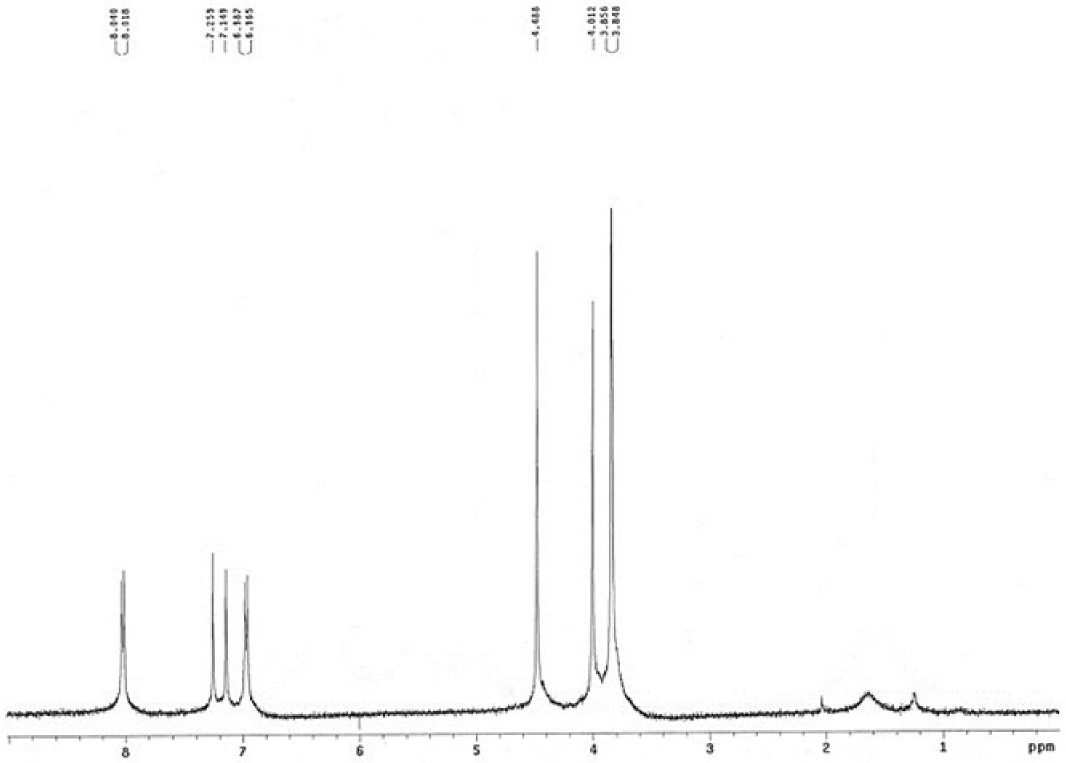

Fig. 25a: Compound **23** ^{1}H NMR spectra.

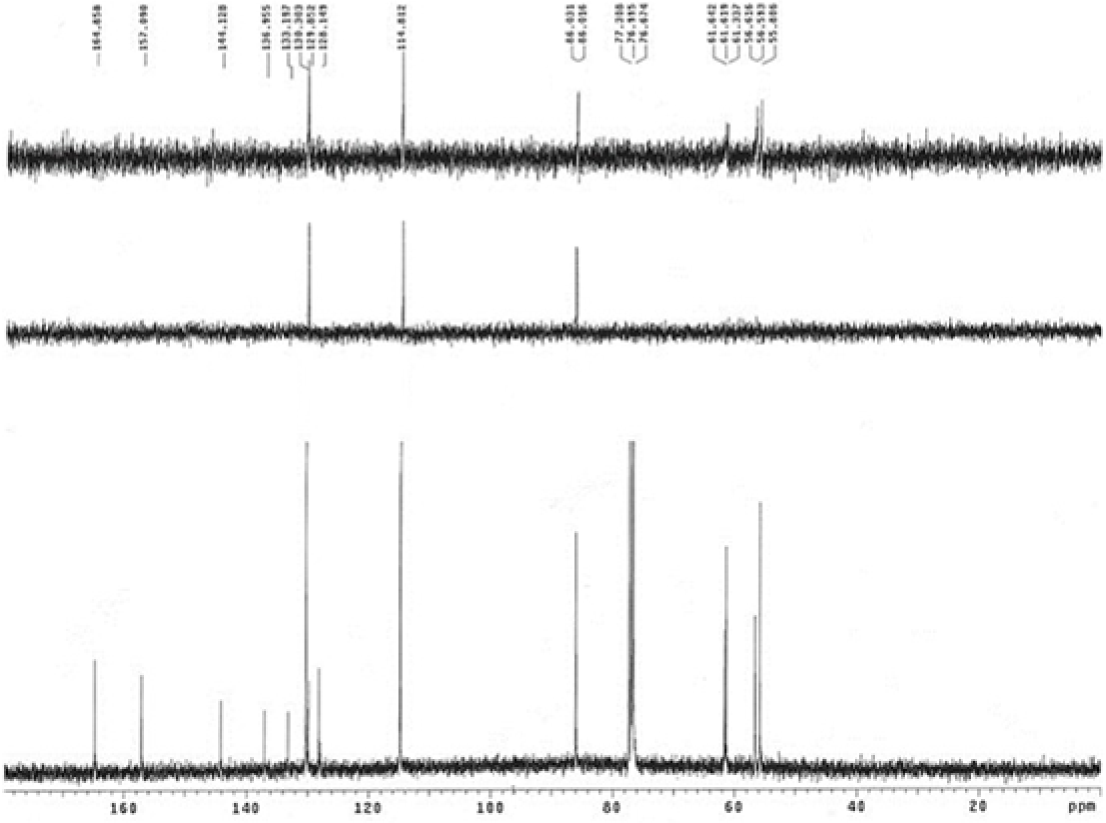

Fig. 25b: Compound **23** ^{13}C NMR spectra.

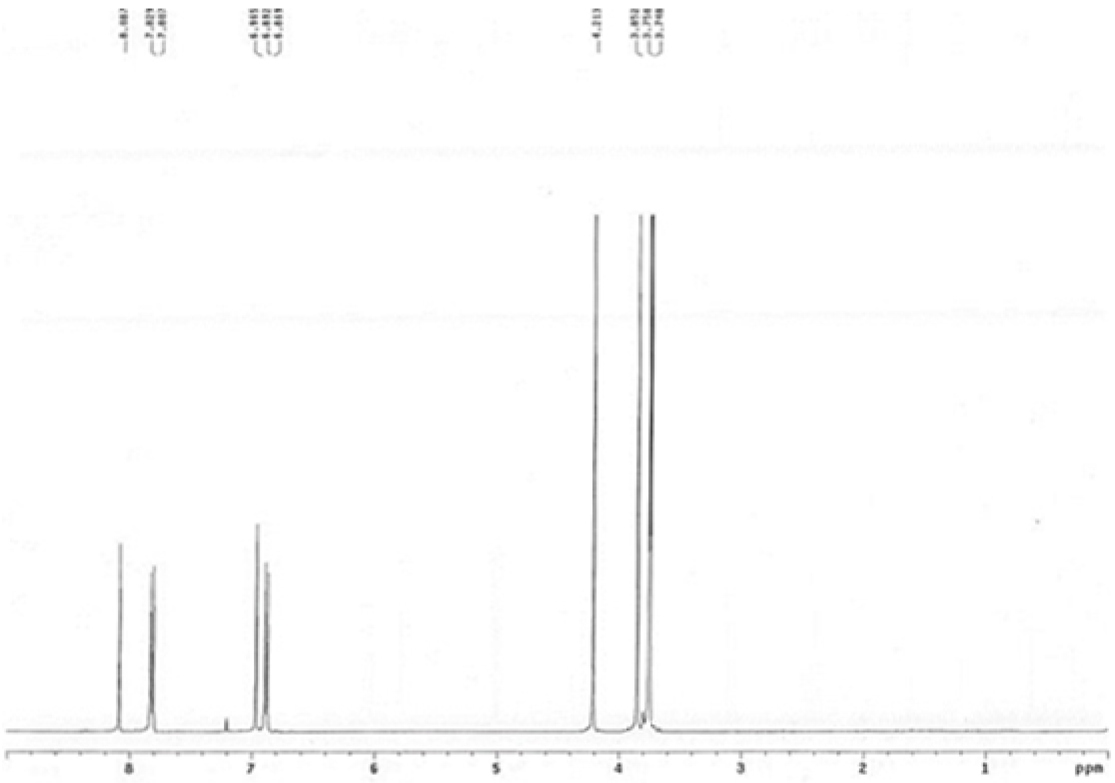

Fig. 26a: Compound **24** ^{1}H NMR spectra.

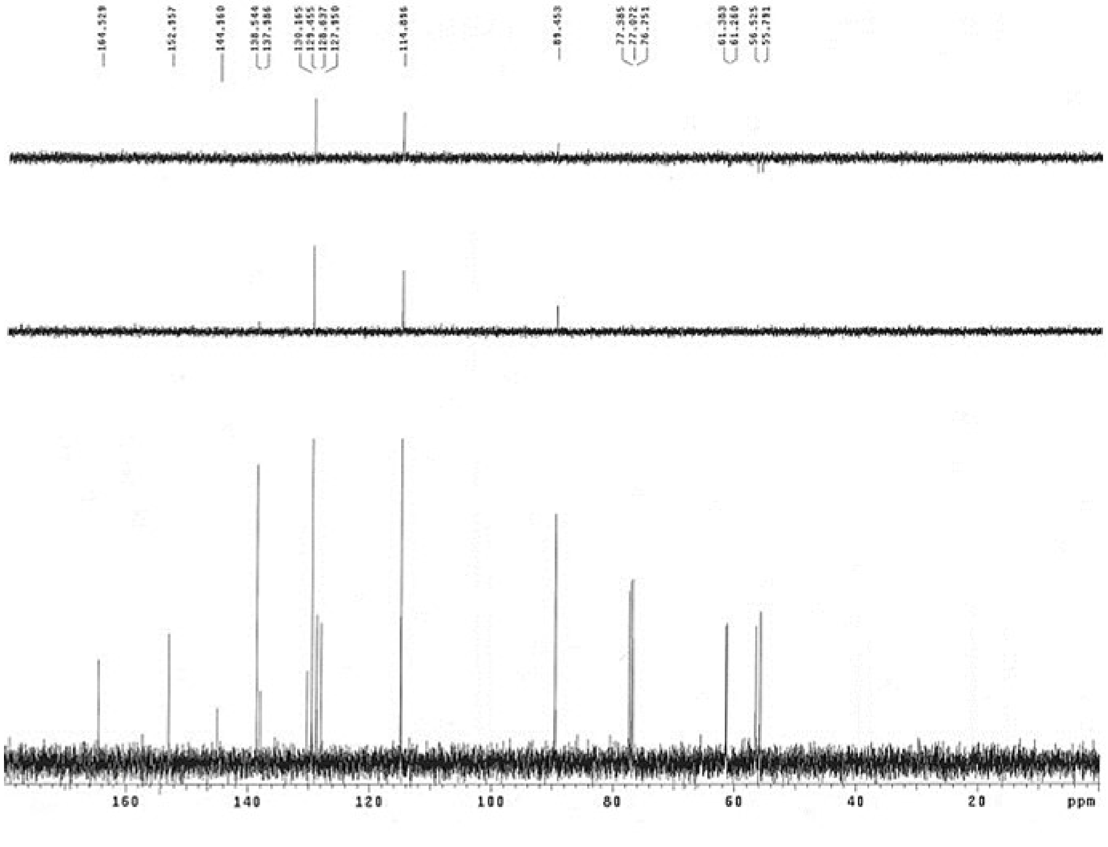

Fig. 26b: Compound **24** ^{13}C NMR spectra.

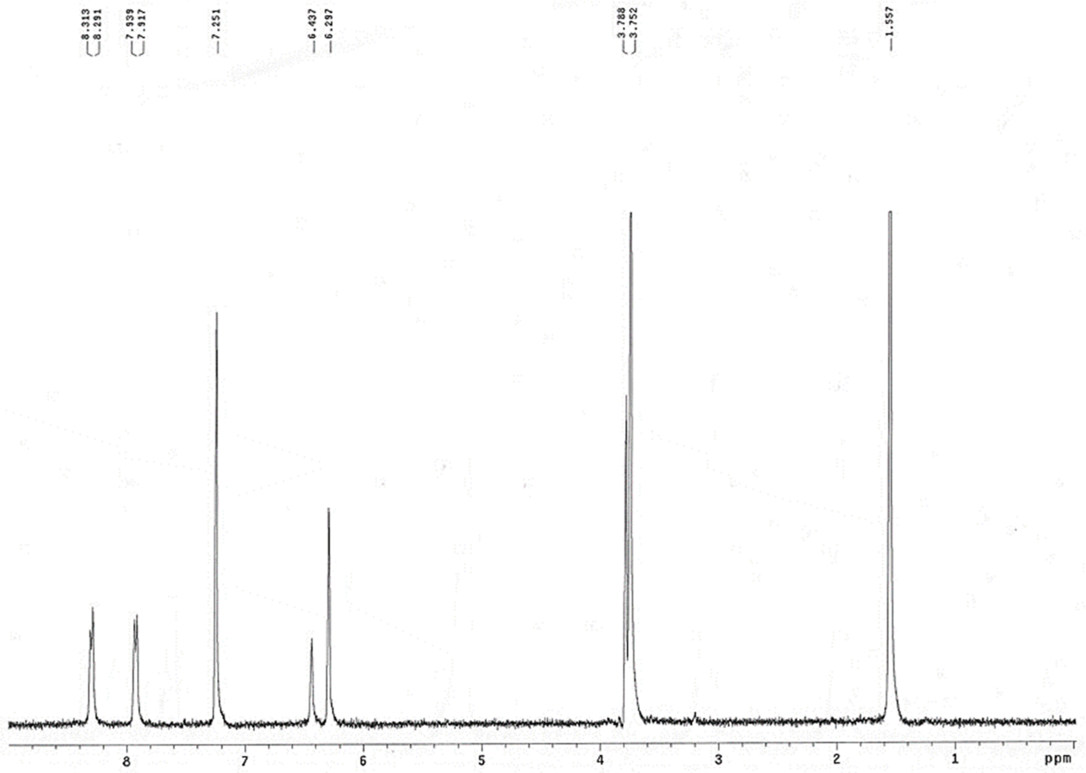

Fig. 27: Compound **25** ^{1}H NMR spectra.

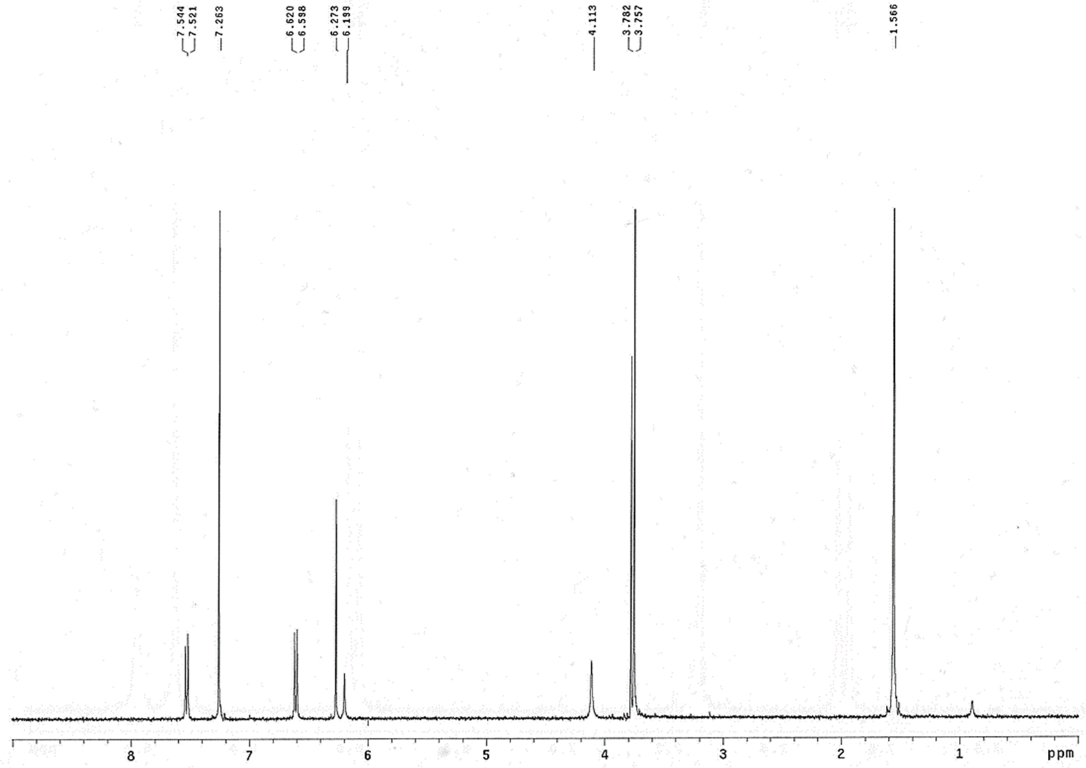

Fig. 28: Compound **26** ^{13}C NMR spectra.

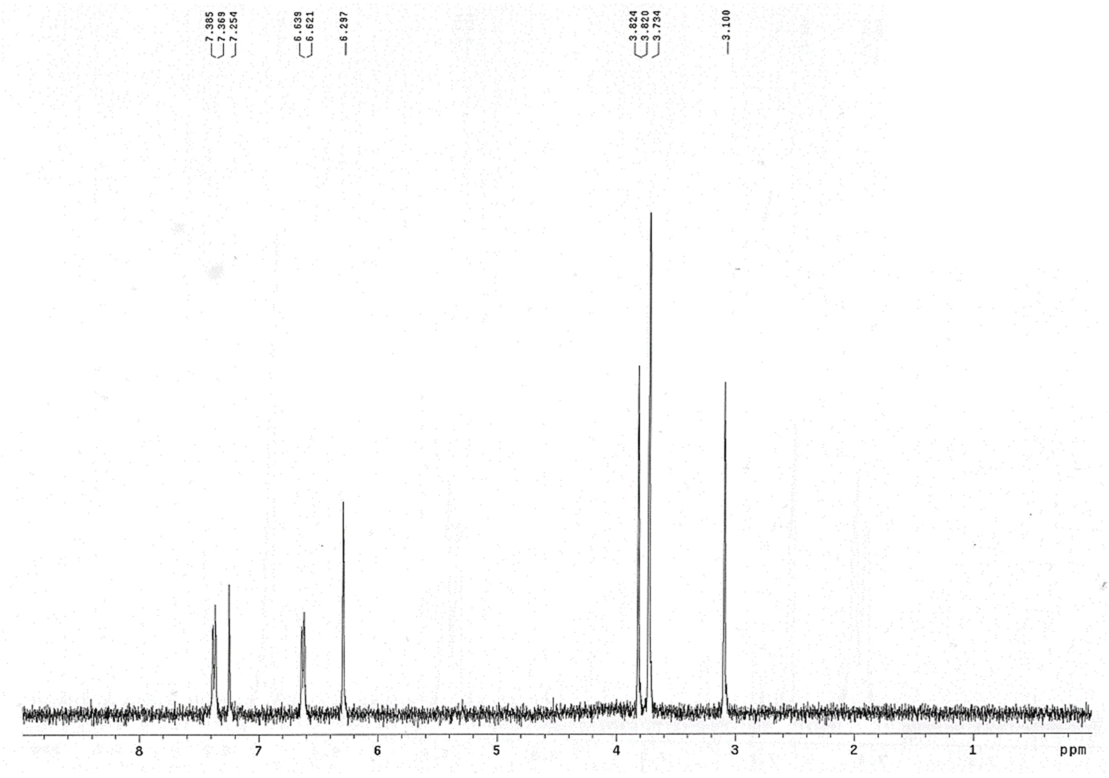

Fig. 29a: Compound **27** ^{1}H NMR spectra.

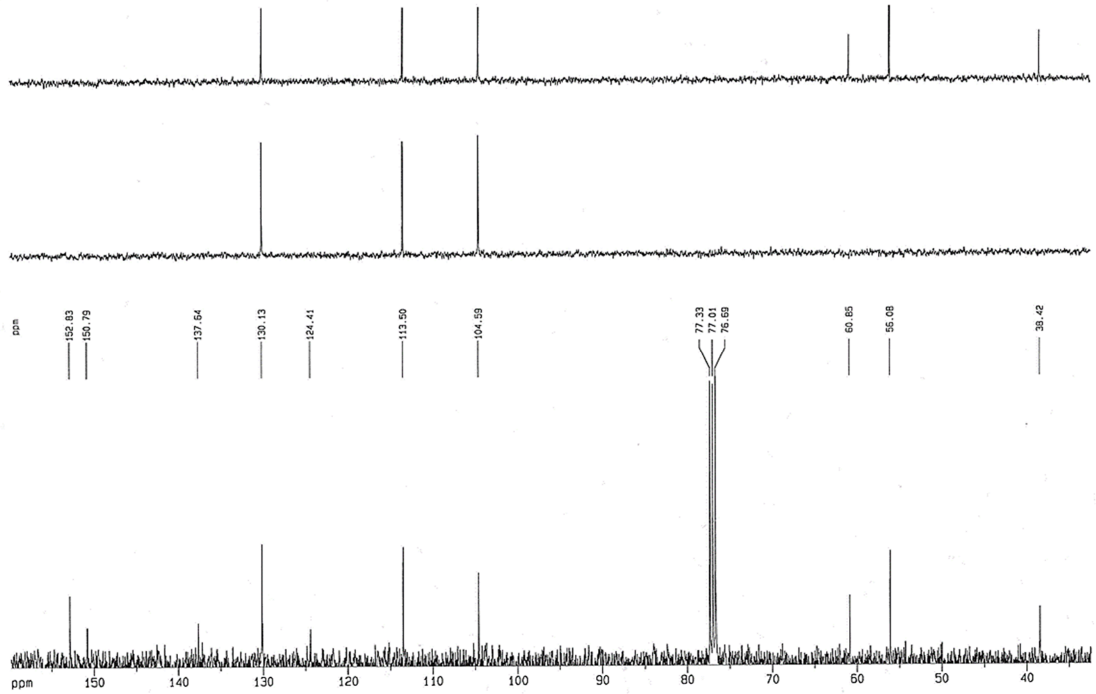

Fig. 29b: Compound **27** ^{13}C NMR spectra.

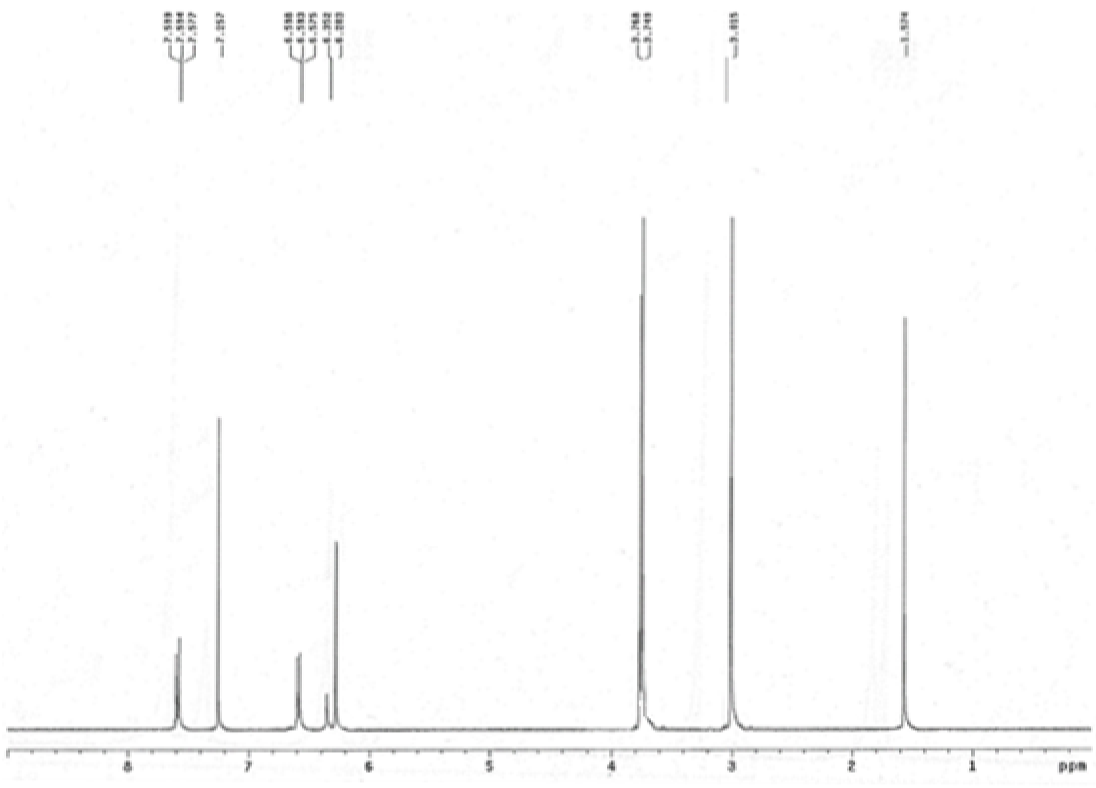

Fig. 30a: Compound **28** ^{1}H NMR spectra.

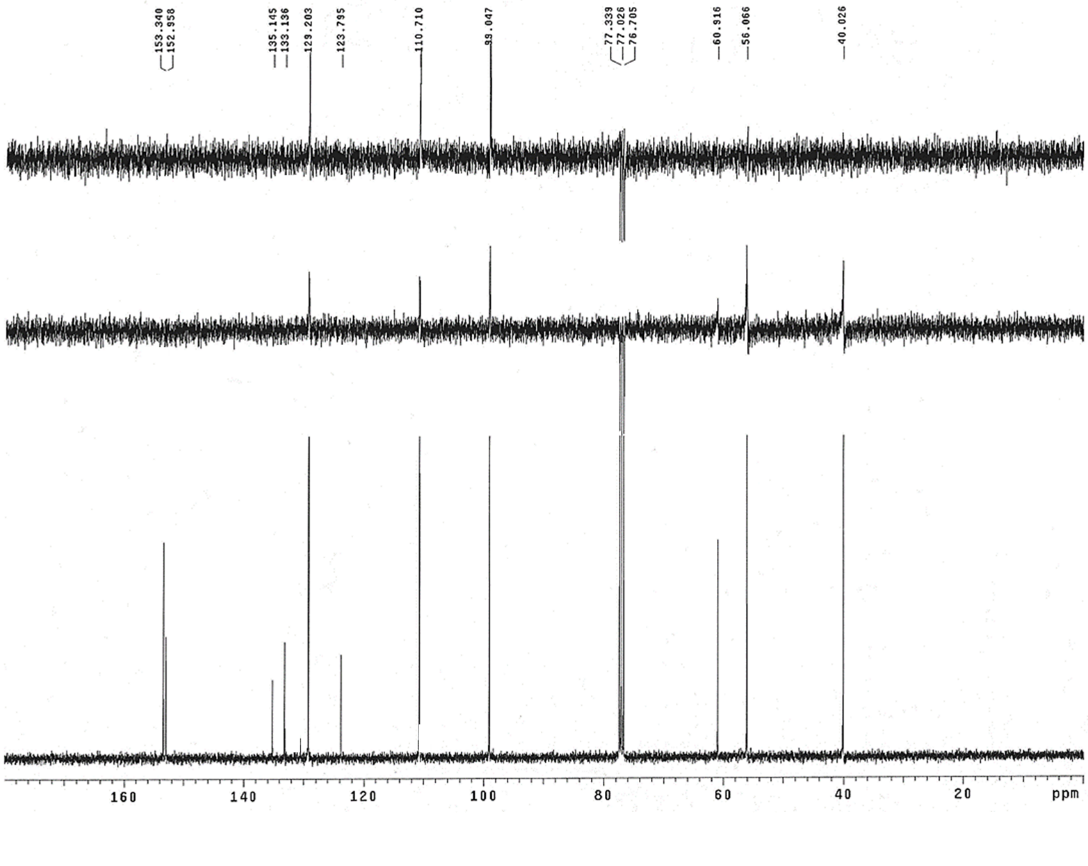

Fig. 30b: Compound **28** ^{13}C NMR spectra.

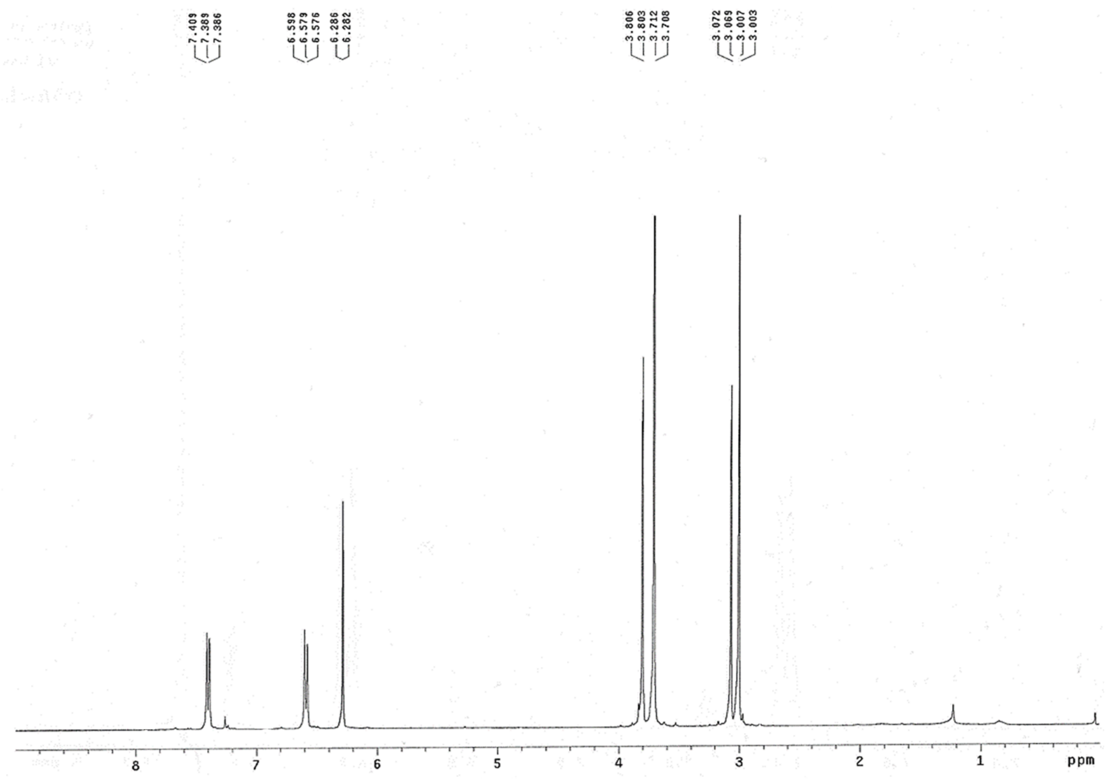

Fig. 31a: Compound **29a** ^{1}H NMR spectra.

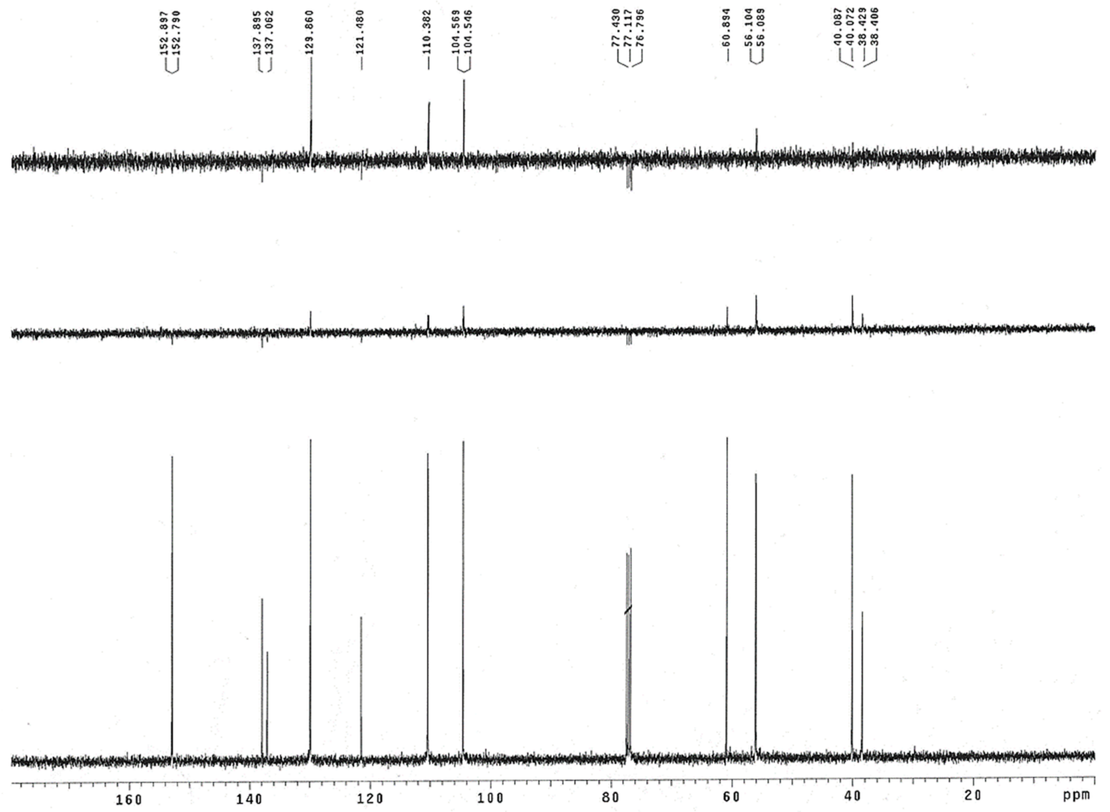

Fig. 31b: Compound **29a** ^{13}C NMR spectra.

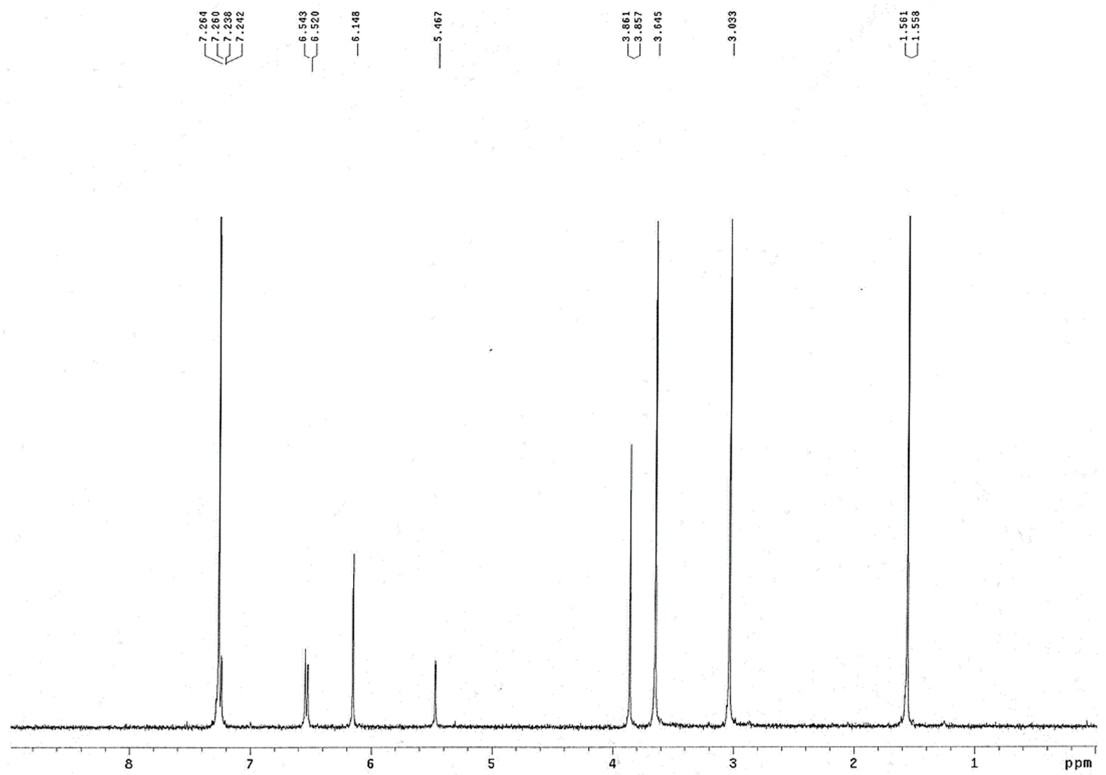

Fig. 32: Compound **29b** ^{1}H NMR spectra.

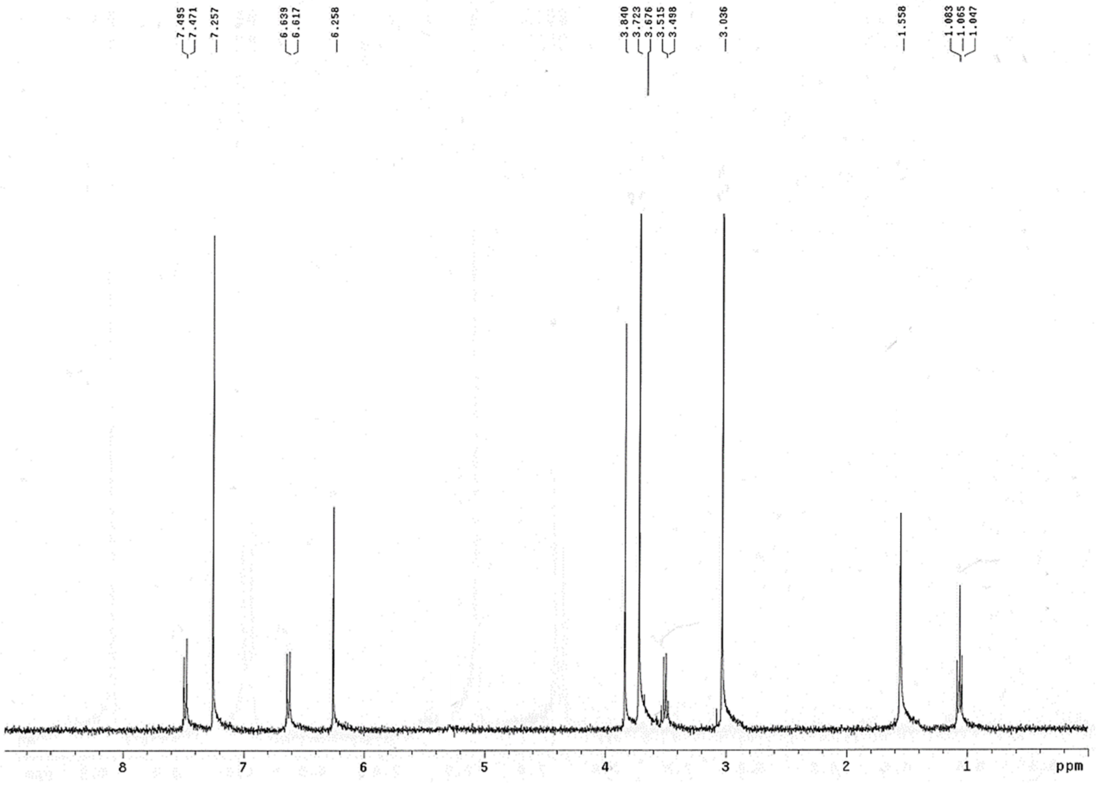

Fig. 33a: Compound **30** ^{1}H NMR spectra.

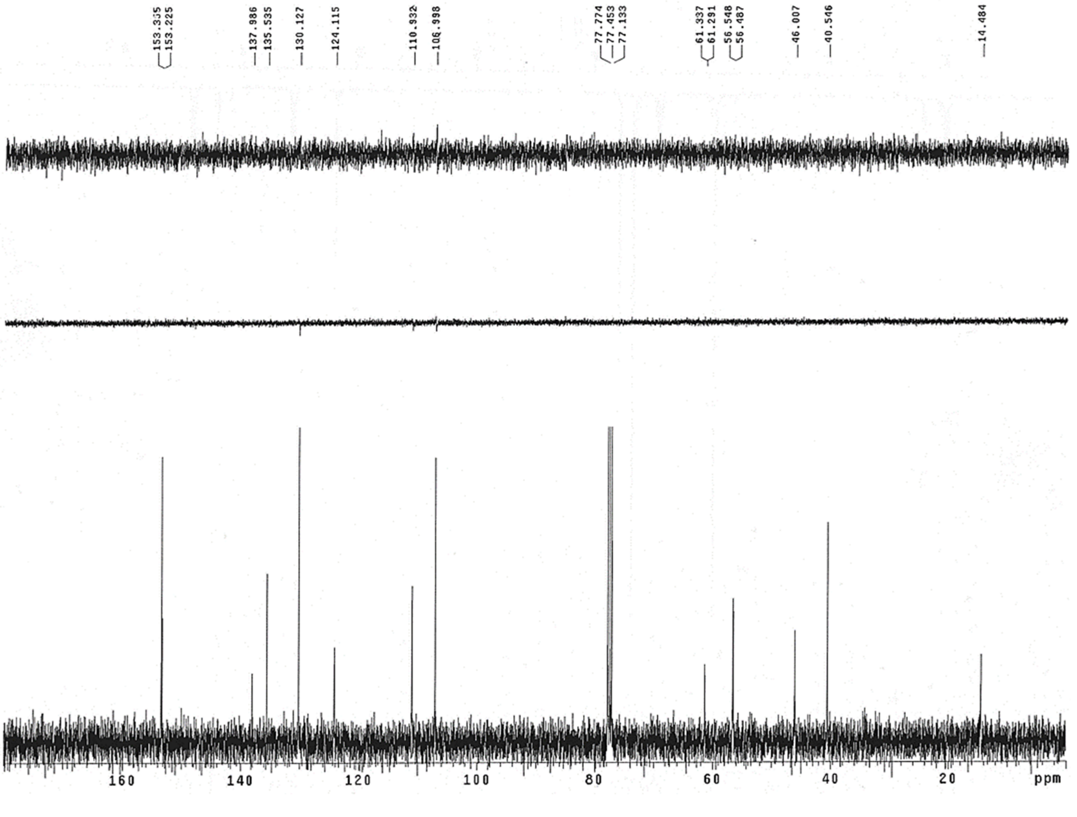

Fig. 33b: Compound **30** ^{13}C NMR spectra.

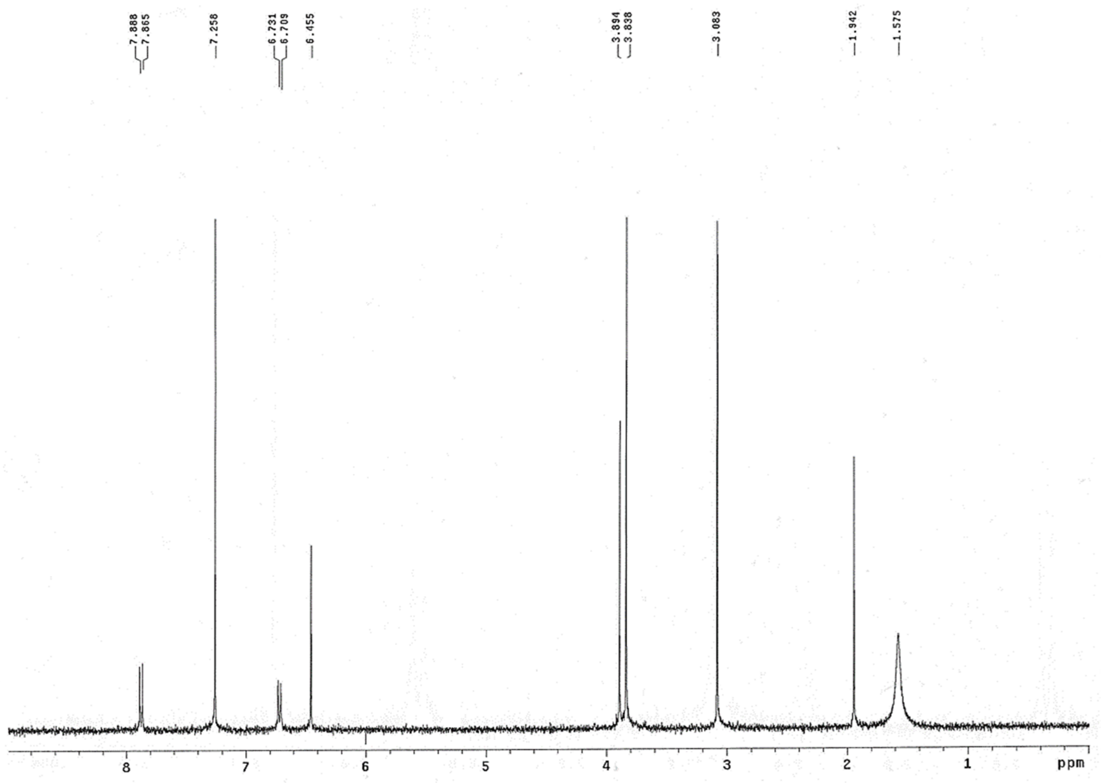

Fig. 34a: Compound **31** ^{1}H NMR spectra.

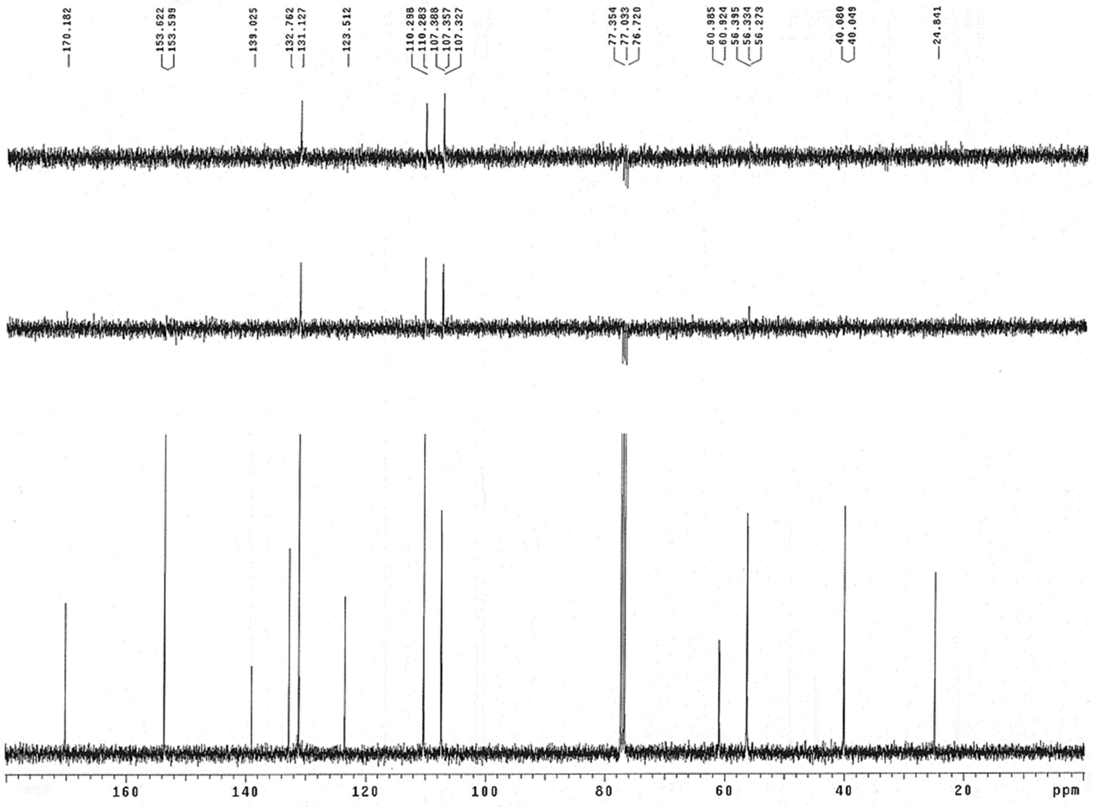

Fig. 34b: Compound **31** ^{13}C NMR spectra.

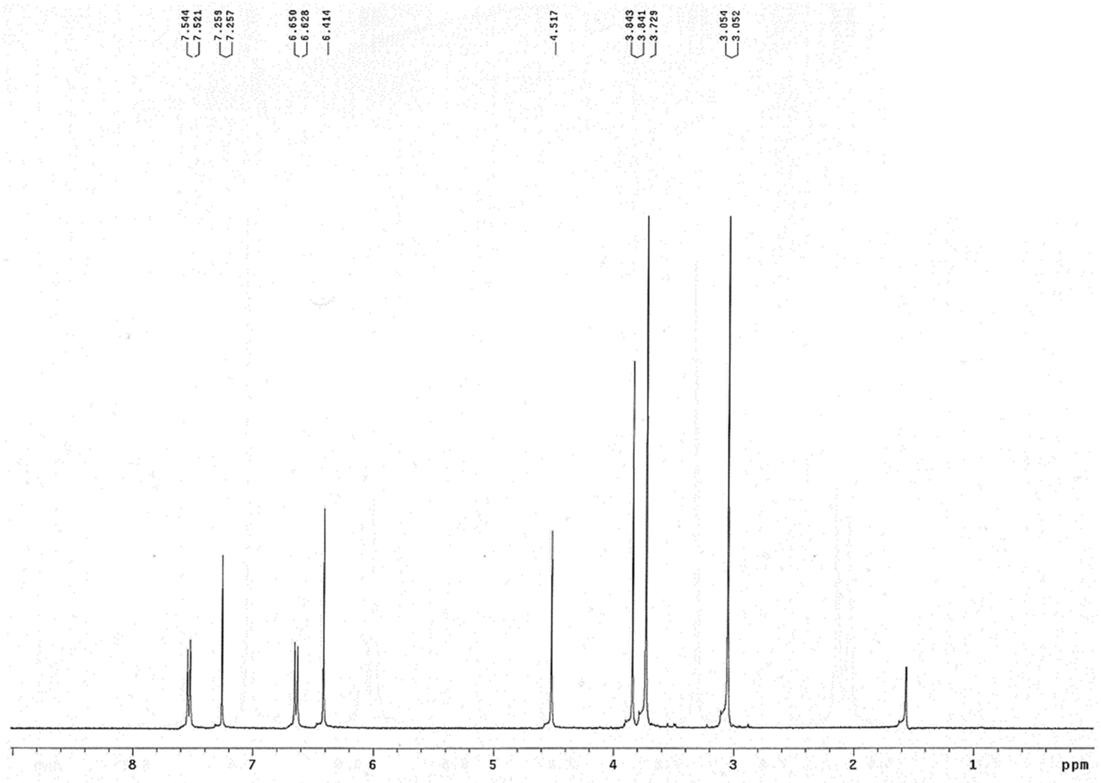

Fig. 35a: Compound **32** ^{1}H NMR spectra.

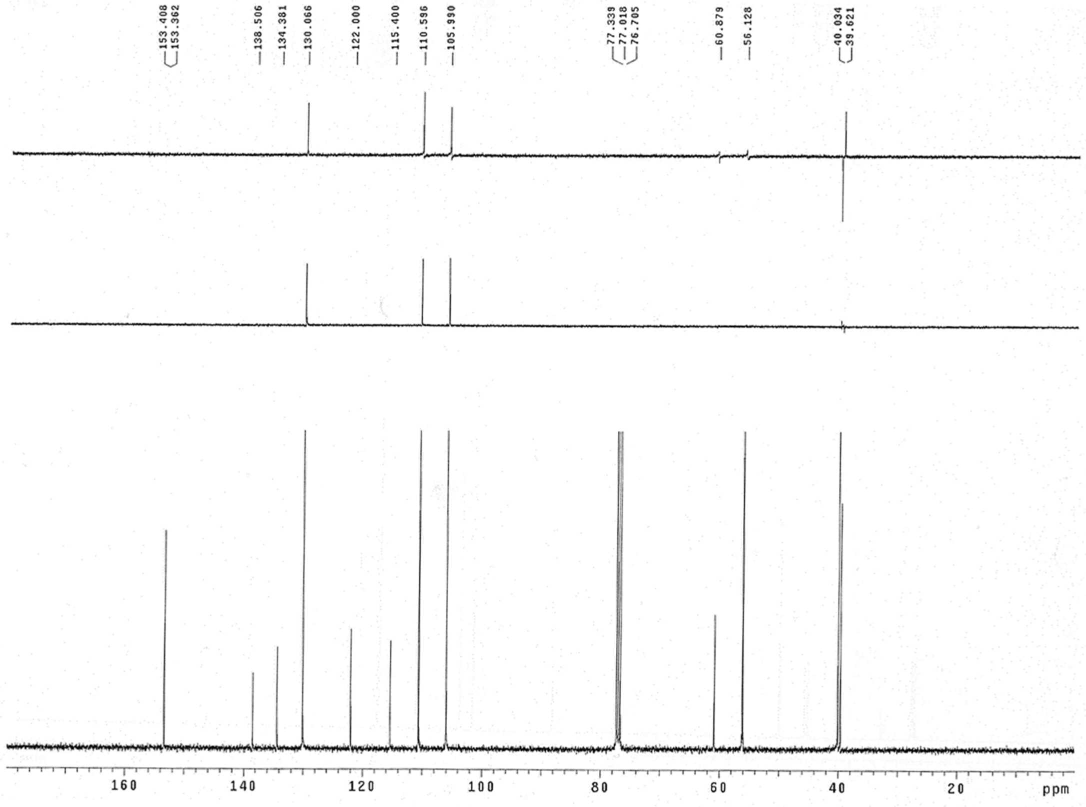

Fig. 35b: Compound **32** ^{13}C NMR spectra.

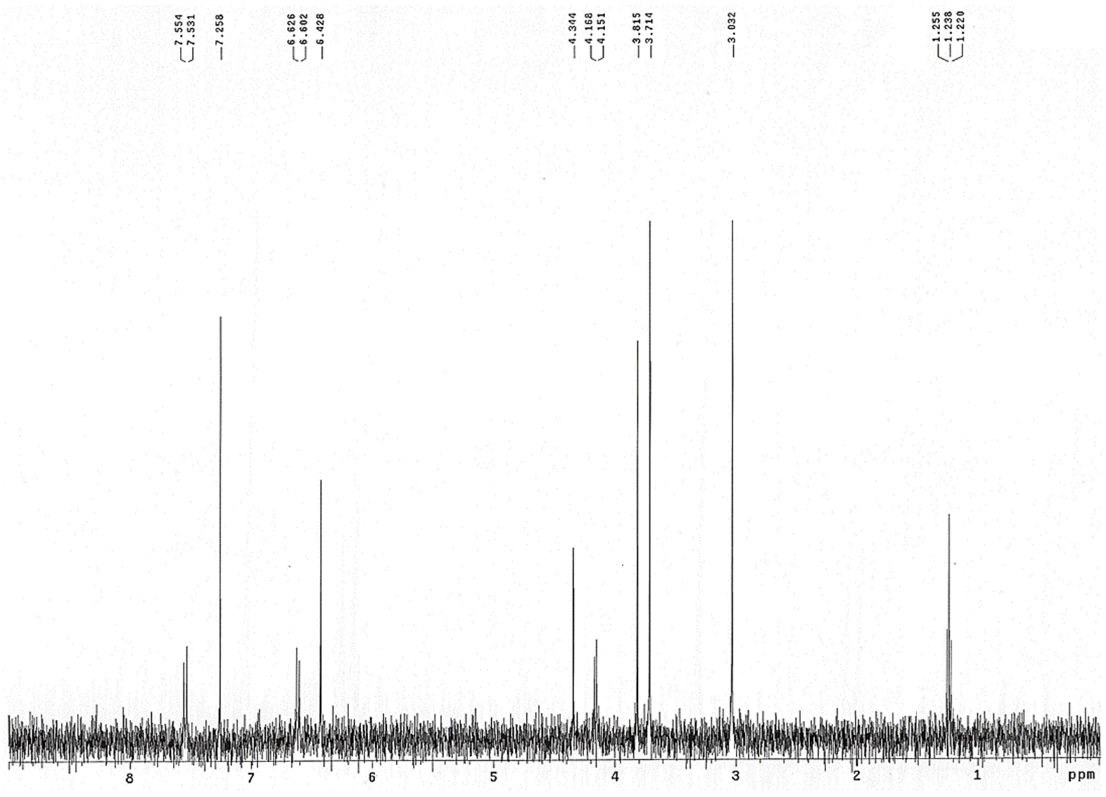

Fig. 36a: Compound **33** ^{1}H NMR spectra.

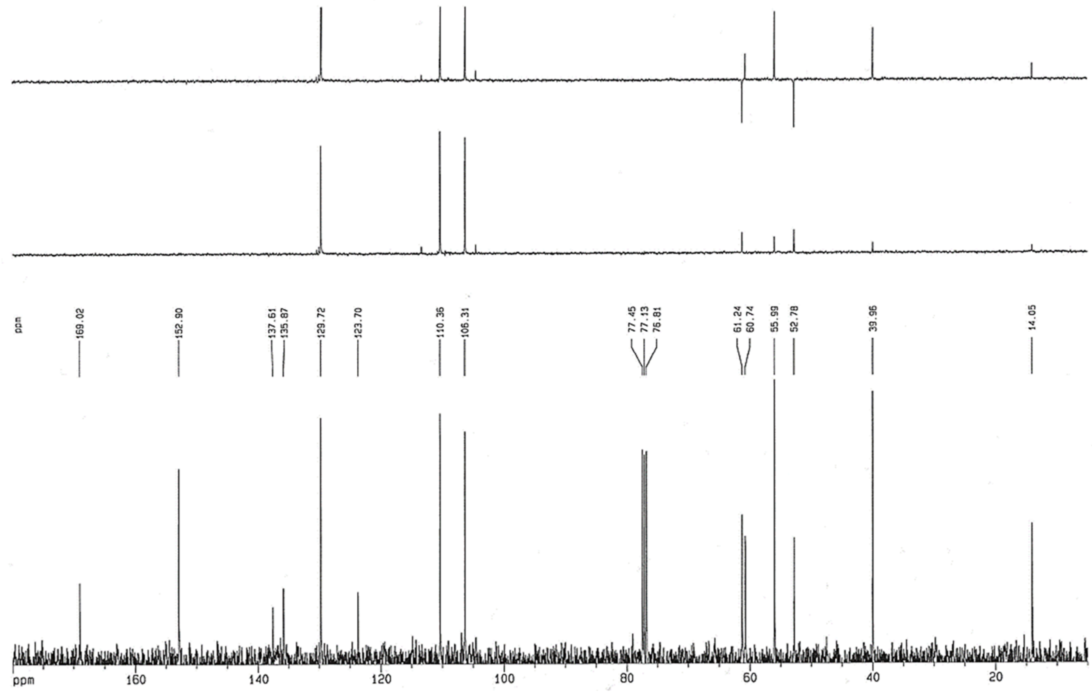

Fig. 36b: Compound **33** ^{13}C NMR spectra.

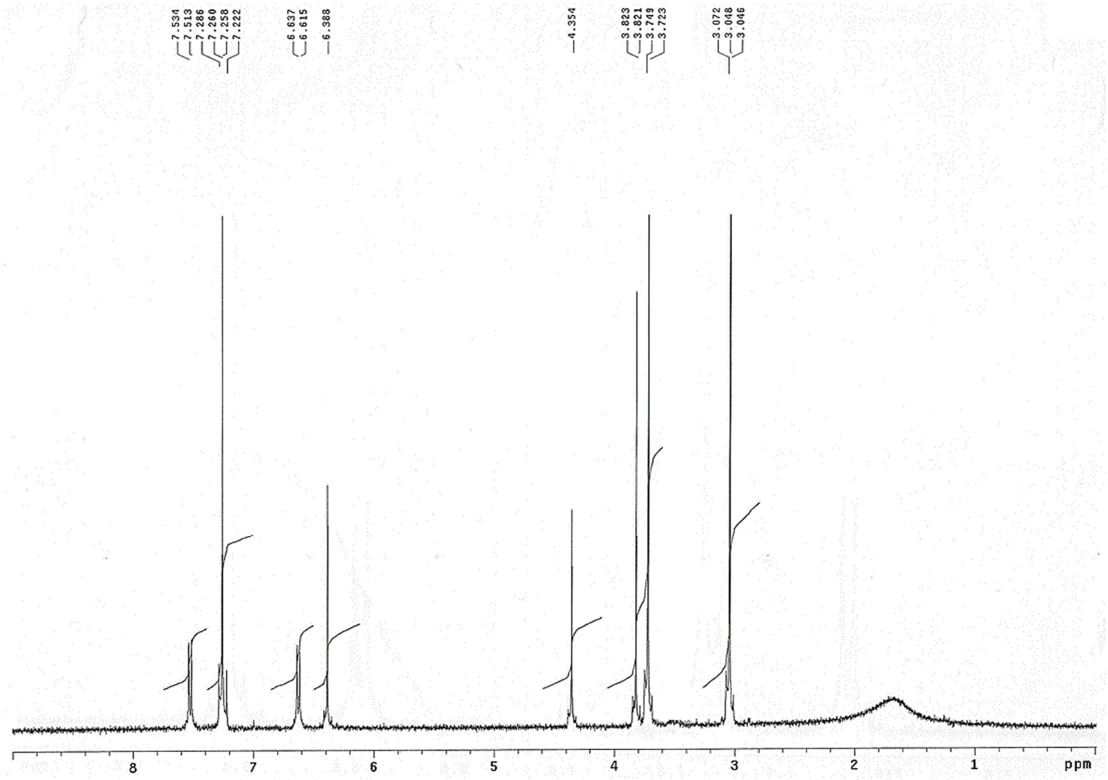

Fig. 37a: Compound **34** ^{1}H NMR spectra.

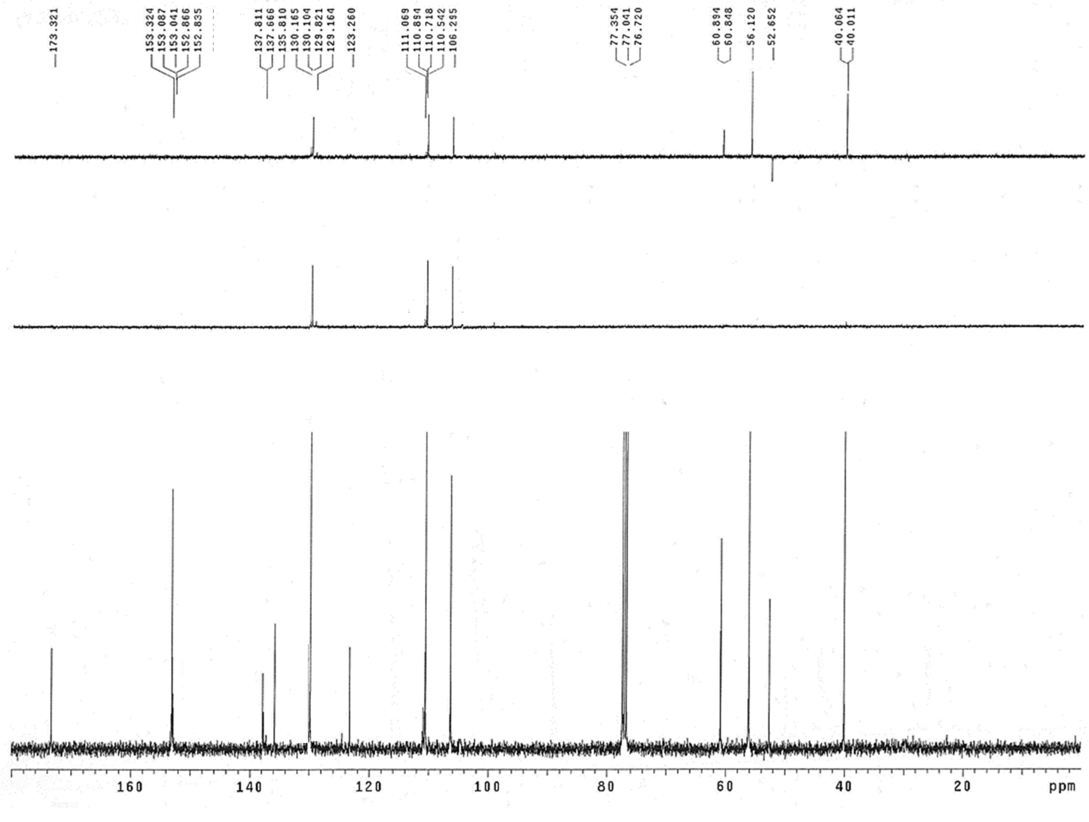

Fig. 37b: Compound **34** ^{13}C NMR spectra.

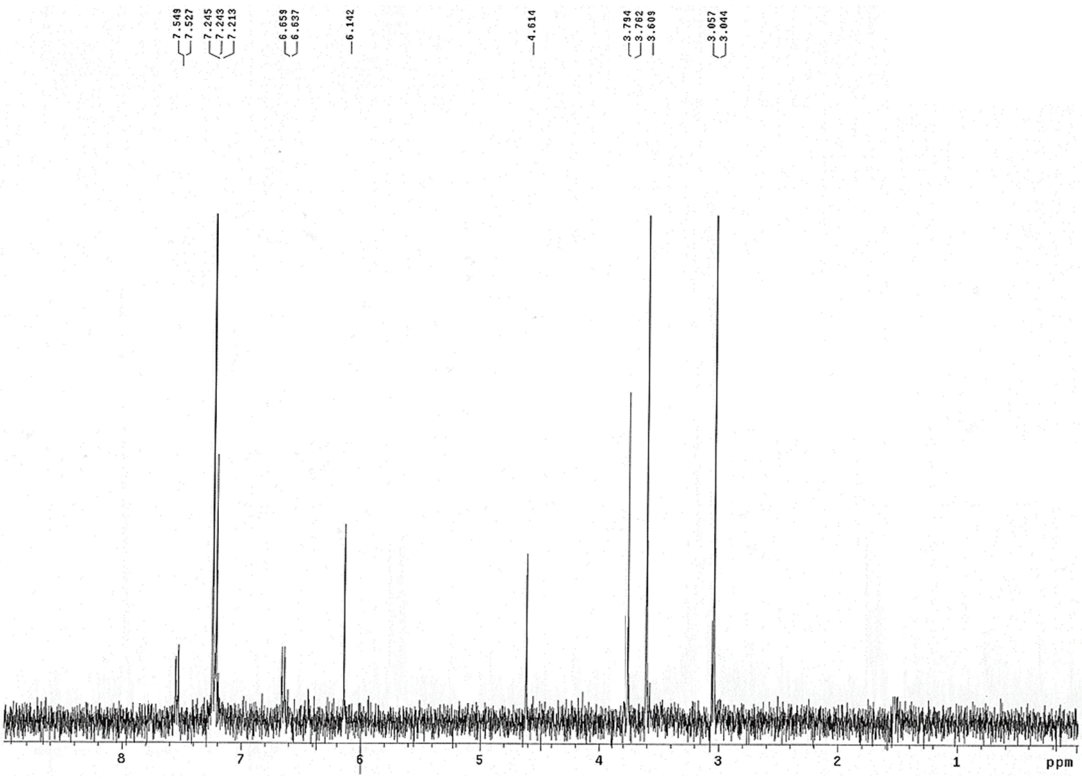

Fig. 38a: Compound **35a** ^{1}H NMR spectra.

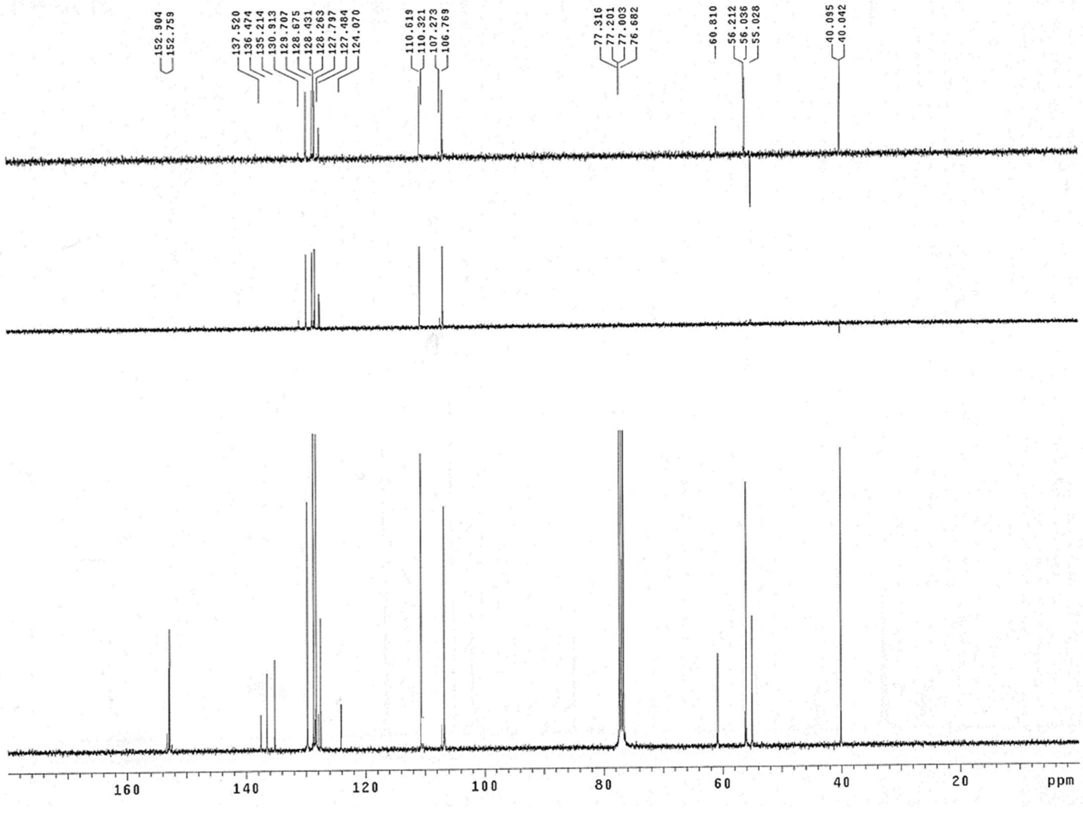

Fig. 38b: Compound **35a** ^{13}C NMR spectra.

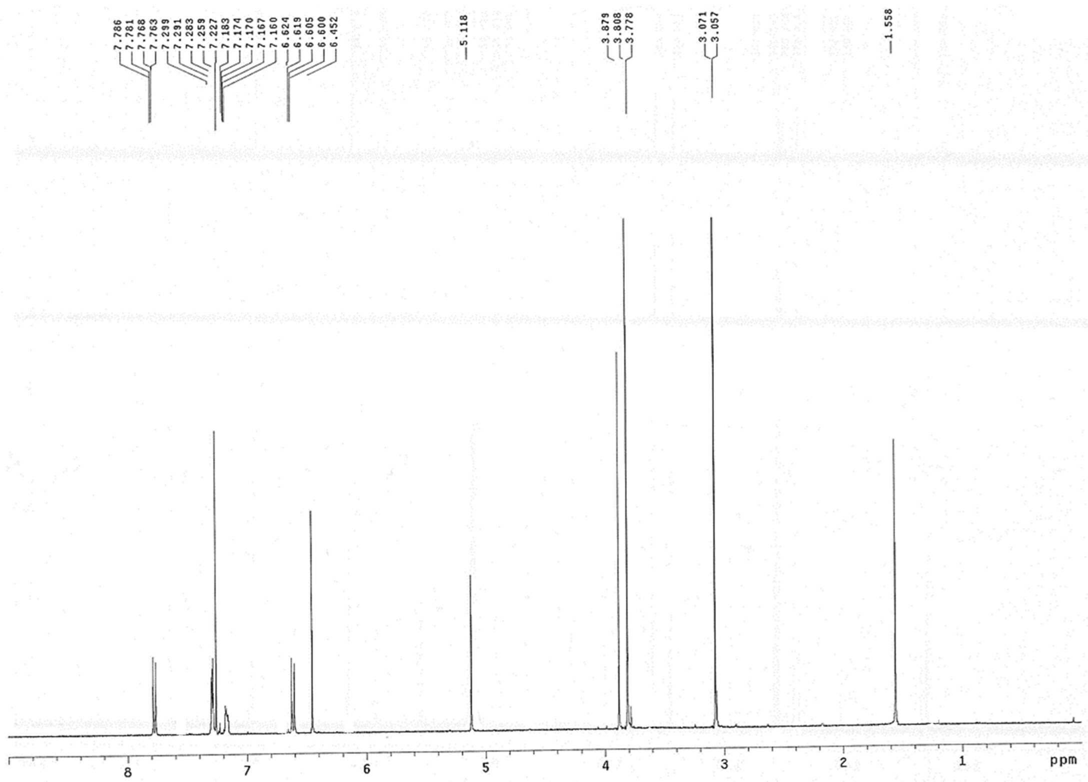

Fig. 39a: Compound **35b** ^{1}H NMR spectra.

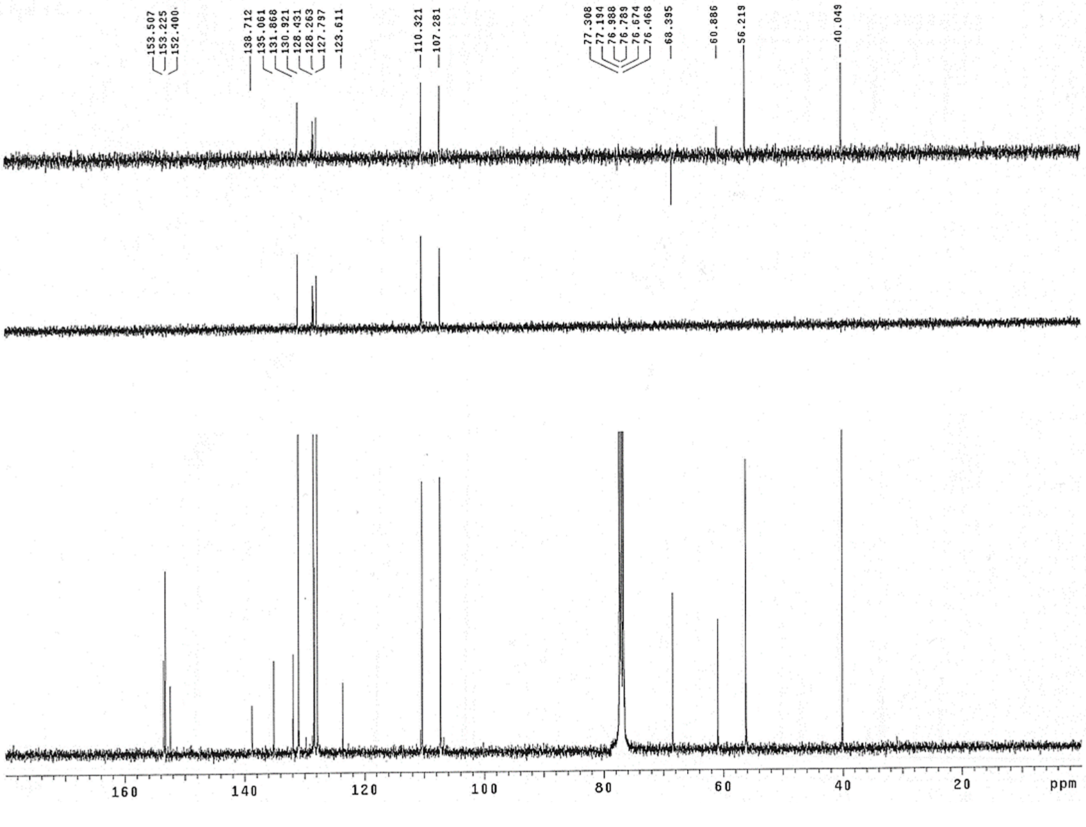

Fig. 39b: Compound **35b** ^{13}C NMR spectra.

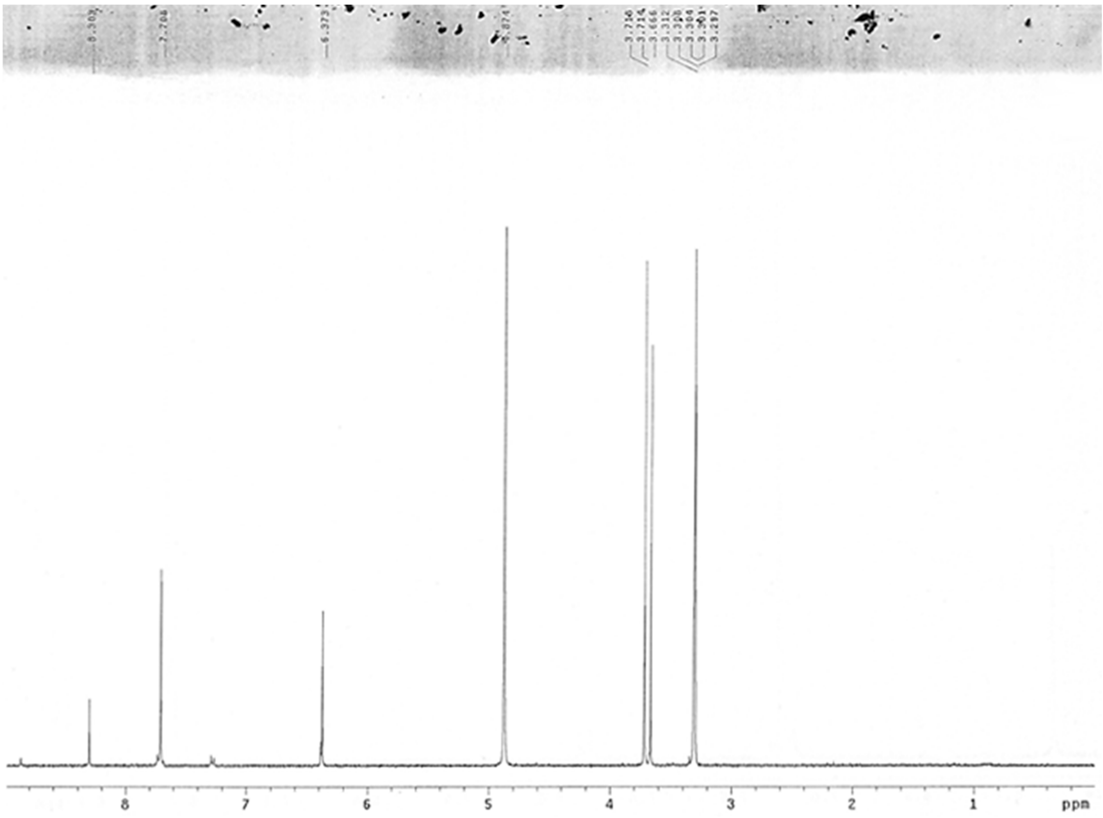

Fig. 40a: Compound **36** ^{1}H NMR spectra.

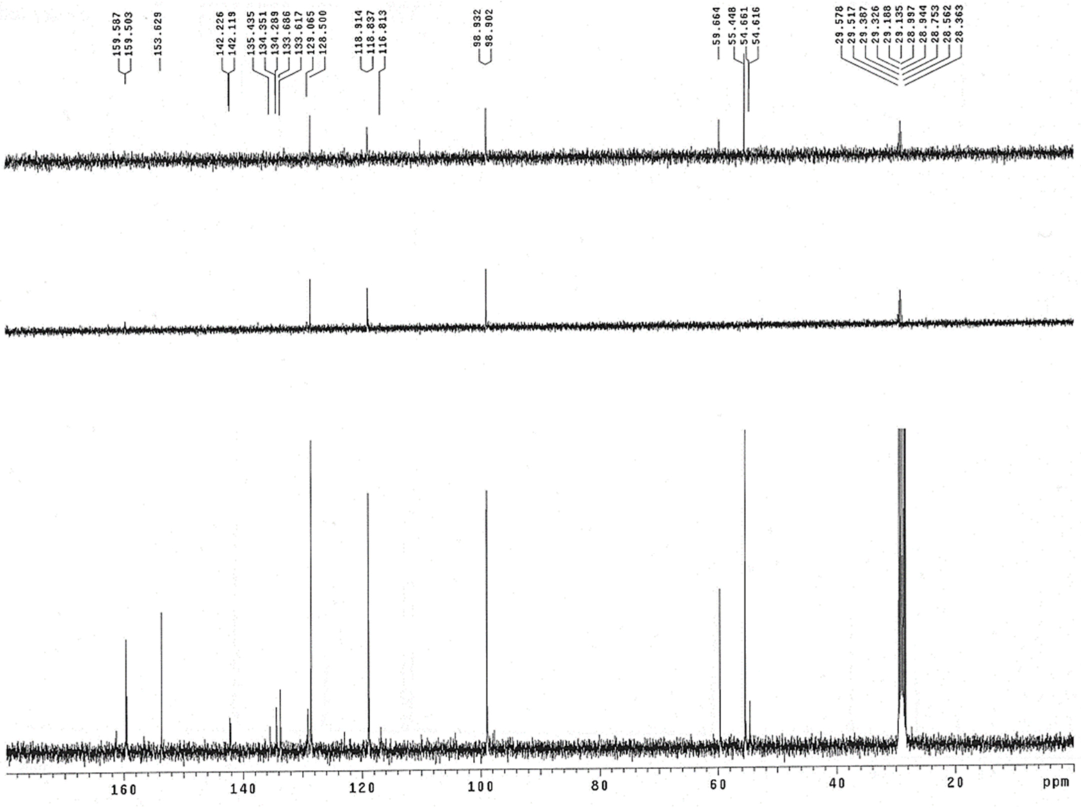

Fig. 40b: Compound **36** ^{13}C NMR spectra.

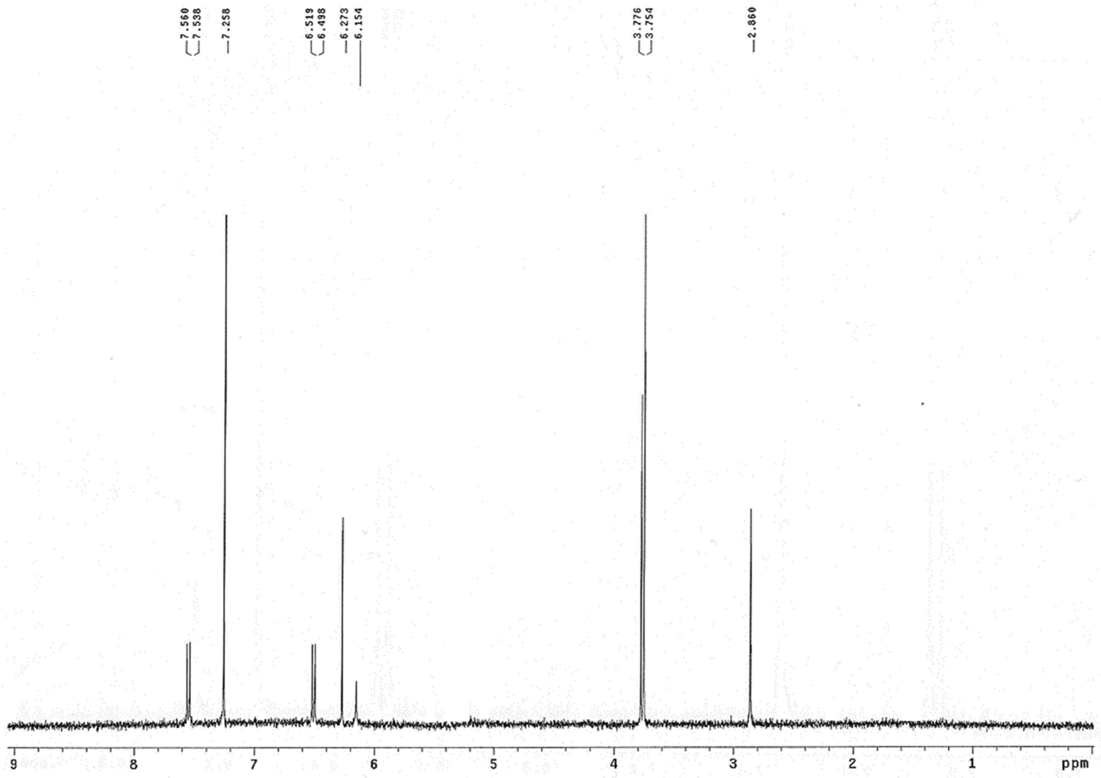

Fig. 41a: Compound **37** ^{1}H NMR spectra.

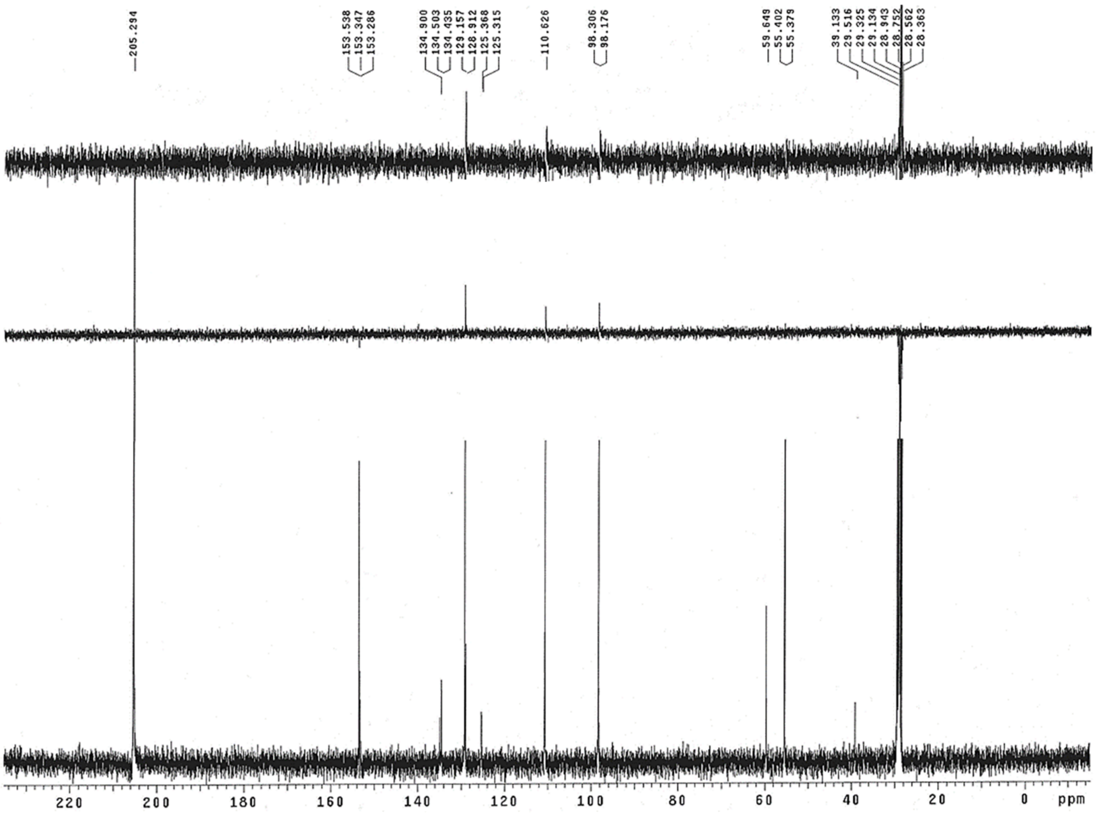

Fig. 41b: Compound **37** ^{13}C NMR spectra.

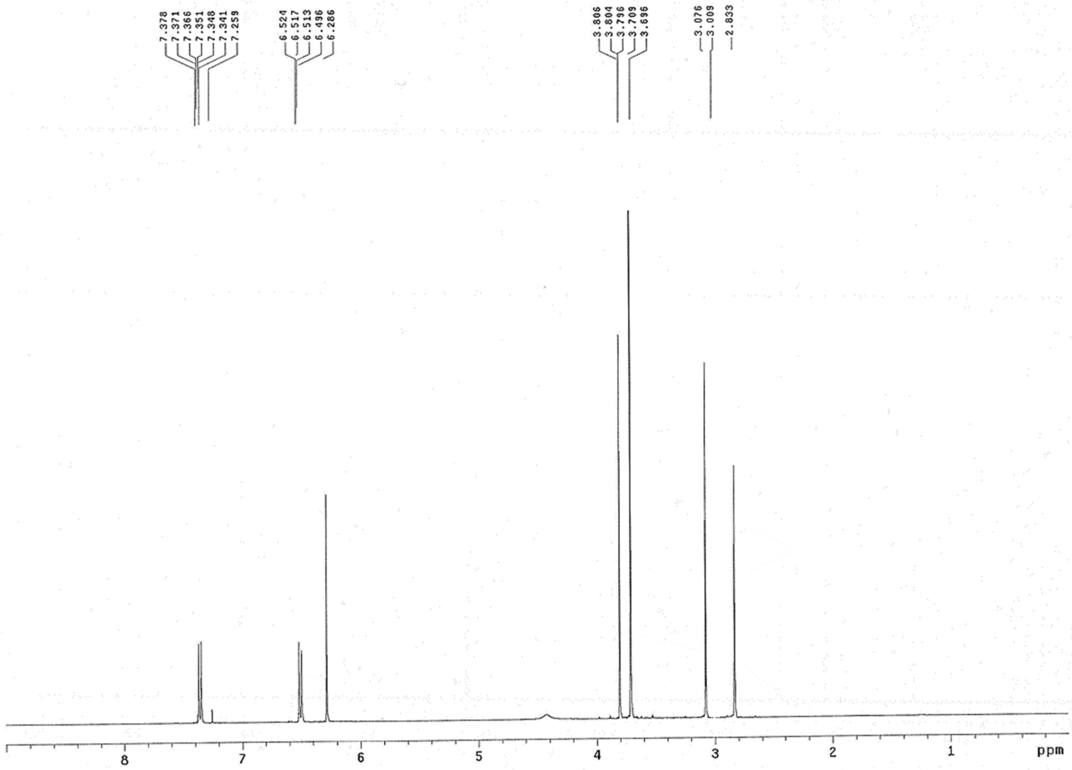

Fig. 42a: Compound **38** ^{1}H NMR spectra.

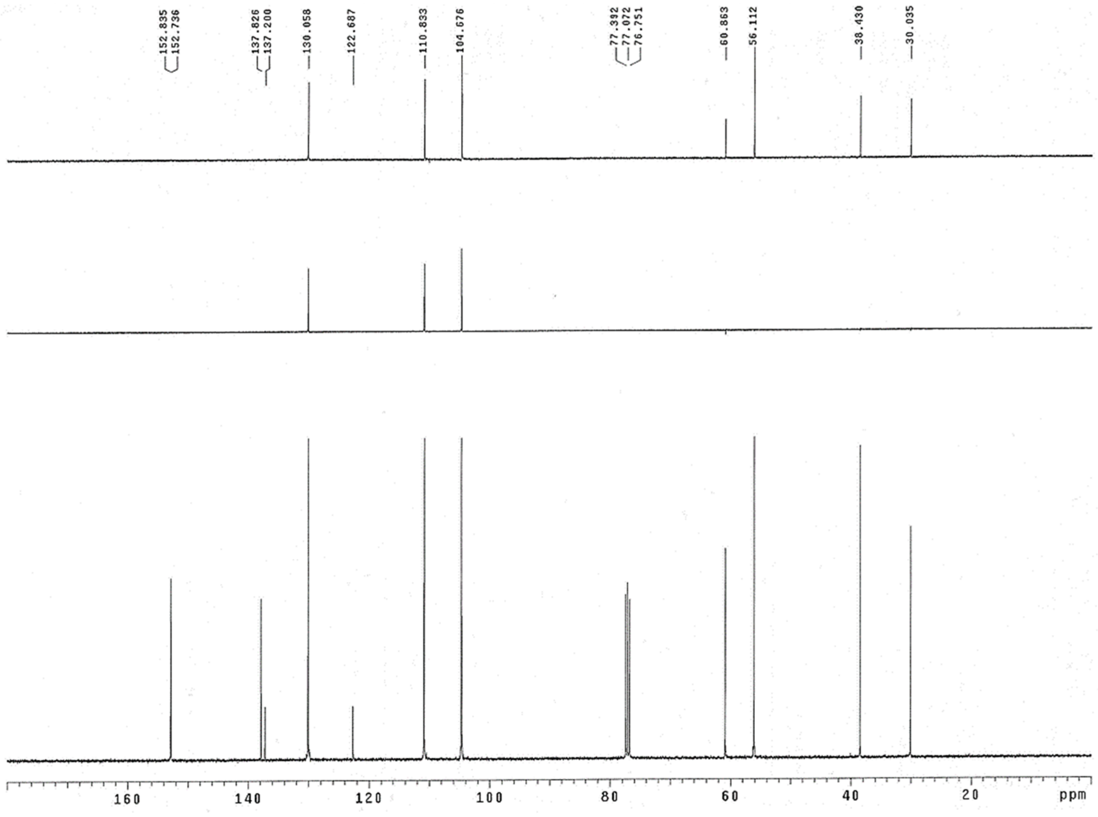

Fig. 42b: Compound **38** ^{13}C NMR spectra.

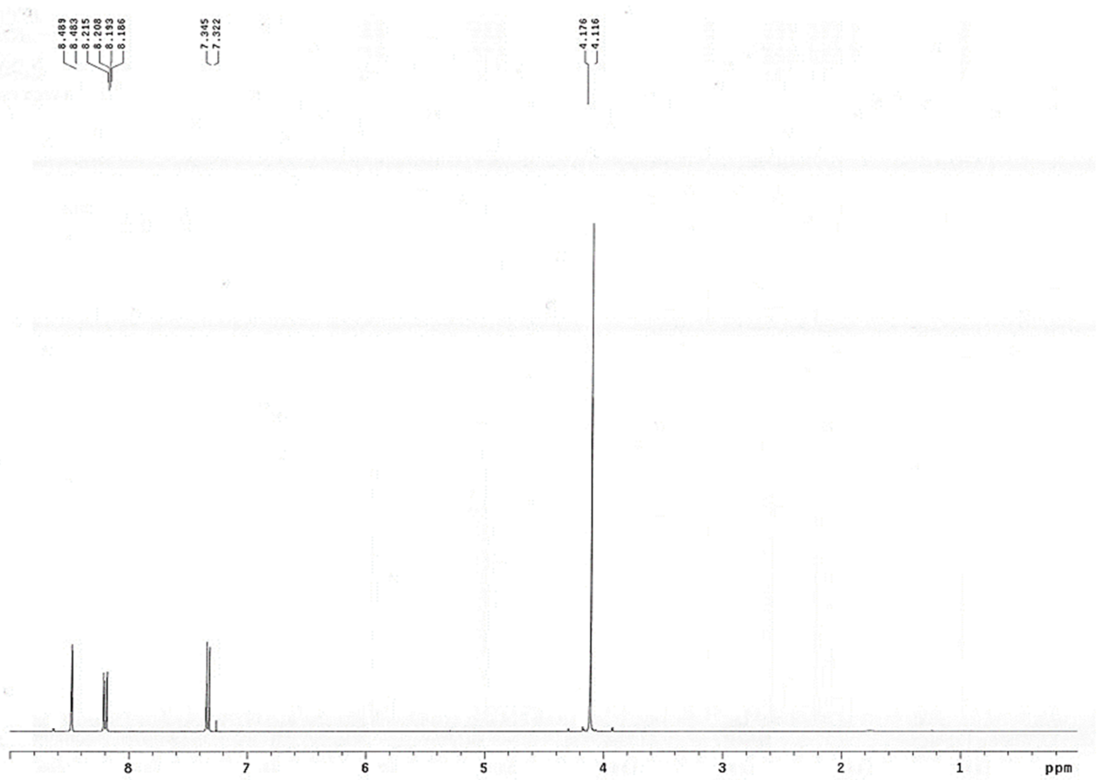

Fig. 43a: Compound **39** ^{1}H NMR spectra.

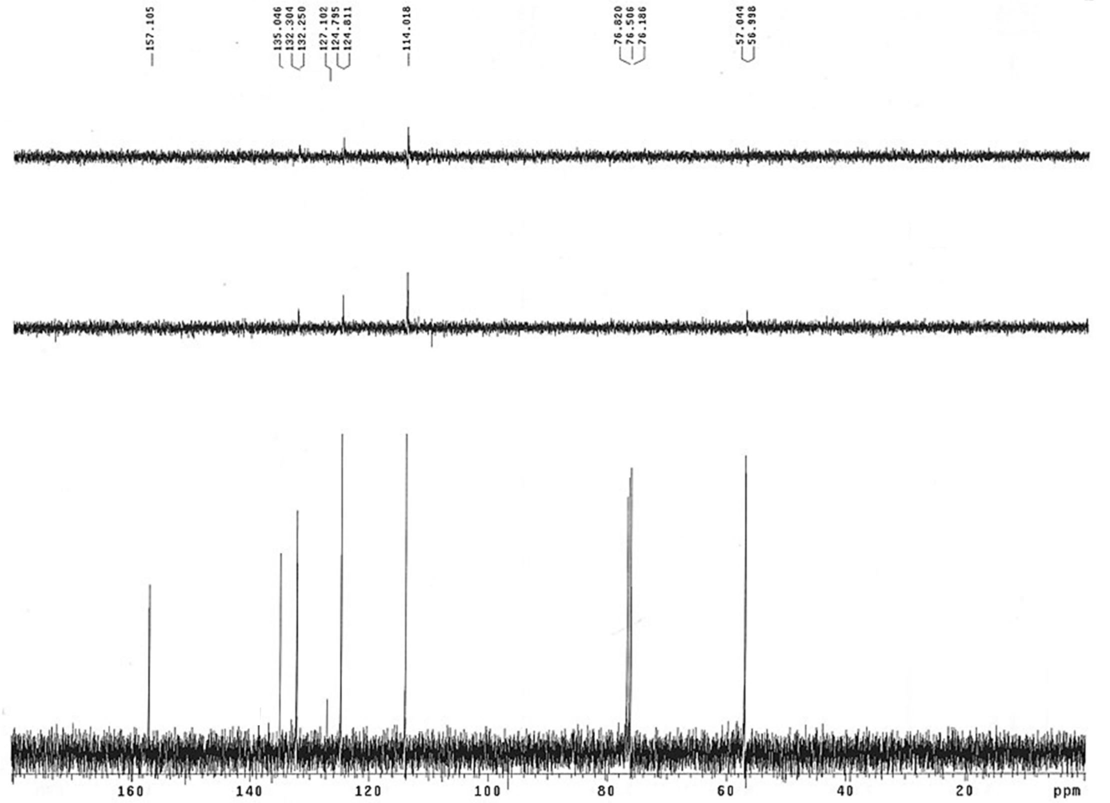

Fig. 43b: Compound **39** ^{13}C NMR spectra.

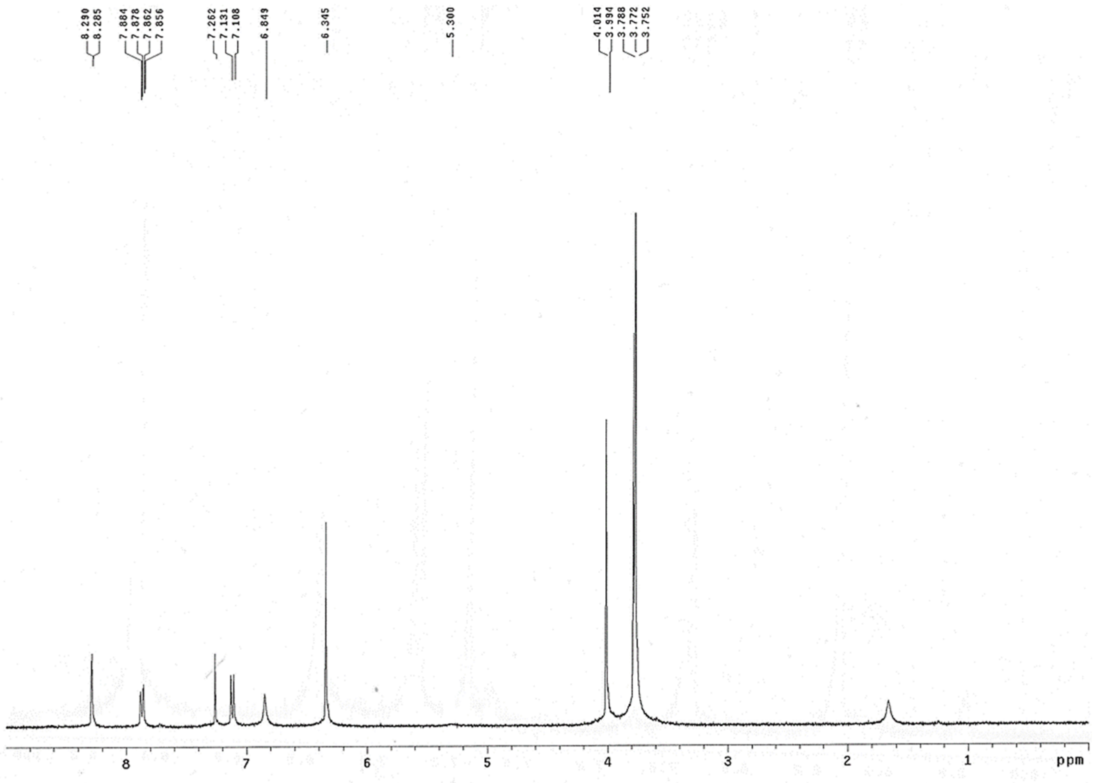

Fig. 44a: Compound **40** ^{1}H NMR spectra.

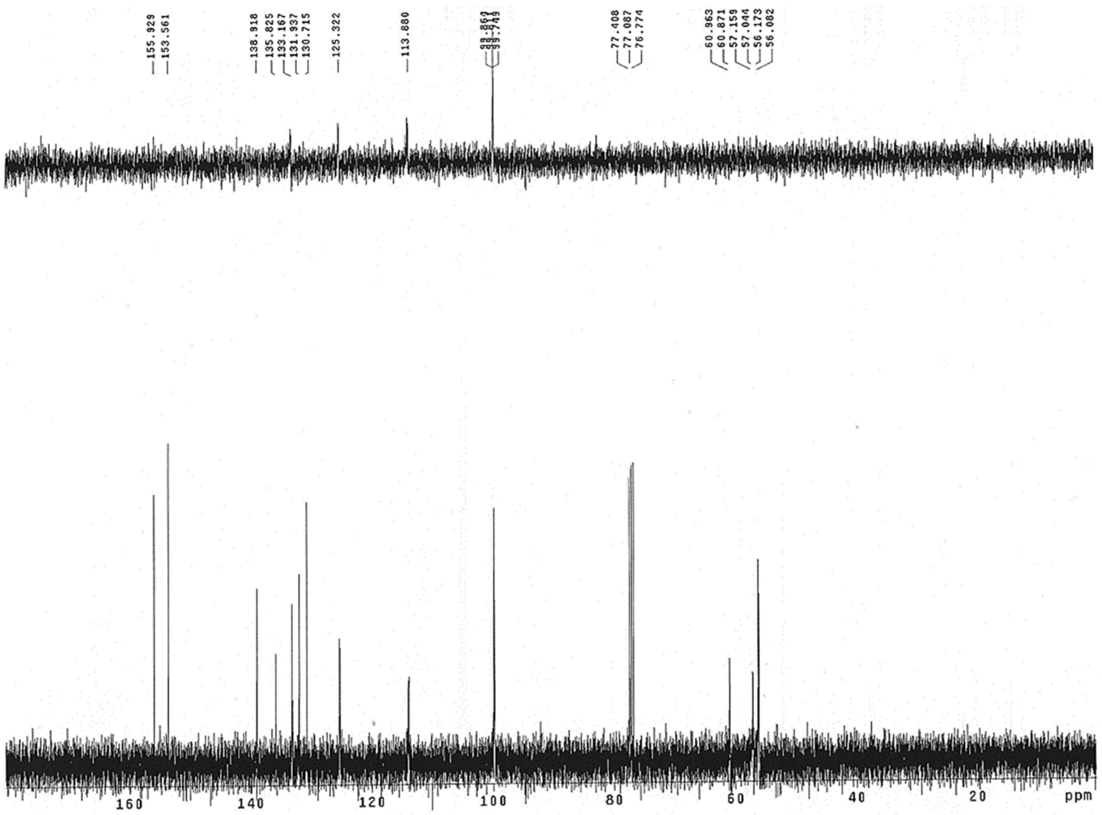

Fig. 44b: Compound **40** ^{13}C NMR spectra.

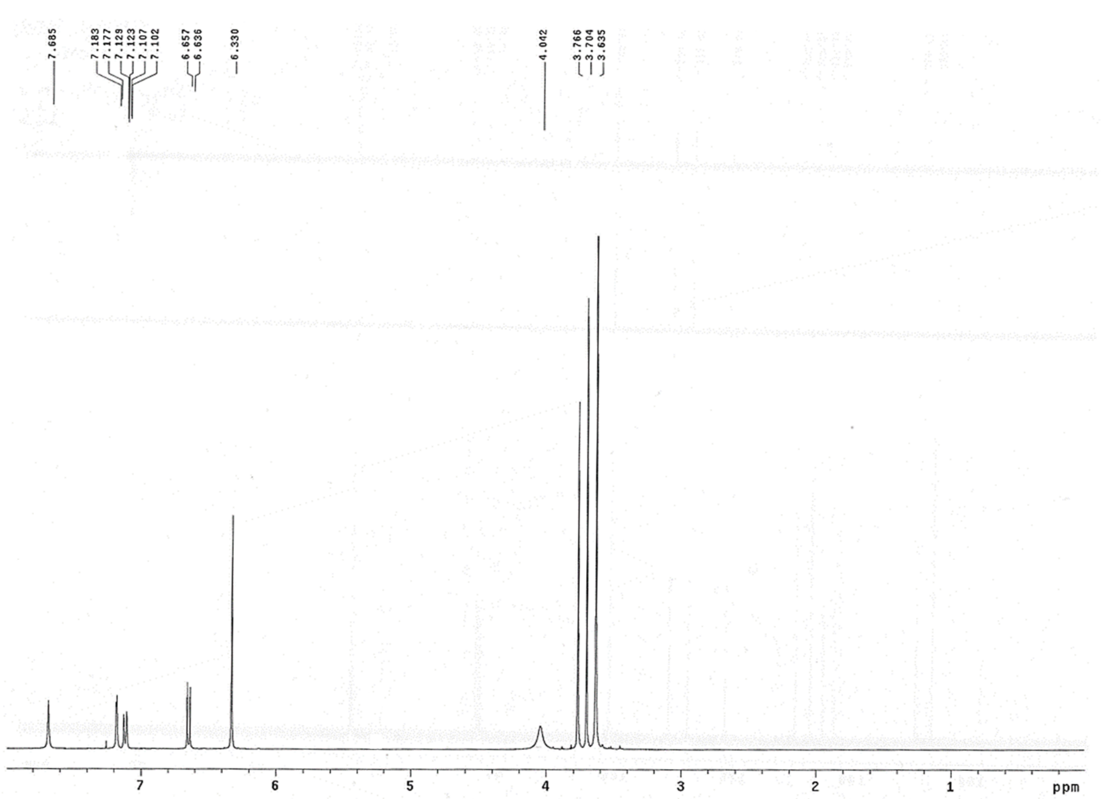

Fig. 45a: Compound **41** ^{1}H NMR spectra.

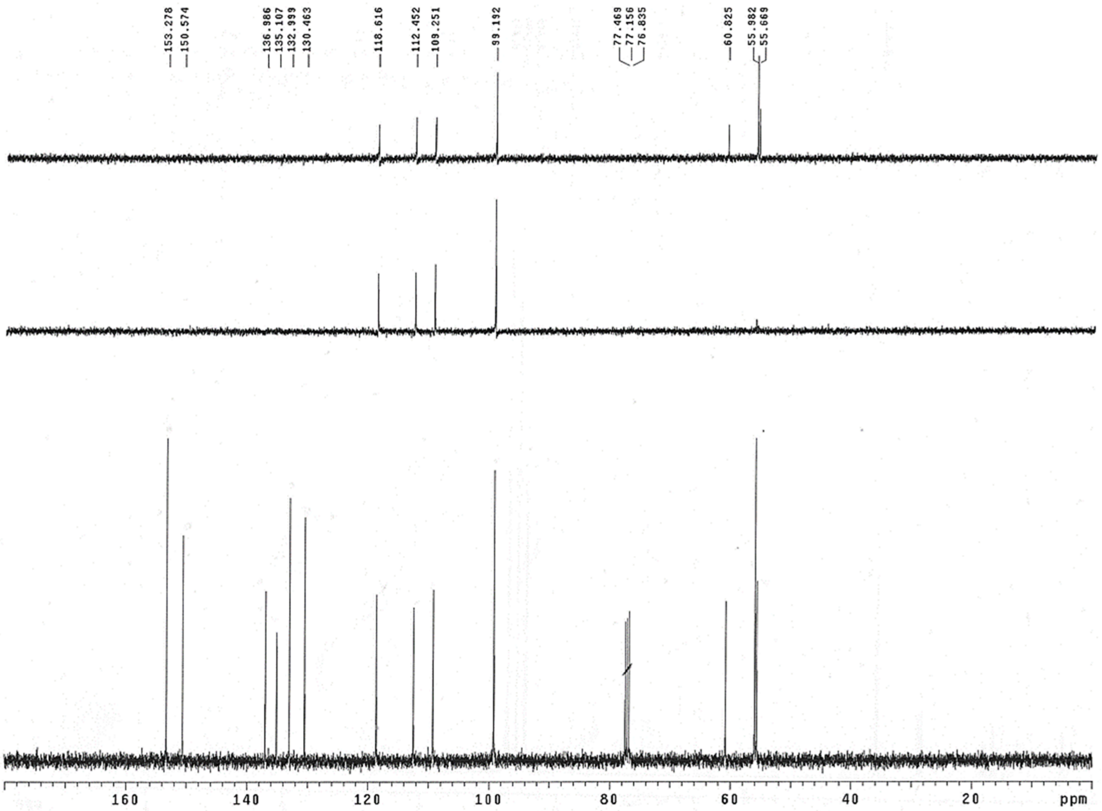

Fig. 45b: Compound **41** ^{13}C NMR spectra.

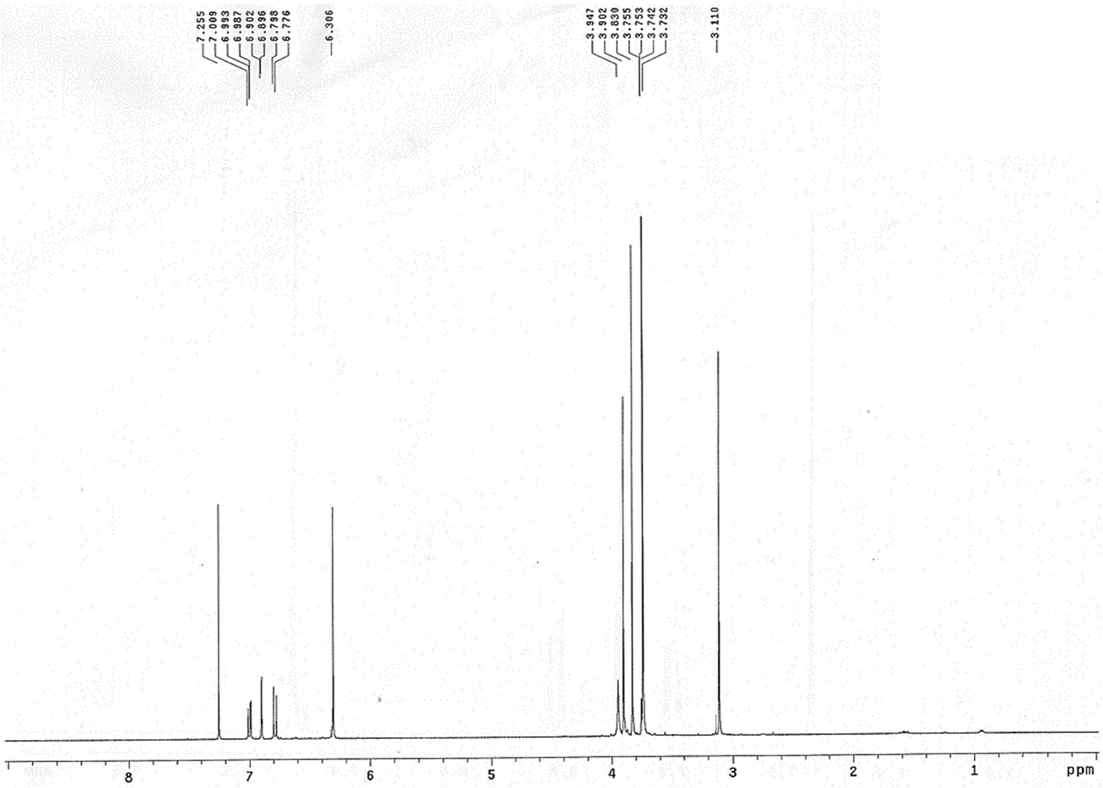

Fig. 46a: Compound **42** ^{1}H NMR spectra.

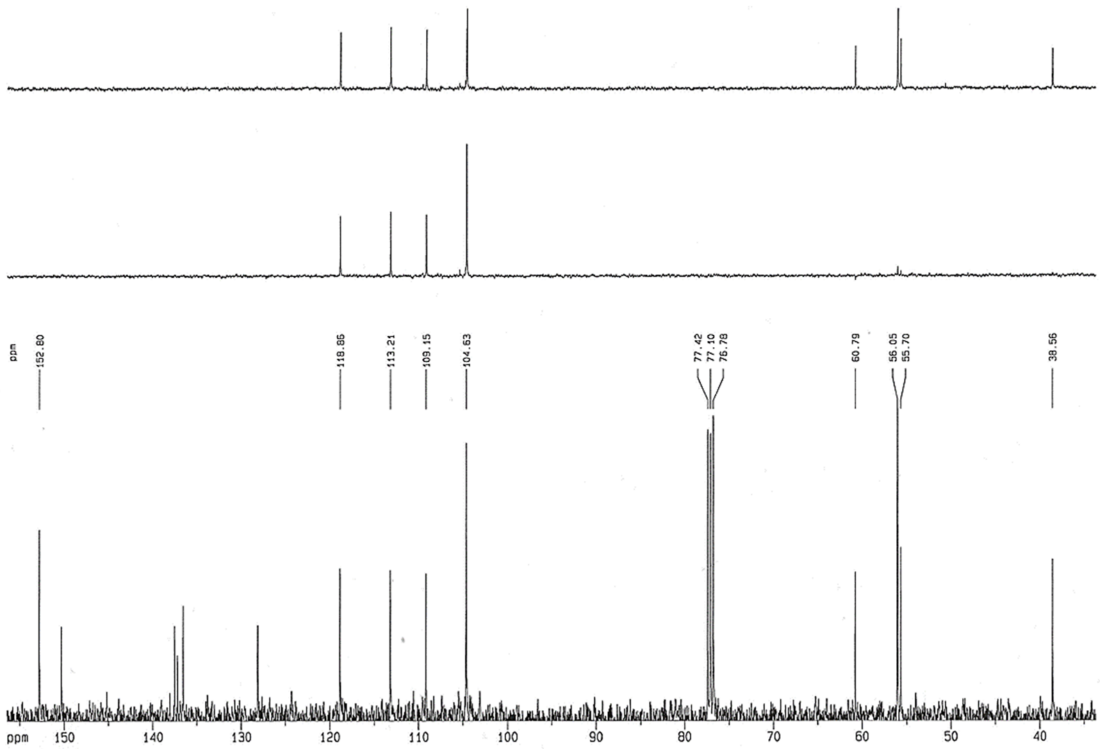

Fig. 46b: Compound **42** ^{13}C NMR spectra.

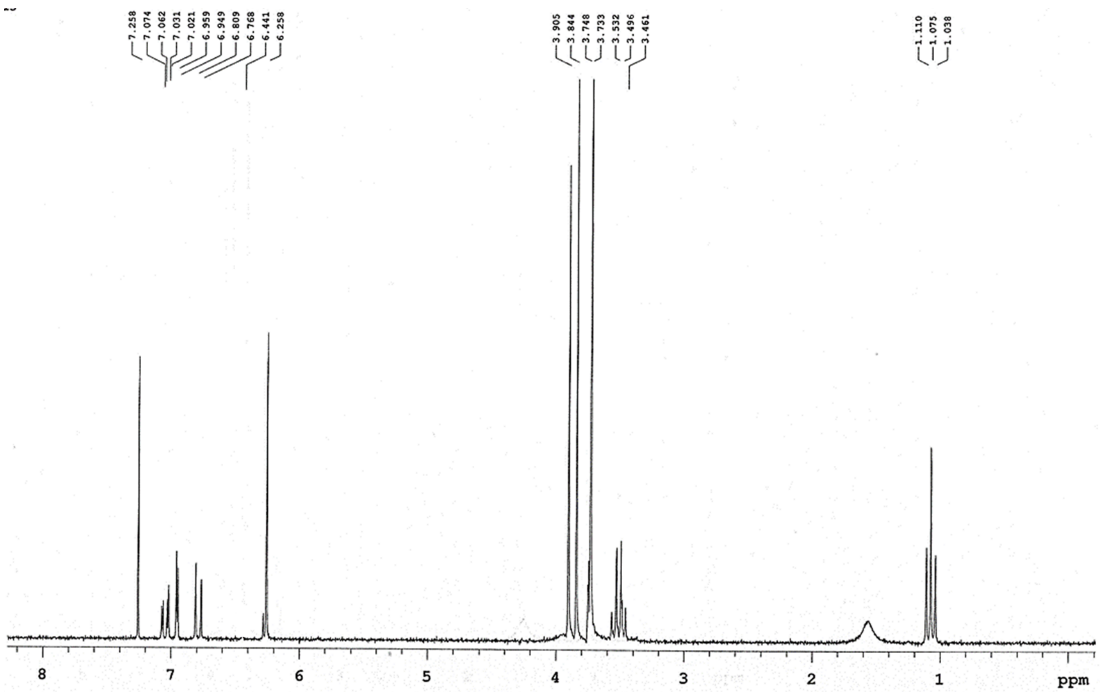

Fig. 47a: Compound **43** ^{1}H NMR spectra.

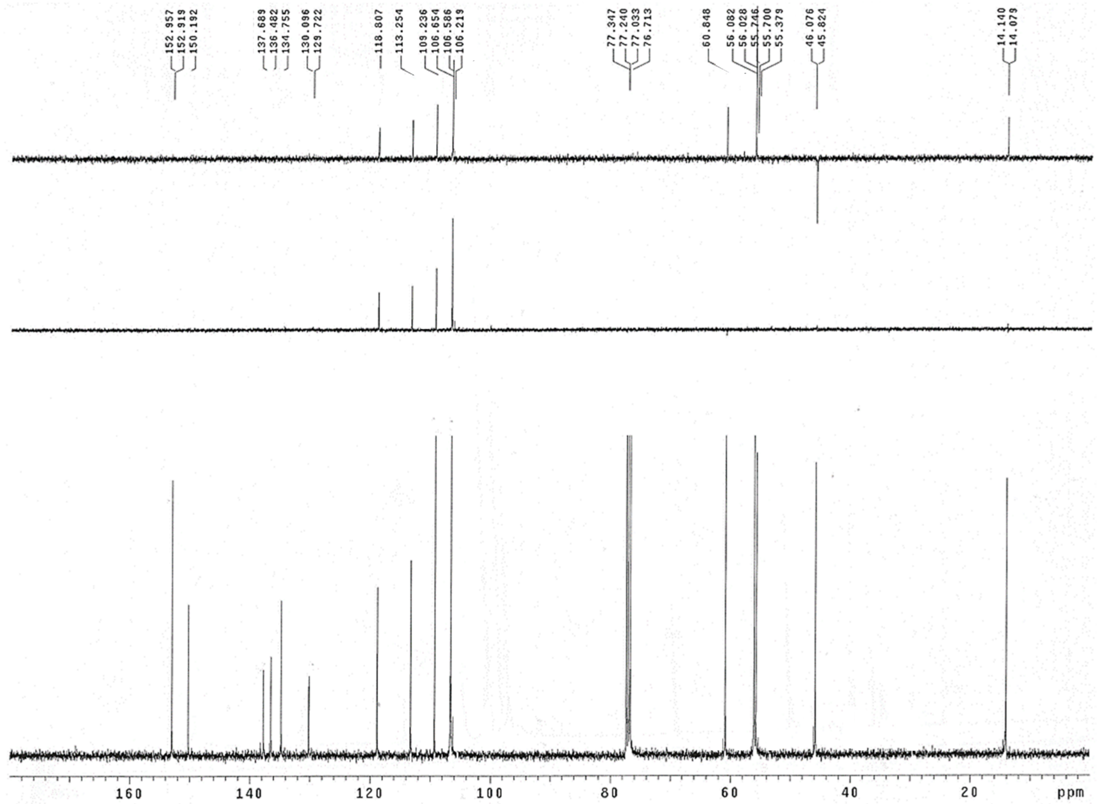

Fig. 47b: Compound **43** ^{13}C NMR spectra.

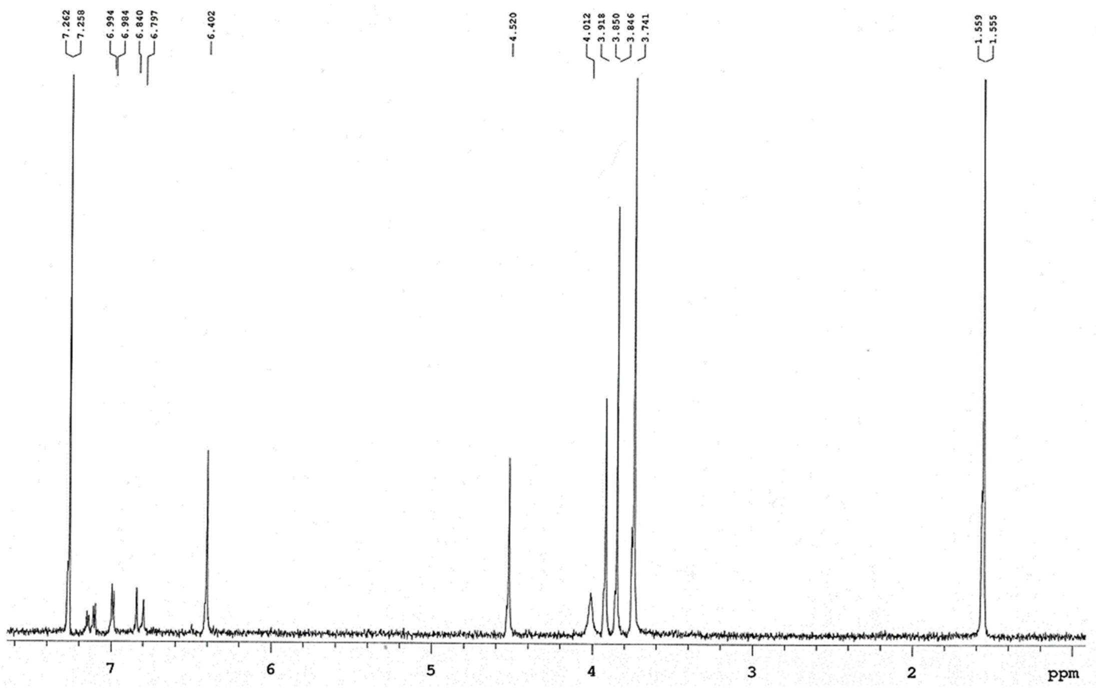

Fig. 48a: Compound **44** ^{1}H NMR spectra.

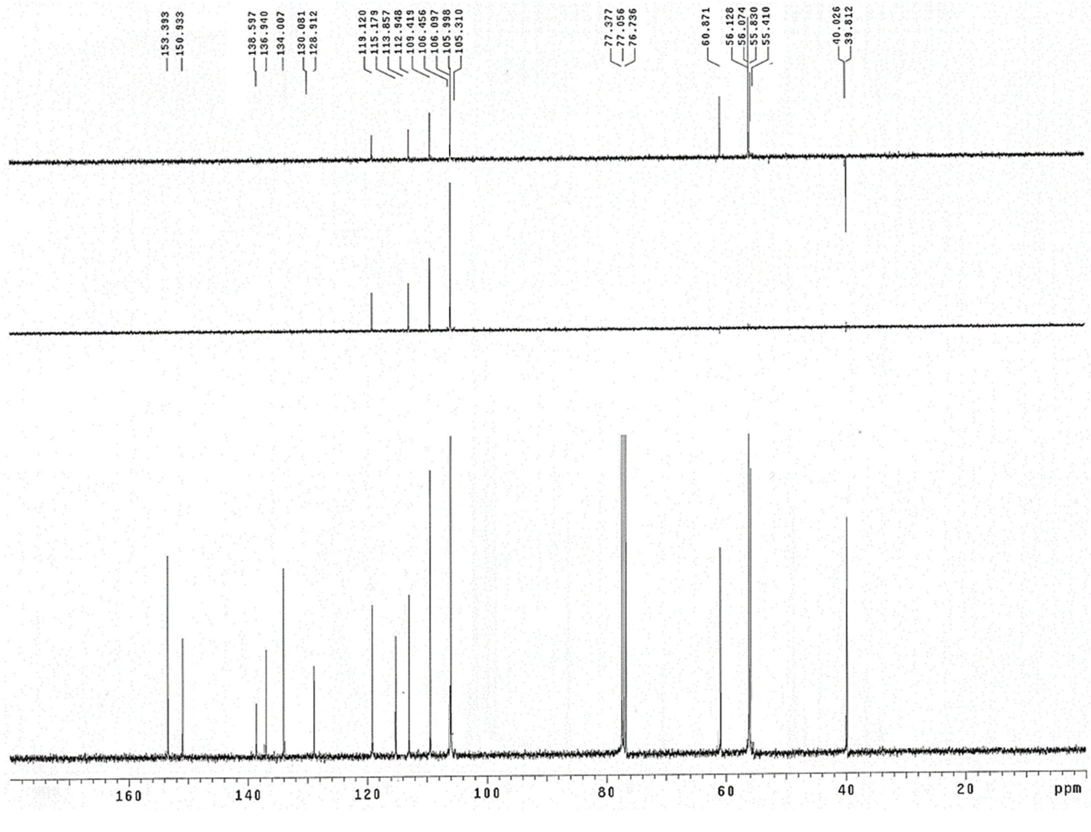

Fig. 48b: Compound **44** ^{13}C NMR spectra.

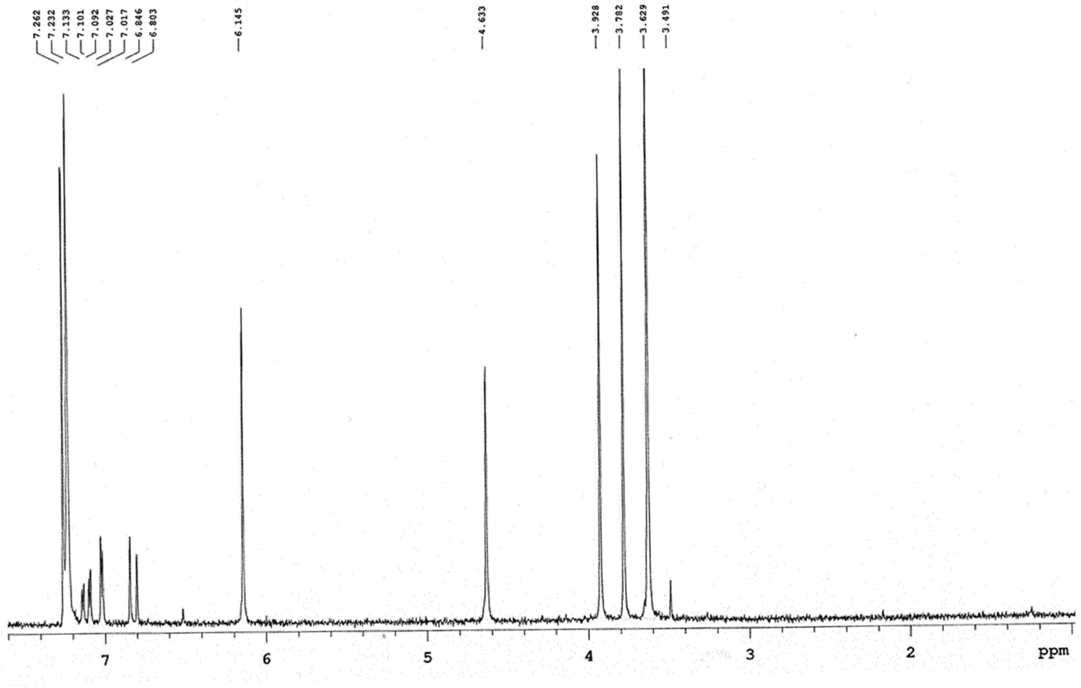

Fig. 49a: Compound **45** ^{1}H NMR spectra.

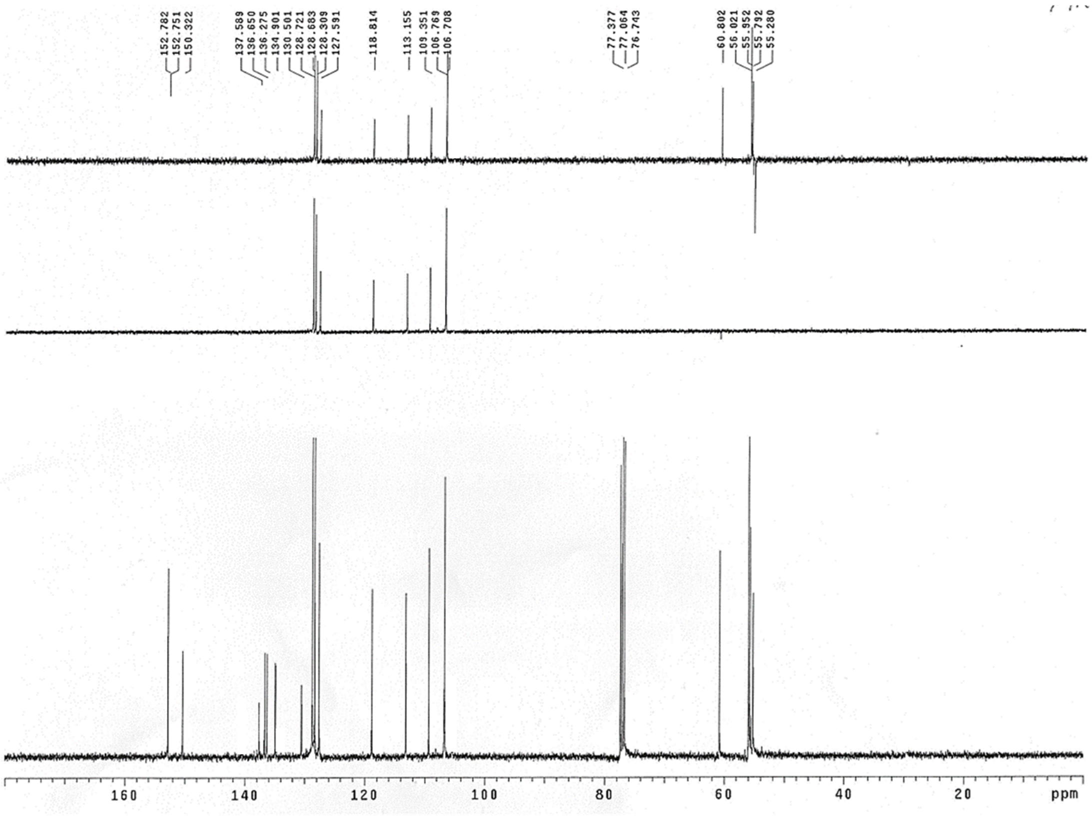

Fig. 49b: Compound **45** ^{13}C NMR spectra.

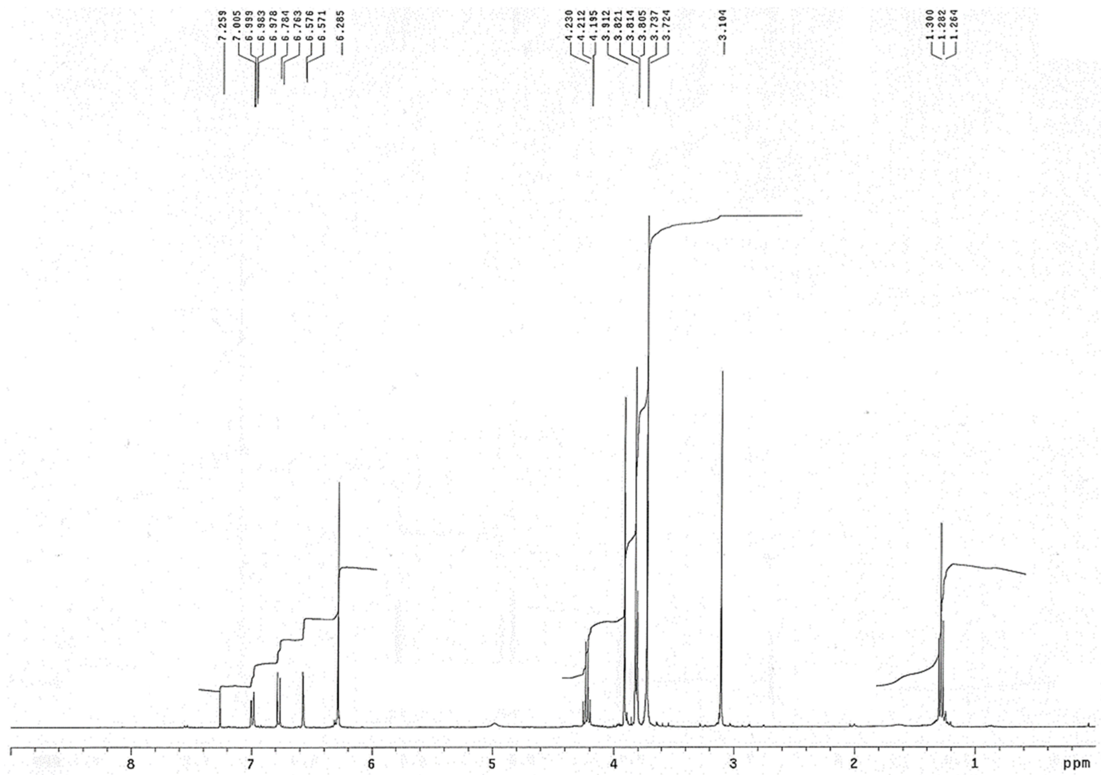

Fig. 50a: Compound **46** ^{1}H NMR spectra.

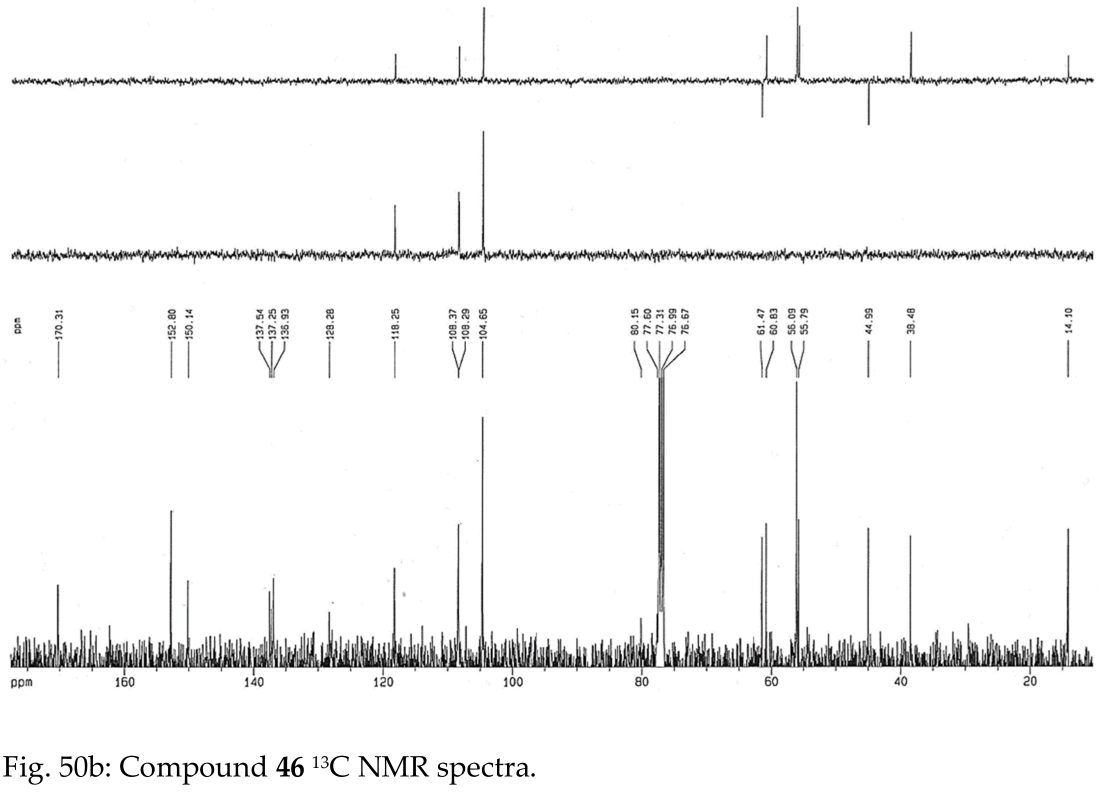

Fig. 50b: Compound **46** ^{13}C NMR spectra.

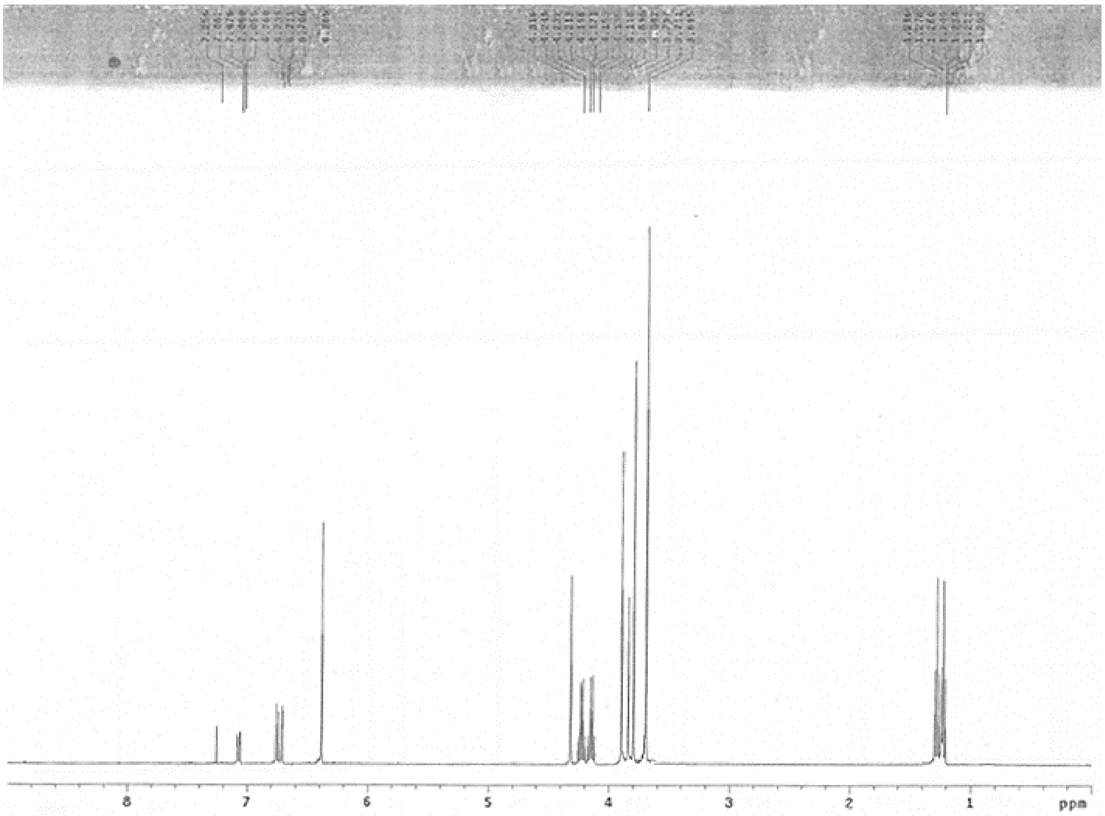

Fig. 51a: Compound **47** ^{1}H NMR spectra.

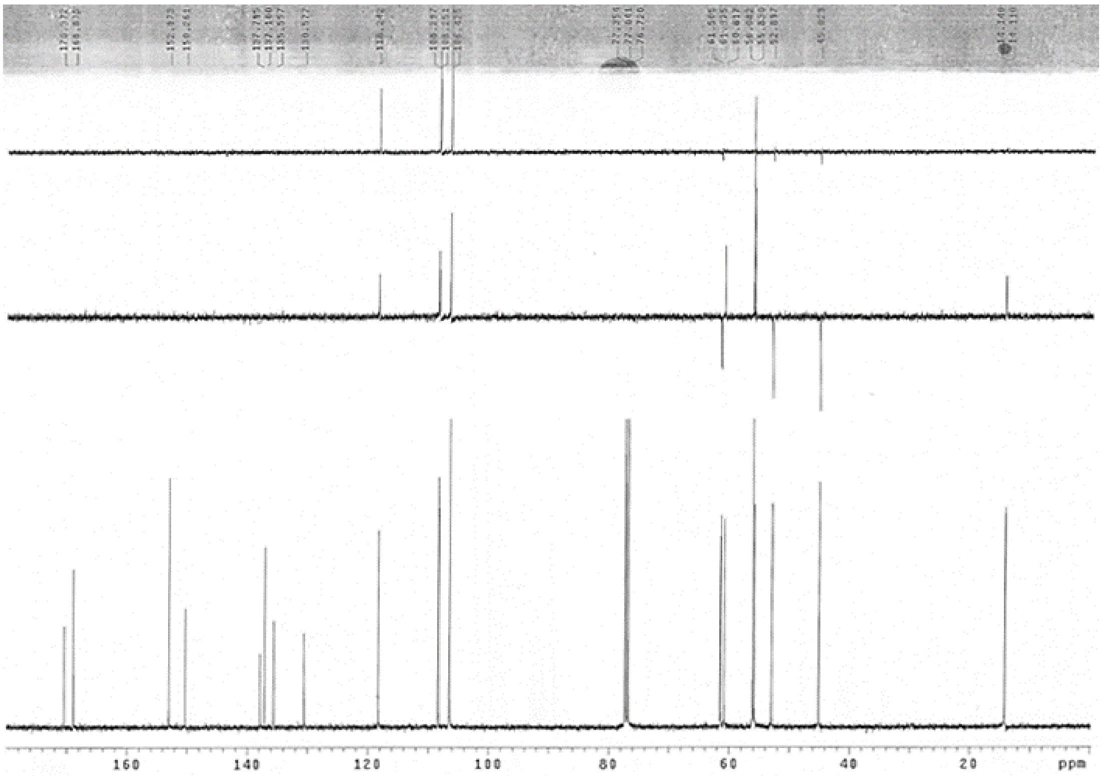

Fig. 51b: Compound **47** ^{13}C NMR spectra.

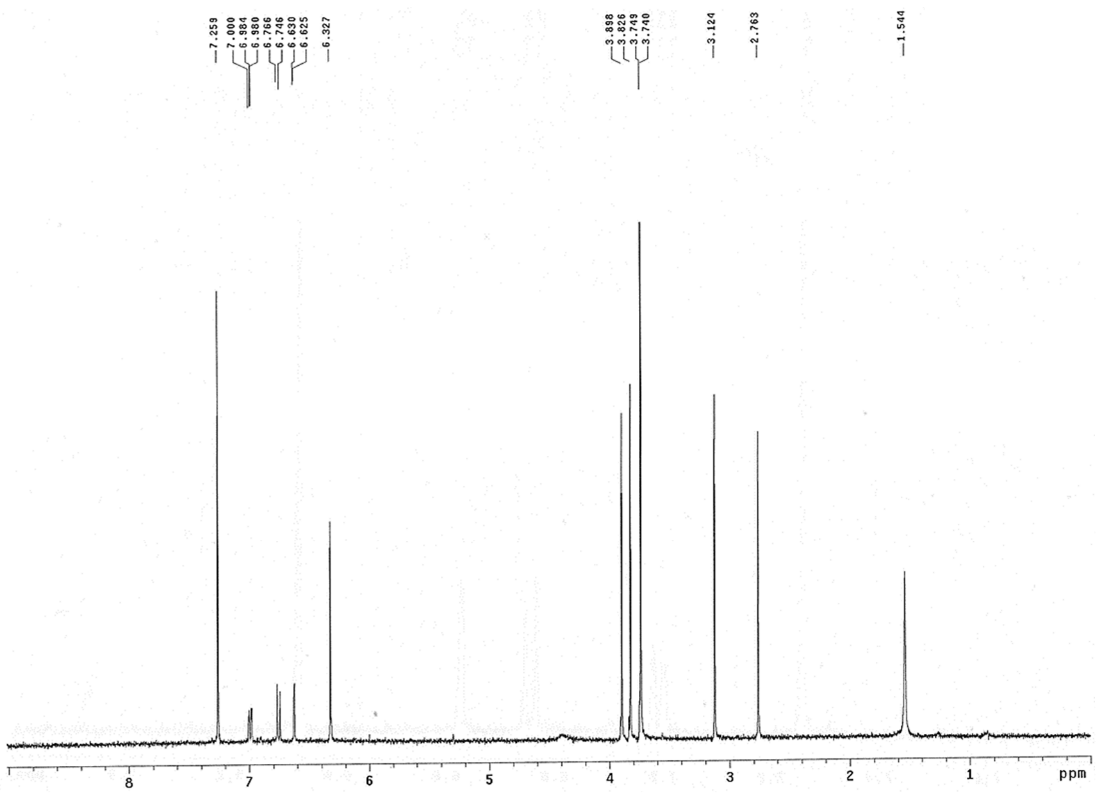

Fig. 52a: Compound **48a** ^{1}H NMR spectra.

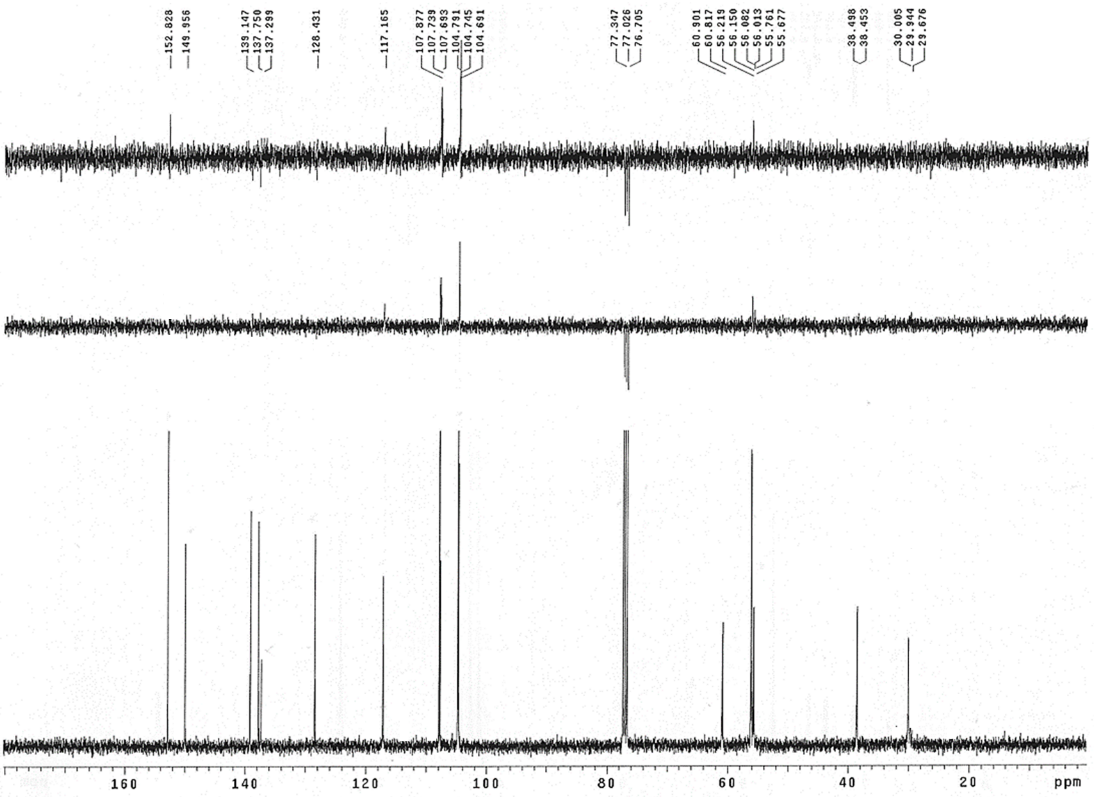

Fig. 52b: Compound **48a** ^{13}C NMR spectra.

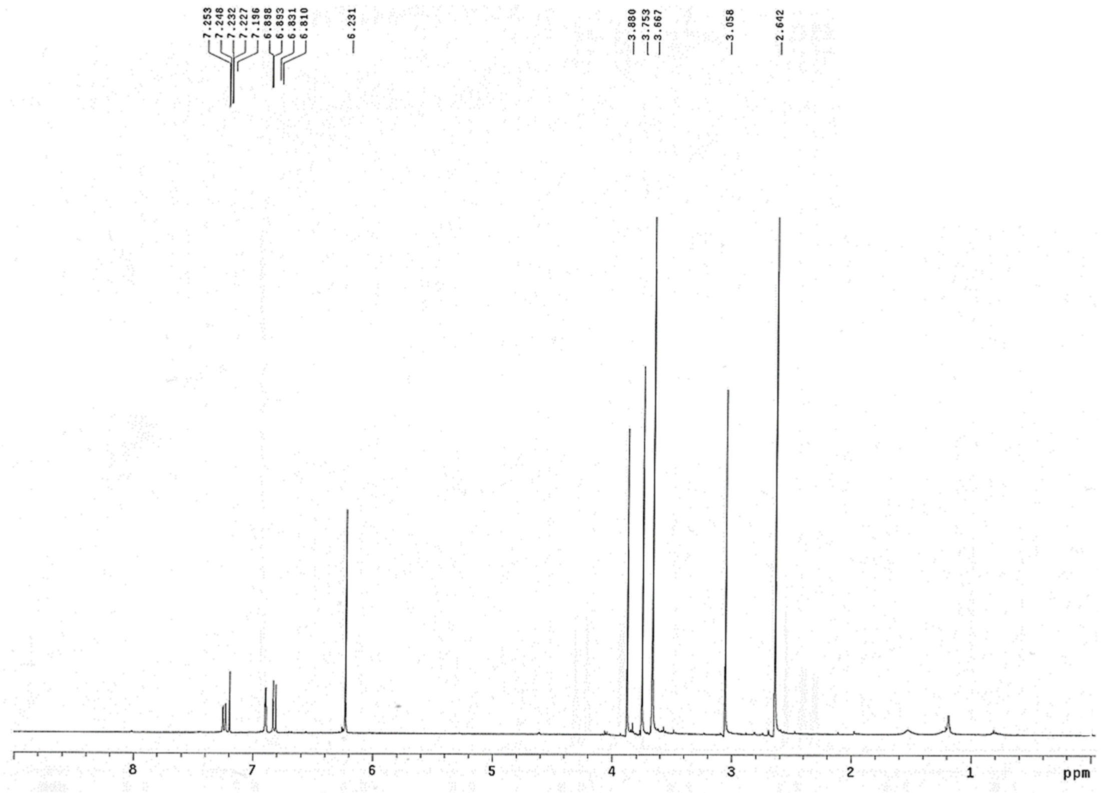

Fig. 53a: Compound **48b** ^{1}H NMR spectra.

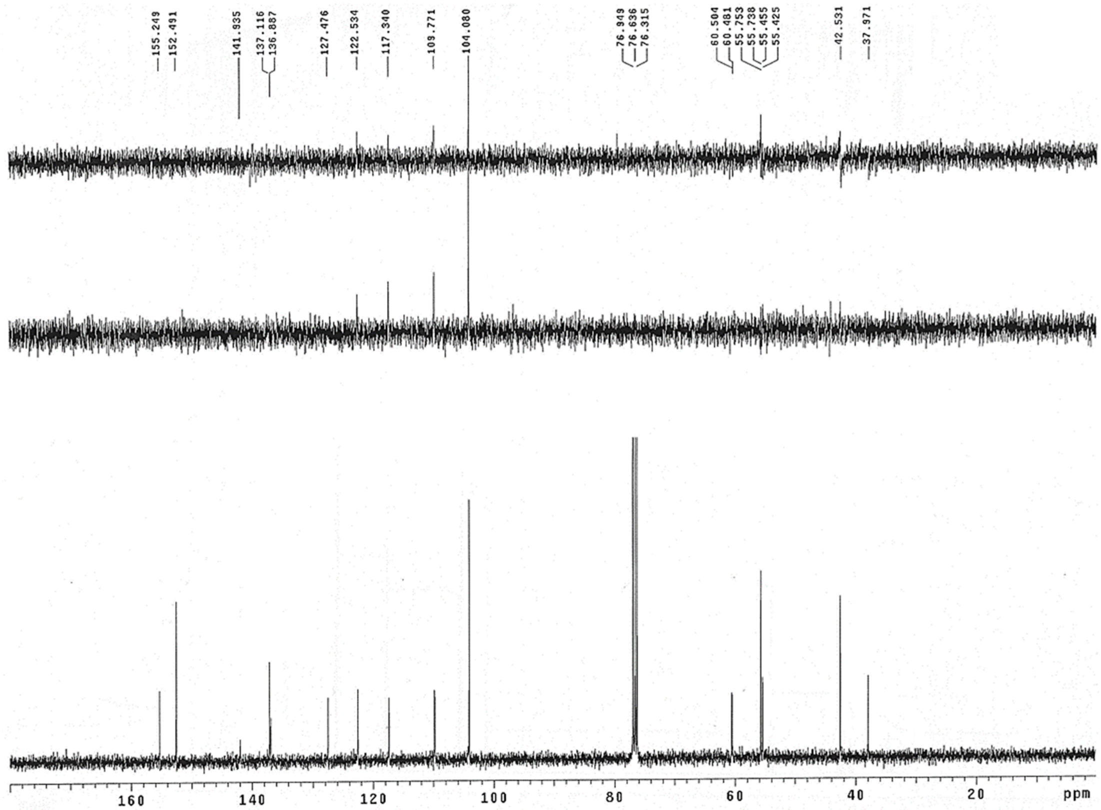

Fig. 53b: Compound **48b** ^{13}C NMR spectra.
